# Supplementary material for: Optimizing the conversion of phosphoenolpyruvate to lactate by enzymatic channeling with mixed nanoparticle display
Source: Cell Rep Methods. 2024 May 6;4(5):100764. doi: 10.1016/j.crmeth.2024.100764 (PMC11133815; doi:10.1016/j.crmeth.2024.100764)
Supplement: Document S1. Figures S1–S14 and Tables S1 and S2 [file mmc1.pdf]

Cell Reports Methods, Volume *4*

Supplemental information

Optimizing the conversion of phosphoenolpyruvate to lactate by enzymatic channeling with mixed nanoparticle display

Shelby L. Hooe, Christopher M. Green, Kimihiro Susumu, Michael H. Stewart, Joyce C. Breger, and Igor L. Medintz

Supporting Information

Optimizing the Conversion of Phosphoenolpyruvate to Lactate by Enzymatic Channeling with Mixed Nanoparticle Display

Shelby L. Hooe,[a] Christopher M. Green,[a] Kimihiro Susumu,[b] Michael H. Stewart,[b] Joyce C. Breger,[a] and Igor L. Medintz[a*]

[a]Center for Bio/Molecular Science and Engineering Code 6900
[b]Optical Sciences Division, Code 5611
U.S. Naval Research Laboratory
Washington, D.C. 20375, USA

*Email: igor.medintz@nrl.navy.mil

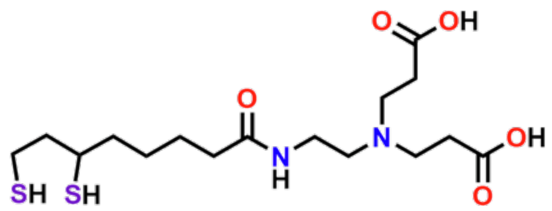

Figure S1. Structure of CL4 ligand shown with the thiols open as the dithiolane (related to Figure 1C). This ligand on the QD surface enables colloidal stability of enzyme-QD mixtures.

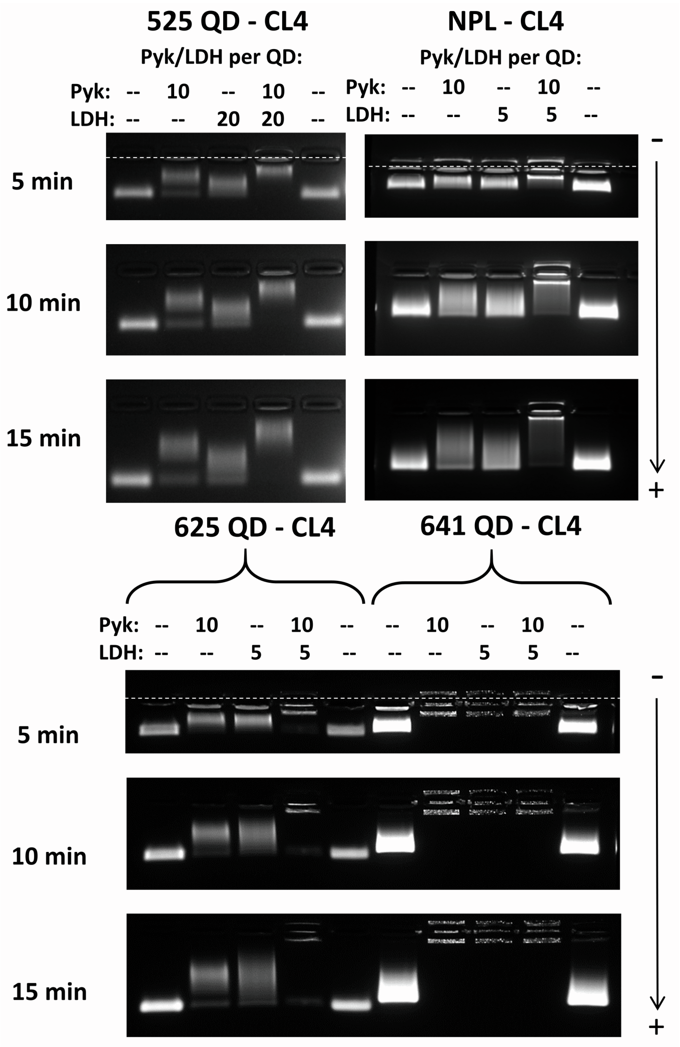

Figure S2. Characterization of QD enzyme clusters. This data supports the gel data shown in Figure 2A. ***Top*** - representative agarose gel mobility assay confirming PykA and LDH assembly to the 523 nm emitting QDs and 585 nm NPLs capped with CL4 ligand. 2.5 picomoles of nanoparticle/well were assembled with the indicated ratio of PykA and/or LDH enzyme and then separated in a 1.5% and 1.0% agarose gel for the 523 nm QD and 585 nm NPLs, respectively, supplemented with 1×TBE buffer. The degree of nanoparticle mobility shifting correlates to the increased ratio of enzyme displayed on their surface. ***Bottom*** - representative agarose gel mobility assay confirming PykA and LDH assembly to the 625 nm and 641 nm emitting QDs capped with CL4 ligand. 2.5 picomoles of QD/well were assembled with the indicated ratio of PykA and/or LDH enzyme and then separated in a 1.0% agarose gel for the supplemented with 1×TBE buffer. The degree of nanoparticle mobility shifting correlates to the increased ratio of enzyme displayed on their surface. The degree of QD mobility is directly correlated to the ratio of different enzymes displayed on their surface. Ratios of enzyme used for gel assays do not correlate to the empirically estimated number of each enzymes that fit around the QD but are utilized to improve differences in mobility. Excess enzyme is used since the sieving action of the gel can remove loosely associated enzyme on the QDs. The location of the wells are indicated by the white dashed line in the 5 minute images.

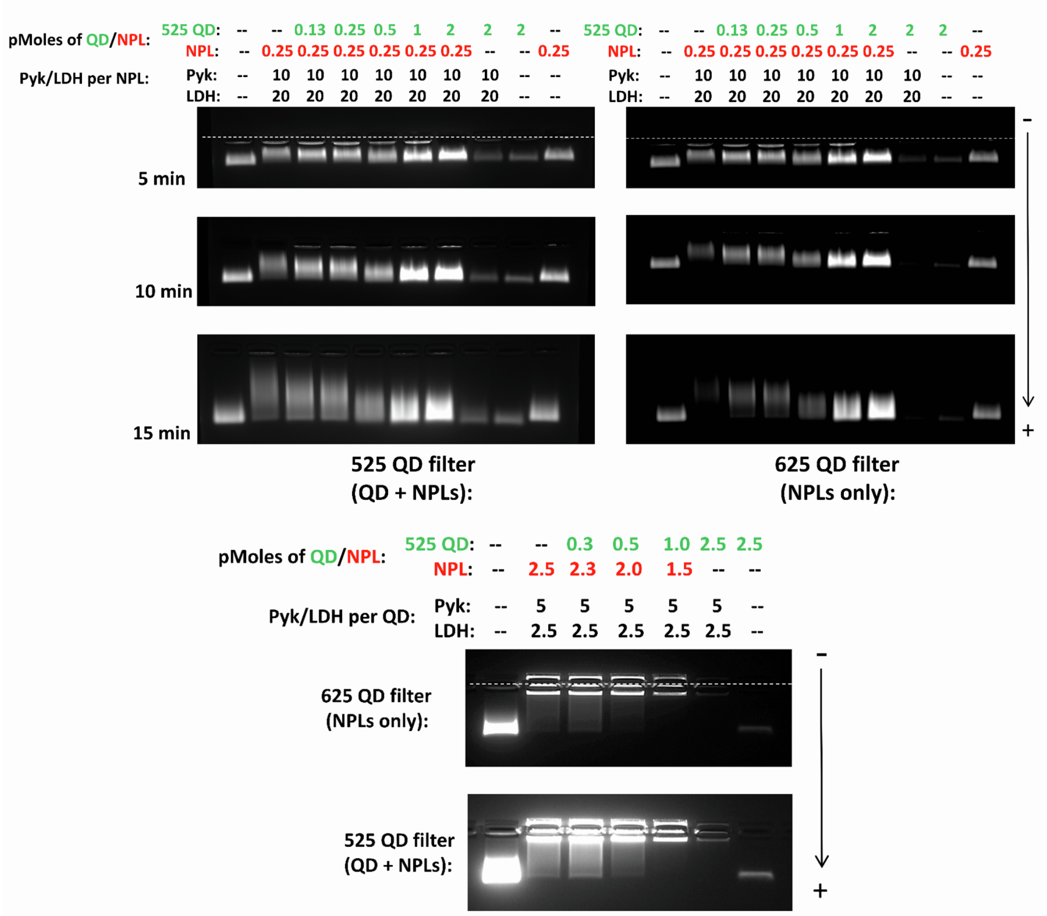

Figure S3. Agarose gels of mixed NP assembly. This data supports the gel data shown in Figure 2A. (Top) Agarose gel mobility assay confirming assembly of the PykA→LDH 2 enzyme cascade to 525 nm emitting QDs and NPLs capped with CL4 ligand. Gel in 2.0% agarose 1×TBE buffer. Enzymes added to QDs and/ NPLs in their ratios shown. These ratios are different from those used in the catalytic assays and were arrived at empirically to reveal changes in mobility during electrophoresis with each sequential nanoparticle addition. (Bottom) Agarose gel mobility assay confirming assembly of the PykA→LDH 2 enzyme cascade to 525 nm emitting QDs and NPLs capped with CL4 ligand. Gel in 2.0% agarose 1×TBE buffer. Enzymes added to QDs and/or NPLs in their ratios shown. These ratios are different from those used in the catalytic assays and were arrived at empirically to reveal changes in mobility during electrophoresis with each sequential nanoparticle addition. White dashed line indicates the location of the wells where samples were loaded.

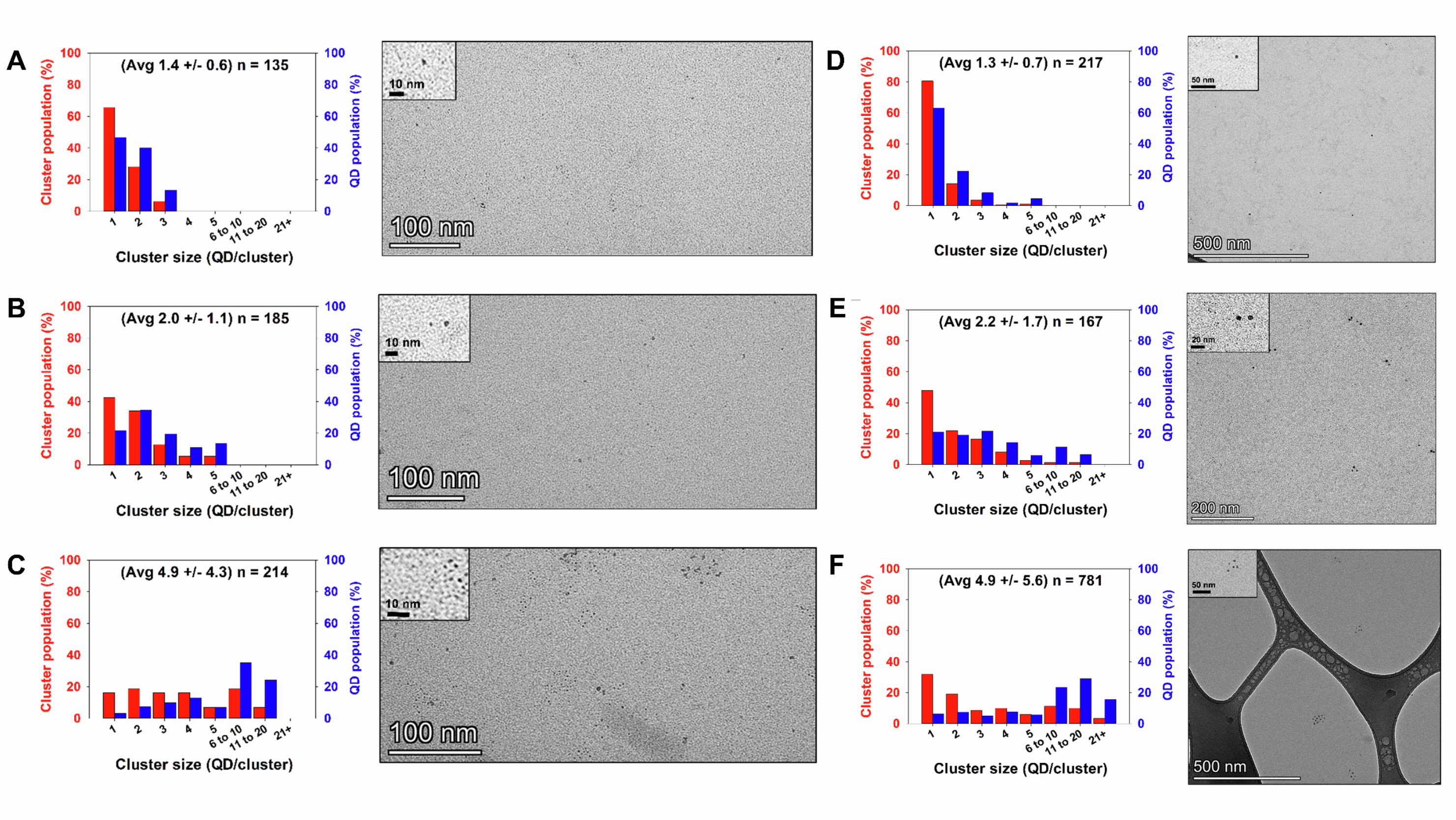

Figure S4. TEM characterization of QD-enzyme clusters with the 525 and 625 QDs. This data supports the TEM data shown in Figure 1C. Representative TEMs of 525 QDs assembled with 40 nM LDH and 20 nM PykA with 0.5 nM **(A)**, 1 nM **(B)**, and 2 nM **(C)** 525 QD. Average cluster size is given above the micrograph along with the number of QDs counted. Corresponding bar plots for each sample below showing the distribution of cluster sizes present (red) and number of NPs per cluster size (blue). TEM characterization of QD-enzyme clusters with the 625 QDs. Representative TEMs of 625 QDs assembled with 40 nM LDH and 20 nM PykA with 0.5 nM **(D)**, 1 nM **(E)**, and 2 nM **(F)** 625 QD. Average cluster size is given in the plots along with the number of QDs counted. Corresponding bar plots for each sample below showing the distribution of cluster sizes present (red) and number of NPs per cluster size (blue).

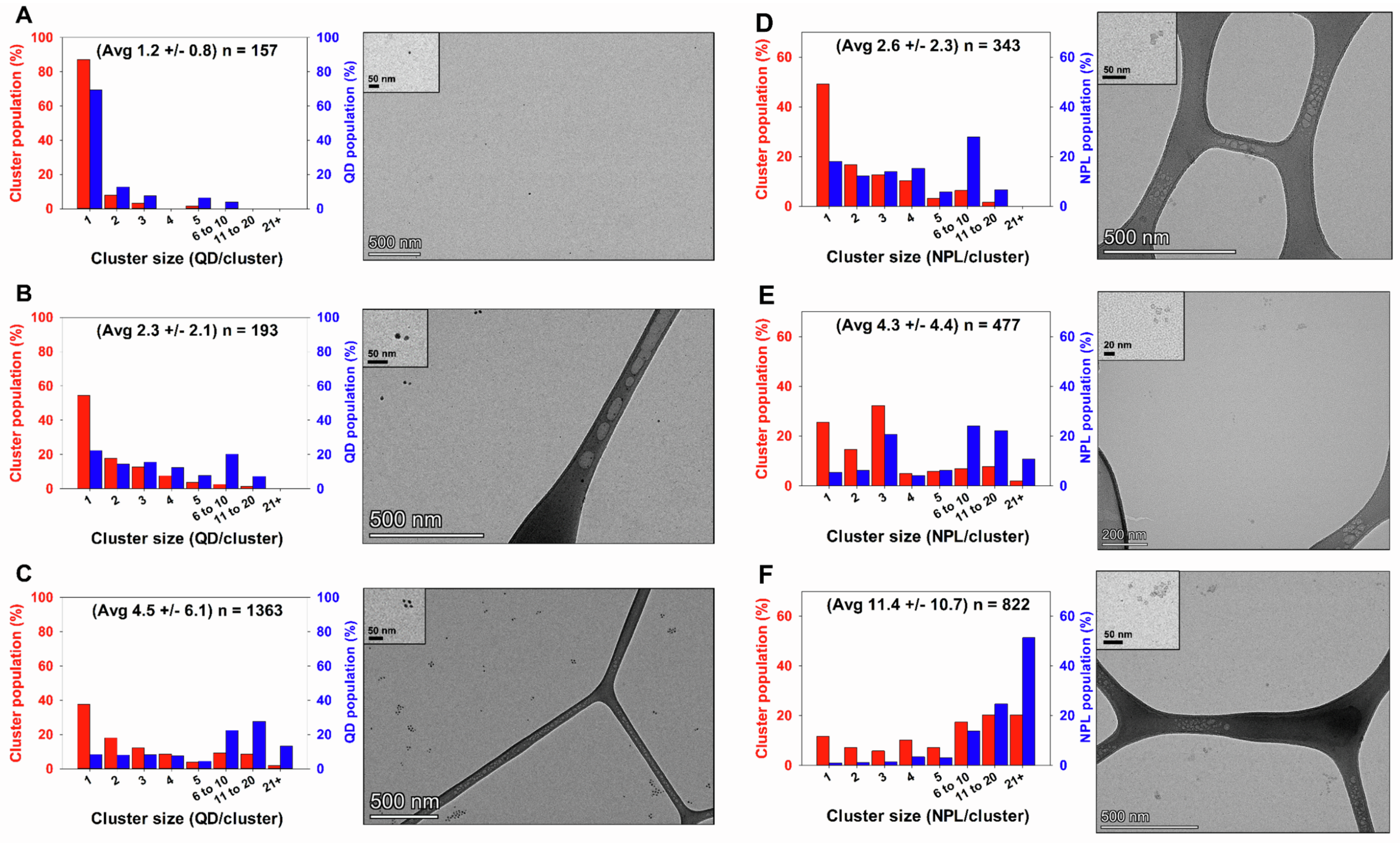

Figure S5. TEM characterization of QD-enzyme clusters with the 641 QDs and NPLs. This data supports the TEM data shown in Figure 1C. Representative TEMs of 641 QDs assembled with 40 nM LDH and 20 nM PykA with 0.5 nM **(A)**, 1 nM **(B)**, and 2 nM **(C)** 641 QD. Average cluster size is given above the micrograph along with the number of QDs counted. Corresponding bar plots for each sample below showing the distribution of cluster sizes present (red) and number of NPs per cluster size (blue). TEM characterization of QD-enzyme clusters with the NPLs. Representative TEMs of NPLs assembled with 40 nM LDH and 20 nM PykA with 0.25 nM **(D)**, 0.5 nM **(E)**, and 1 nM **(F)** NPL. Average cluster size is given in the plots along with the number of NPLs counted. Corresponding bar plots for each sample below showing the distribution of cluster sizes present (red) and number of NPLs per cluster size (blue).

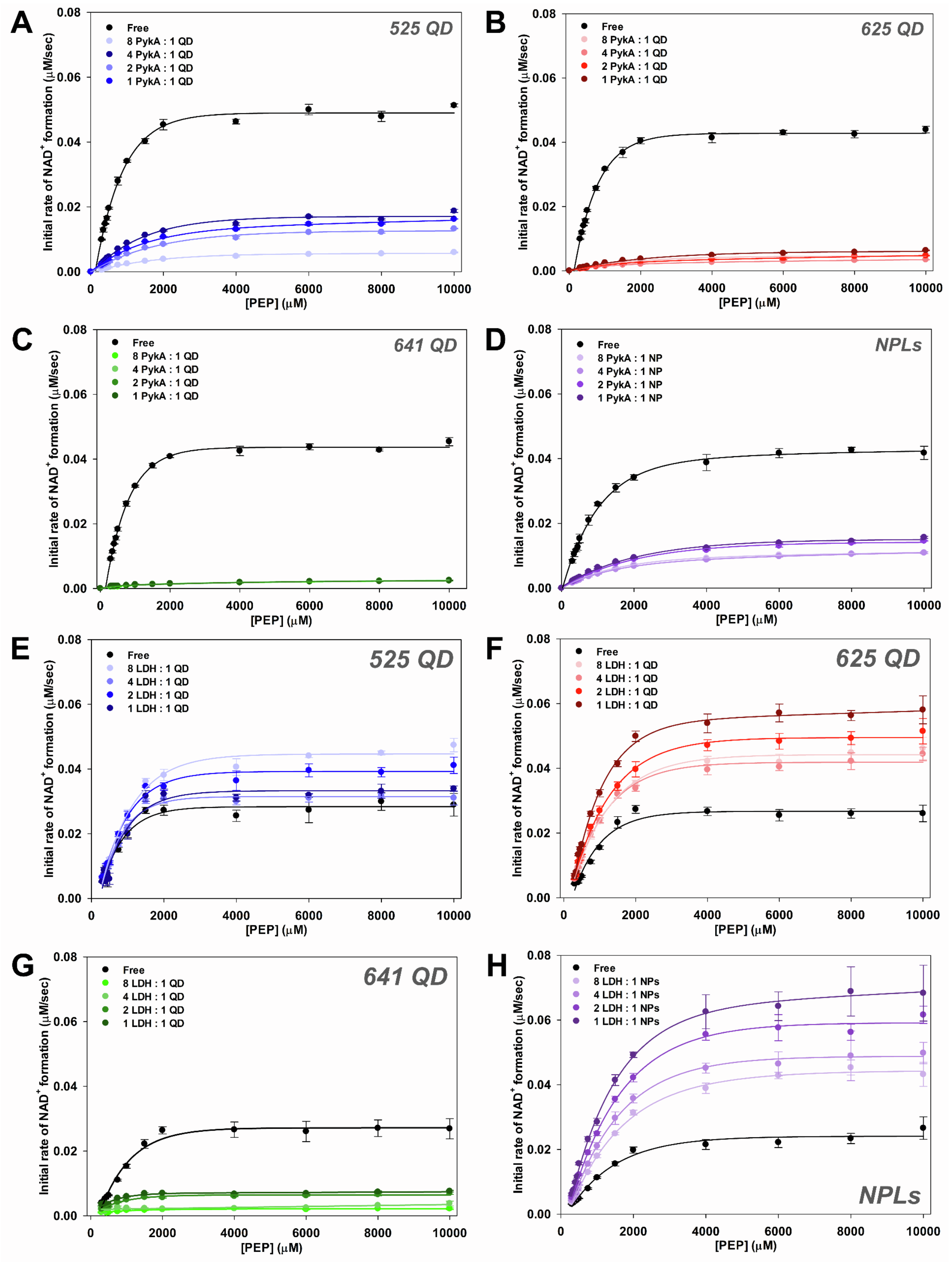
A
525 QD
Free
8 PykA : 1 QD
4 PykA : 1 QD
2 PykA : 1 QD
1 PykA : 1 QD
Initial rate of NAD+ formation (μM/sec)
[PEP] (μM)
B
625 QD
C
641 QD
D
NPLs
8 PykA : 1 NP
4 PykA : 1 NP
2 PykA : 1 NP
1 PykA : 1 NP
E
525 QD
8 LDH : 1 QD
4 LDH : 1 QD
2 LDH : 1 QD
1 LDH : 1 QD
F
625 QD
G
641 QD
H
NPLs
8 LDH : 1 NPs
4 LDH : 1 NPs
2 LDH : 1 NPs
1 LDH : 1 NPs

Figure S6.

Figure S6. MM data for LDH and PykA across the different NP materials. This data supports the kinetic data shown in Figure 3 and Figure 4. (A) MM plots showing initial rates of NAD^+ conversion for free PykA and as assembled to increasing amounts of 525 QD used in the self-assembly *versus* increasing concentrations of PEP. (B) MM plots showing initial rates of NAD^+ conversion for free PykA and as assembled to increasing amounts of 625 QD used in the self-assembly *versus* increasing concentrations of PEP. (C) MM plots showing initial rates of NAD^+ conversion for free PykA and as assembled to increasing amounts of 641 QD used in the self-assembly *versus* increasing concentrations of PEP. (D) MM plots showing initial rates of NAD^+ conversion for free PykA and as assembled to increasing amounts of NPL used in the self-assembly *versus* increasing concentrations of PEP. Reaction conditions for panels A-D include variable PEP, 5 mM $MgCl_2 \times 6H_2O$, 1 mM ADP, 0.25 mM NADH, 2.5 nM PykA, 250 nM LDH, 10 mM KCl, and 0.5 mM EDTA in 120 mM HEPES at pH 8 and 30° C. (E) MM plots showing initial rates of NAD^+ conversion for free LDH and as assembled to increasing amounts of 525 QD used in the self-assembly *versus* increasing concentrations of PEP. (F) MM plots showing initial rates of NAD^+ conversion for free LDH and as assembled to increasing amounts of 625 QD used in the self-assembly *versus* increasing concentrations of PEP. (G) MM plots showing initial rates of NAD^+ conversion for free LDH and as assembled to increasing amounts of 641 QD used in the self-assembly *versus* increasing concentrations of PEP. (H) MM plots showing initial rates of NAD^+ conversion for free LDH and as assembled to increasing amounts of NPL used in the self-assembly *versus* increasing concentrations of PEP. Reaction conditions for panels E-H include variable PEP, 5 mM $MgCl_2 \times 6H_2O$, 1 mM ADP, 0.25 mM NADH, 2.5 nM LDH, 250 nM PykA, 10 mM KCl, and 0.5 mM EDTA in 120 mM HEPES at pH 8 and 30° C.

Table S1. Estimated enzymatic kinetic parameters for PykA when free in solution and as assembled on QDs and NPLs. This data supports the kinetic data shown in Figure 3.

Enzyme: Ratio per QD	V_{Max} (nM × s^{-1})	k_{cat} (sec^{-1})	K_M (mM)	k_{cat} / K_M (mM^{-1} × s^{-1})
**PykA:[a] 0**	63.0 ± 1	25.0 ± 0.1	1.3 ± 0.1	$2.0 \times 10^{-5} \pm 2 \times 10^{-6}$
**525 QDs**				
**1**	17.0 ± 1	6.8 ± 0.1	1.3 ± 0.1	$5.3 \times 10^{-6} \pm 5 \times 10^{-7}$
**2**	14.0 ± 1	5.7 ± 0.1	1.4 ± 0.1	$4.1 \times 10^{-6} \pm 4 \times 10^{-7}$
**4**	21.0 ± 1	8.5 ± 0.1	1.6 ± 0.1	$5.3 \times 10^{-6} \pm 3 \times 10^{-8}$
**8**	6.8 ± 0.2	2.7 ± 0.1	1.7 ± 0.2	$1.6 \times 10^{-6} \pm 2 \times 10^{-7}$
**625 QDs**				
**1**	6.9 ± 0.2	2.7 ± 0.1	1.6 ± 0.1	$1.7 \times 10^{-6} \pm 8 \times 10^{-8}$
**2**	5.4 ± 0.2	2.2 ± 0.1	2.0 ± 0.2	$1.1 \times 10^{-6} \pm 1 \times 10^{-7}$
**4**	3.9 ± 0.1	1.6 ± 0.1	1.8 ± 0.3	$8.8 \times 10^{-7} \pm 2 \times 10^{-7}$
**8**	5.7 ± 0.2	2.3 ± 0.1	1.8 ± 0.2	$1.3 \times 10^{-6} \pm 1 \times 10^{-7}$
**641 QDs**				
**1**	2.7 ± 0.2	1.1 ± 0.1	1.4 ± 0.1	$7.7 \times 10^{-7} \pm 9 \times 10^{-8}$
**2**	2.6 ± 0.1	1.1 ± 0.1	1.5 ± 0.1	$7.0 \times 10^{-7} \pm 2 \times 10^{-8}$
**4**	3.0 ± 0.1	1.2 ± 0.1	1.8 ± 0.2	$6.6 \times 10^{-7} \pm 9 \times 10^{-8}$
**8**	2.6 ± 0.1	1.0 ± 0.1	1.5 ± 0.1	$6.9 \times 10^{-7} \pm 6 \times 10^{-8}$
**NPLs**				
**1**	17.0 ± 1	6.8 ± 0.1	1.5 ± 0.1	$4.5 \times 10^{-6} \pm 4 \times 10^{-7}$
**2**	16.0 ± 1	6.4 ± 0.1	1.5 ± 0.2	$4.2 \times 10^{-6} \pm 5 \times 10^{-7}$
**4**	12.0 ± 1	5.0 ± 0.1	1.7 ± 0.2	$2.9 \times 10^{-6} \pm 4 \times 10^{-7}$
**8**	12.0 ± 1	5.0 ± 0.1	1.5 ± 0.1	$3.2 \times 10^{-6} \pm 2 \times 10^{-7}$

Notes. Final enzyme concentration: [a]PykA = 2.5 nM. All kinetic values are qualified as apparent. Ratio of 0 = free enzyme in solution, no QD present.

Table S2. Estimated enzymatic kinetic parameters for LDH when free in solution and as assembled on QDs and NPLs. This data supports the kinetic data shown in Figure 3.

Enzyme: Ratio per QD	V_{Max} (nM × s^{-1})	k_{cat} (sec^{-1})	K_M (mM)	k_{cat} / K_M (mM^{-1} × s^{-1})
**LDH:[a] 0**	33.0 ± 3	13.3 ± 0.2	0.9 ± 0.4	$1.5 \times 10^{-5} \pm 7 \times 10^{-6}$
**525 QDs**				
**1**	40.0 ± 6	15.9 ± 0.4	1.1 ± 0.2	$1.5 \times 10^{-5} \pm 5 \times 10^{-7}$
**2**	45.0 ± 1	17.9 ± 0.1	0.9 ± 0.4	$2.0 \times 10^{-5} \pm 7 \times 10^{-6}$
**4**	40.0 ± 1	15.5 ± 0.1	1.1 ± 0.3	$1.4 \times 10^{-5} \pm 4 \times 10^{-6}$
**8**	57.0 ± 1	22.7 ± 0.1	1.5 ± 0.4	$1.5 \times 10^{-5} \pm 3 \times 10^{-6}$
**625 QDs**				
**1**	69.0 ± 1	27.6 ± 0.2	1.3 ± 0.3	$2.1 \times 10^{-5} \pm 5 \times 10^{-6}$
**2**	58.0 ± 3	23.1 ± 0.2	1.0 ± 0.2	$2.3 \times 10^{-5} \pm 4 \times 10^{-6}$
**4**	49.0 ± 1	19.5 ± 0.1	1.1 ± 0.3	$1.8 \times 10^{-5} \pm 5 \times 10^{-6}$
**8**	52.0 ± 1	20.7 ± 0.1	1.2 ± 0.3	$1.7 \times 10^{-5} \pm 5 \times 10^{-6}$
**641 QDs**				
**1**	8.3 ± 0.1	3.32 ± 0.1	0.6 ± 0.2	$6.0 \times 10^{-6} \pm 2 \times 10^{-6}$
**2**	7.7 ± 0.3	3.07 ± 0.1	0.8 ± 0.1	$3.8 \times 10^{-6} \pm 7 \times 10^{-7}$
**4**	3.6 ± 0.5	1.45 ± 0.1	0.7 ± 0.3	$2.1 \times 10^{-6} \pm 1 \times 10^{-6}$
**8**	2.6 ± 0.3	1.04 ± 0.1	0.8 ± 0.1	$1.3 \times 10^{-6} \pm 3 \times 10^{-7}$
**NPLs**				
**1**	81.0 ± 9	32.3 ± 0.6	1.6 ± 0.3	$2.0 \times 10^{-5} \pm 6 \times 10^{-6}$
**2**	71.0 ± 4	28.6 ± 0.3	0.2 ± 0.2	$1.7 \times 10^{-5} \pm 2 \times 10^{-6}$
**4**	59.0 ± 5	23.6 ± 0.3	1.7 ± 0.4	$1.4 \times 10^{-5} \pm 4 \times 10^{-6}$
**8**	52.0 ± 2	20.9 ± 0.1	1.7 ± 0.3	$1.2 \times 10^{-5} \pm 3 \times 10^{-6}$

Notes. Final enzyme concentration: [a]LDH = 2.5 nM. All kinetic values are qualified as apparent. Ratio of 0 = free enzyme in solution, no QD present.

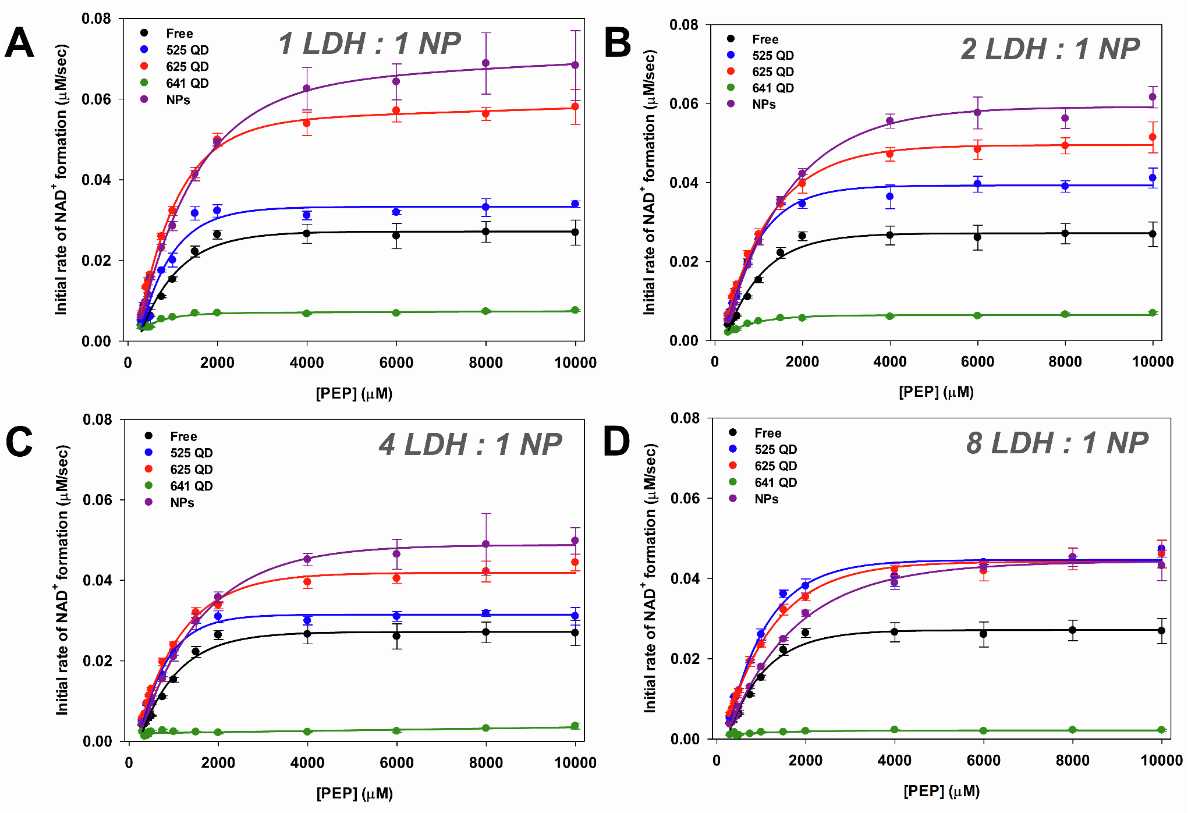

Figure S7. MM plots showing initial rates of NAD^+ conversion for free LDH and as assembled across different nanoparticles used in the self-assembly *versus* increasing concentrations of PEP. This data supports the kinetic data shown in Figure 3. Data collected at the LDH to NPL ratios of 1:1 (A), 2:1 (B), 4:1 (C), and 8:1 (D). Reaction conditions include variable PEP, 5 mM $MgCl_2 \times 6H_2O$, 1 mM ADP, 0.25 mM NADH, 2.5 nM LDH, 250 nM PykA, 10 mM KCl, and 0.5 mM EDTA in 120 mM HEPES at pH 8 and 30° C.

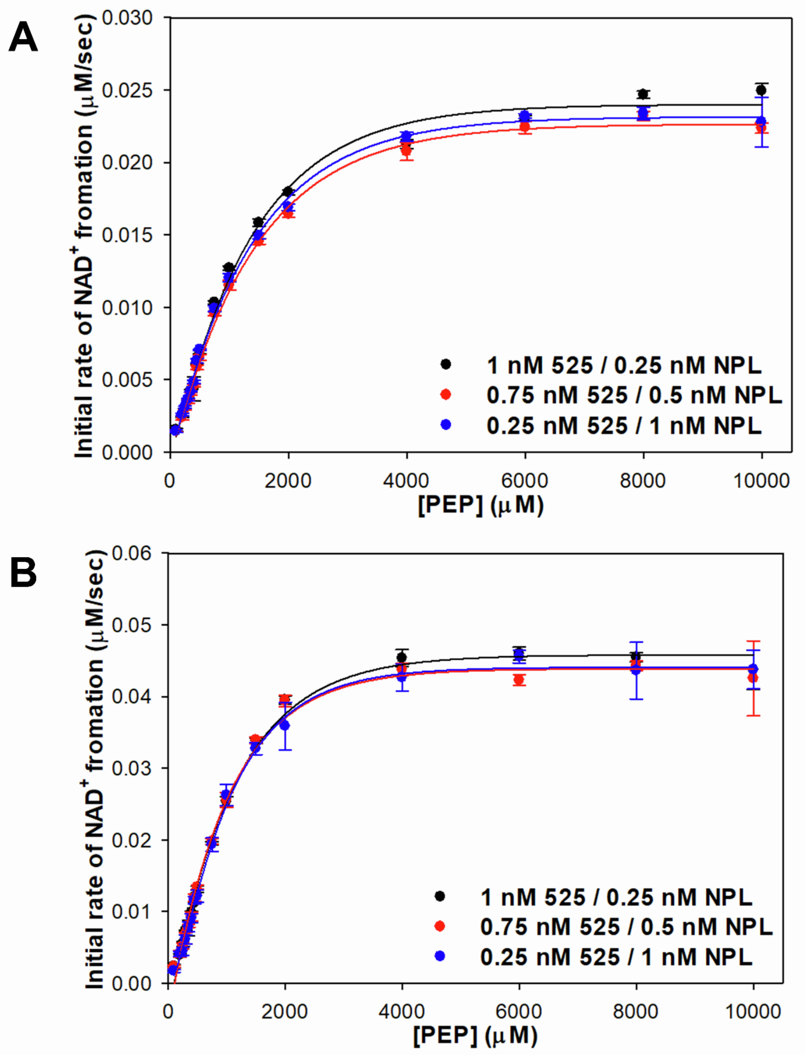

Figure S8. Mixed NP assembly MM data for LDH and PykA. This data supports the kinetic data shown in Figure 5. (A) MM plots showing initial rates of NAD^+ conversion for PykA as assembled across mixed nanoparticles used in the self-assembly *versus* increasing concentrations of PEP. Reaction conditions include variable PEP, 5 mM $MgCl_2 \times 6H_2O$, 1 mM ADP, 0.25 mM NADH, 250 nM LDH, 2.5 nM PykA, 10 mM KCl, and 0.5 mM EDTA in 120 mM HEPES at pH 8 and 30° C. (B) MM plots showing initial rates of NAD^+ conversion for LDH as assembled across mixed nanoparticles used in the self-assembly *versus* increasing concentrations of PEP. Reaction conditions include variable PEP, 5 mM $MgCl_2 \times 6H_2O$, 1 mM ADP, 0.25 mM NADH, 2.5 nM LDH, 250 nM PykA, 10 mM KCl, and 0.5 mM EDTA in 120 mM HEPES at pH 8 and 30° C.

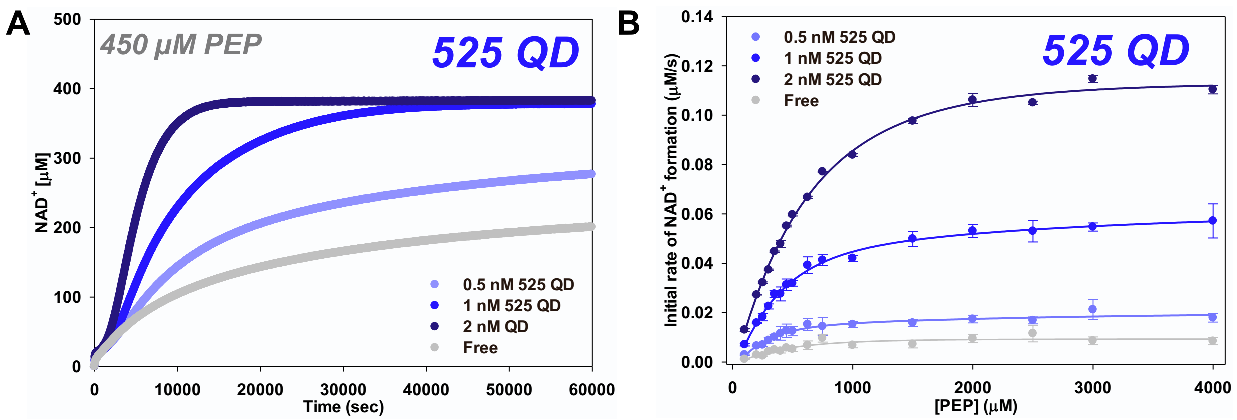

Figure S9. Kinetic enhancement from channeling in the two enzyme cascade clustered with 525 QDs. This data supports the kinetic data shown in Figure 3. (**A**) Traces of NAD^+ concentration versus time for the two-enzyme cascade at increasing concentrations of 525 QD with 450 μM PEP. (**B**) Plots of k_{flux} showing initial rates of NAD^+ conversion for the two-enzyme cascade across increasing amounts of 525 QD used in the self-assembly *versus* increasing concentrations of PEP.

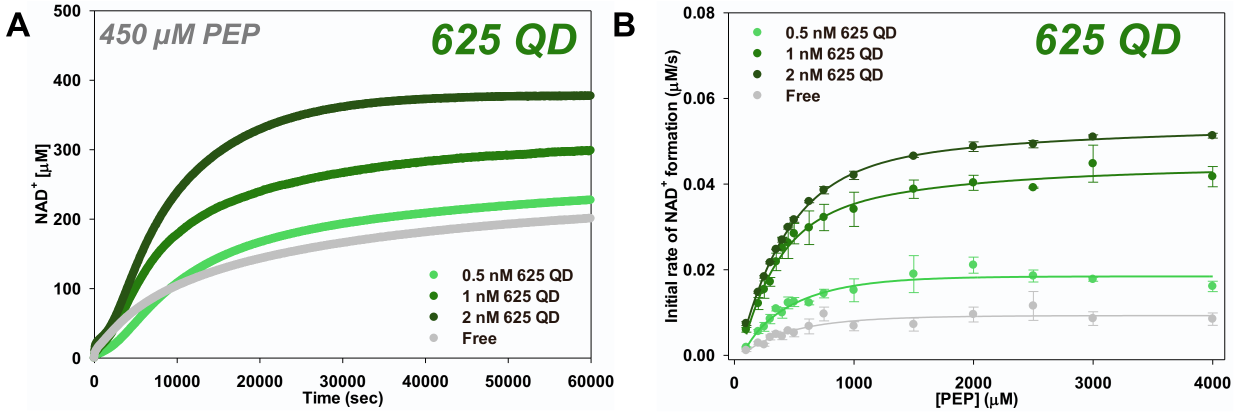

Figure S10. Kinetic enhancement from channeling in the two enzyme cascade clustered with 625 QDs. This data supports the kinetic data shown in Figure 3. (**A**) Traces of NAD^+ concentration versus time for the two-enzyme cascade at increasing concentrations of 625 QD with 450 μM PEP. (**B**) Plots of k_{flux} showing initial rates of NAD^+ conversion for the two-enzyme cascade across increasing amounts of 625 QD used in the self-assembly *versus* increasing concentrations of PEP.

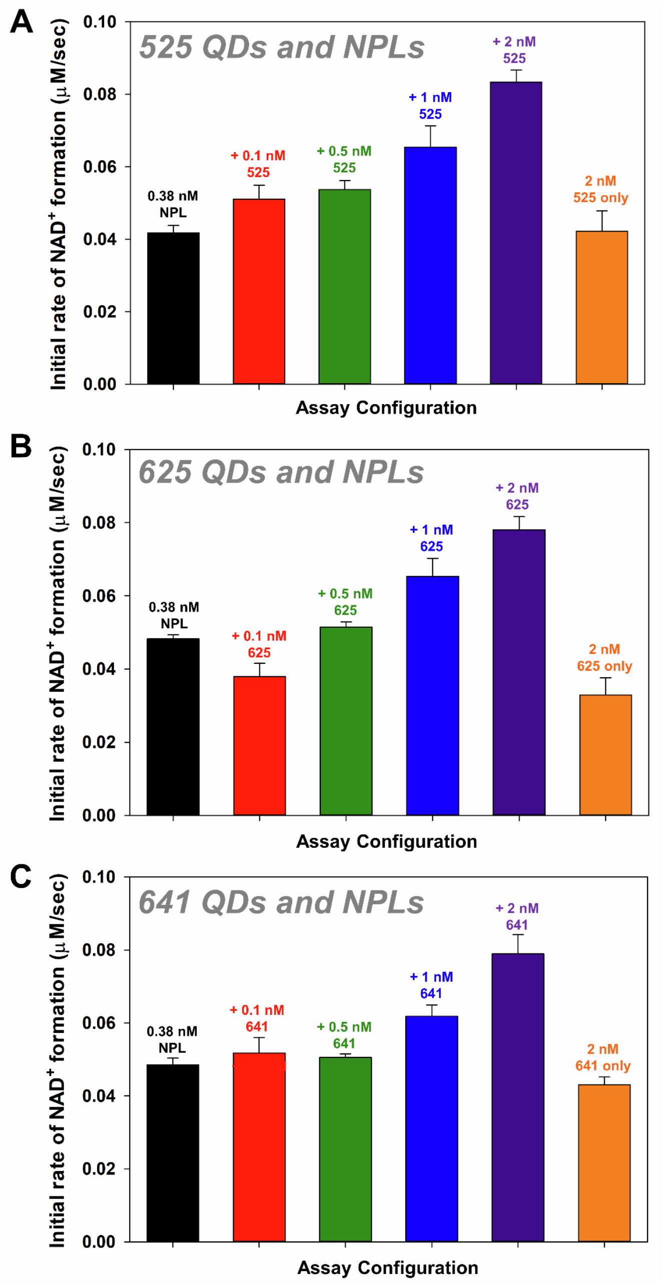

Figure S11. Changes in initial rate at 4000 µM PEP in the two-enzyme cascade as the result of mixed QD-NPL clusters engaged in channeling. This data supports the kinetic data shown in Figure 5. (**A**) Plots of initial rate of NAD^+ conversion for the two-enzyme cascade with 0.375 nM NPL and increasing amounts of 525 QD used in the self-assembly at 4000 µM PEP. (**B**) Plots of initial rate of NAD^+ conversion for the two-enzyme cascade with 0.375 nM NPL and increasing amounts of 625 QD used in the self-assembly at 4000 µM PEP. (**C**) Plots of initial rate of NAD^+ conversion for the two-enzyme cascade with 0.375 nM NPL and increasing amounts of 641 QD used in the self-assembly at 4000 µM PEP. Enzyme concentrations held constant in each assay while nanoparticle concentrations varied. Data points from replicate samples and standard deviations were <15% in all cases.

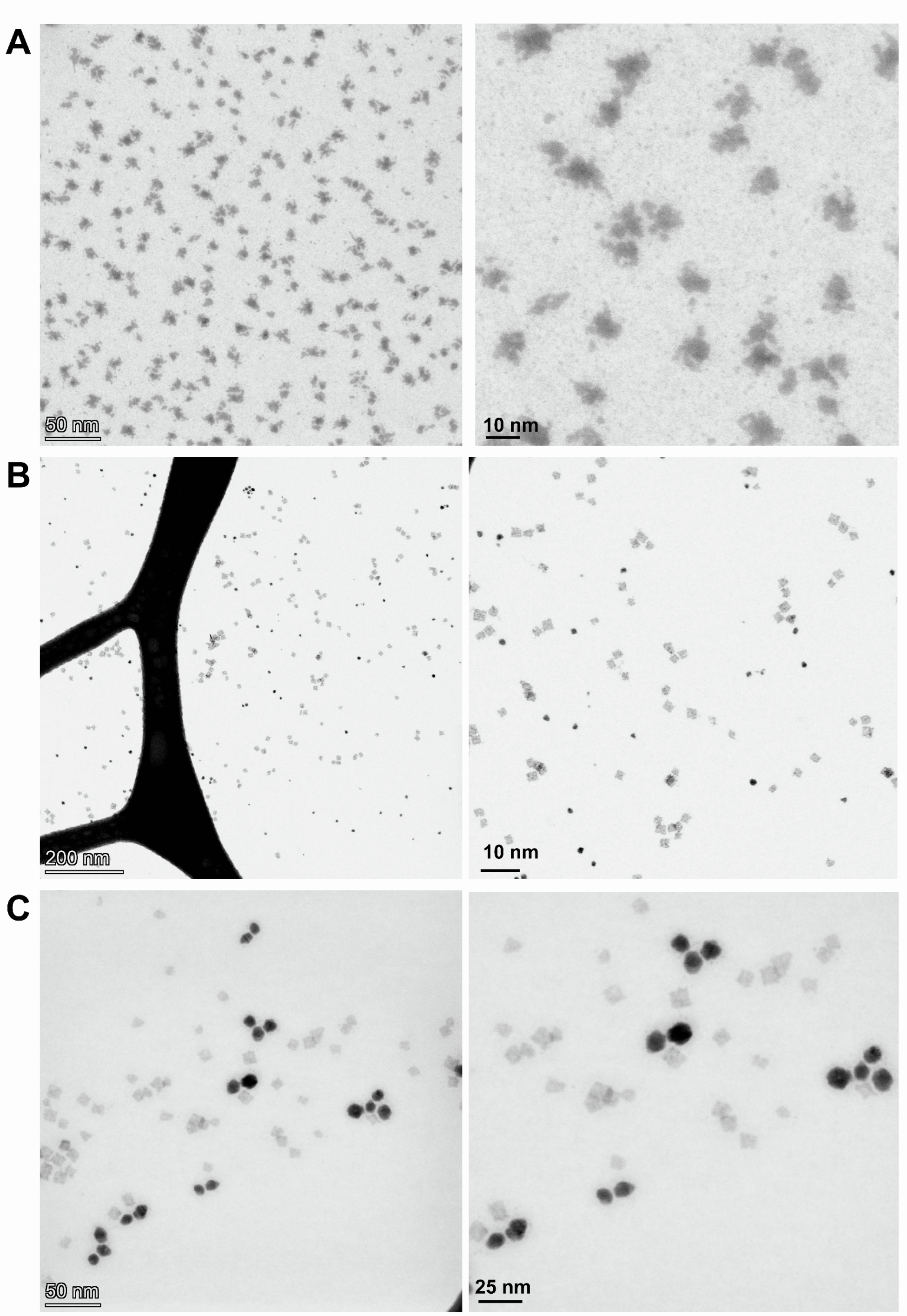

Figure S12. TEM images of mixed NPL systems. This data supports the kinetic data shown in Figure 2B. Samples with NPL and 525 QDs **(A)**, 625 QDs **(B)**, or 641 QDs **(C)**. Conditions: 10 mM HEPES, 0.38 nM NPL, 0.75 nM QD, 40 nM LDH, and 20 nM PyKA.

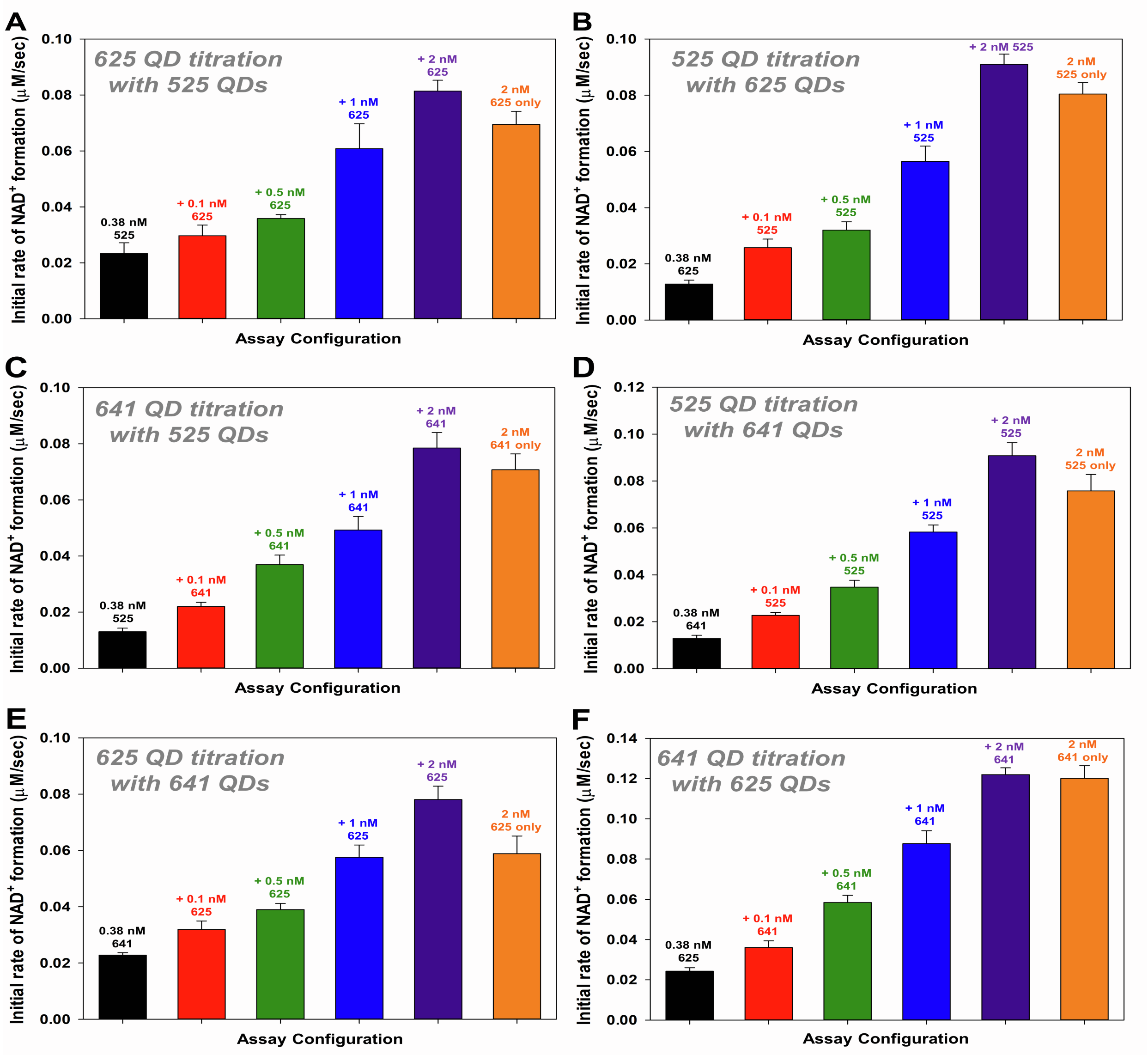

Figure S13. Changes in initial rate at 4000 µM PEP in the two-enzyme cascade as the result of mixed QDs of different sizes. This data supports the kinetic data shown in Figure 4 and Figure 5. (**A**) Plots of initial rate of NAD^+ conversion for the two-enzyme cascade with 0.38 nM 525 QD and increasing amounts of 625 QD used in the self-assembly at 4000 µM PEP. (**B**) Plots of initial rate of NAD^+ conversion for the two-enzyme cascade with 0.38 nM 625 QD and increasing amounts of 525 QD used in the self-assembly at 4000 µM PEP. (**C**) Plots of initial rate of NAD^+ conversion for the two-enzyme cascade with 0.38 nM 525 QD and increasing amounts of 641 QD used in the self-assembly at 4000 µM PEP. (**D**) Plots of initial rate of NAD^+ conversion for the two-enzyme cascade with 0.38 nM 641 QD and increasing amounts of 525 QD used in the self-assembly at 4000 µM PEP. (**E**) Plots of initial rate of NAD^+ conversion for the two-enzyme cascade with 0.38 nM 641 QD and increasing amounts of 625 QD used in the self-assembly at 4000 µM PEP. (**F**) Plots of initial rate of NAD^+ conversion for the two-enzyme cascade with 0.38 nM 625 QD and increasing amounts of 641 QD used in the self-assembly at 4000 µM PEP. Enzyme concentrations held constant in each assay while nanoparticle concentrations varied. Data points from replicate samples and standard deviations were <15% in all cases.

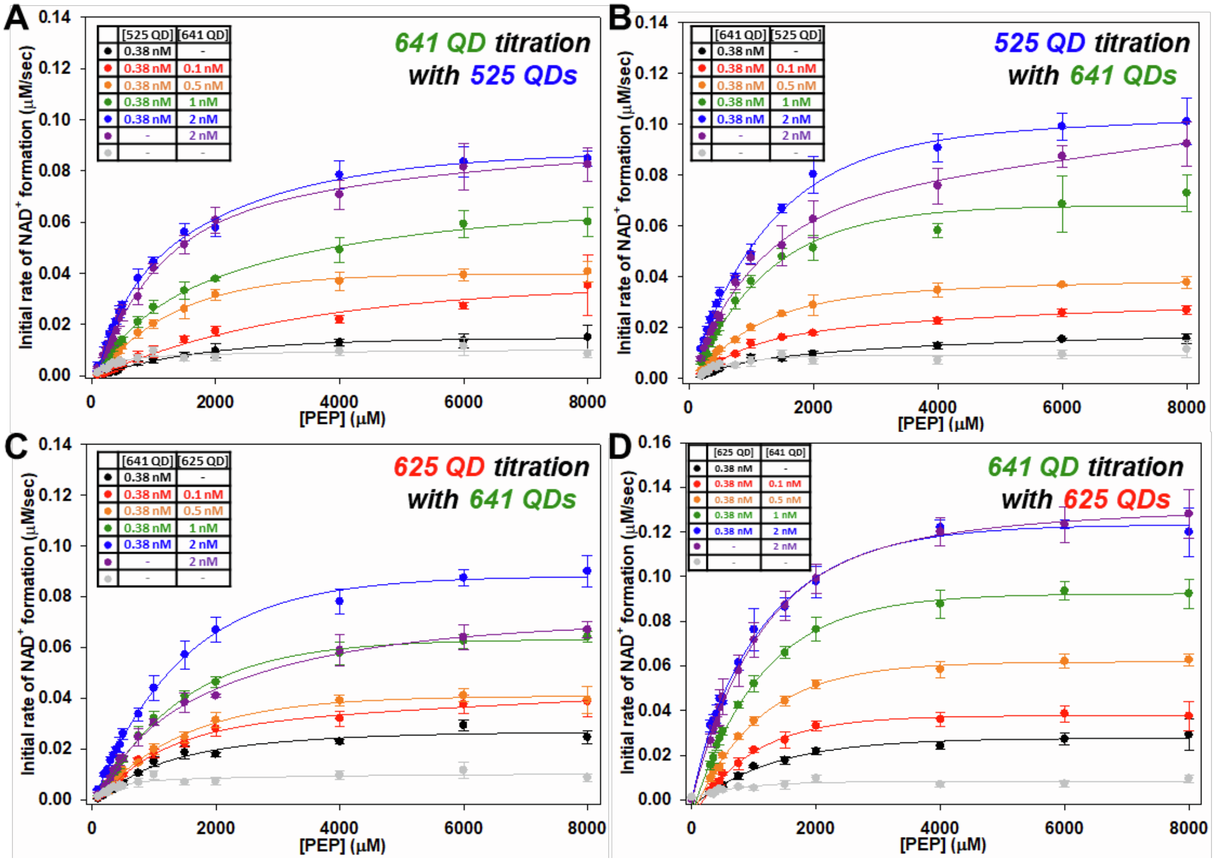

Figure S14. Mixed QD-QD assembly systems. This data supports the kinetic data shown in Figure 5. (A) Plots of k_{flux} showing initial rates of NAD^+ conversion for the two-enzyme cascade with 0.38 nM 525 QD and increasing amounts of 641 QD used in the self-assembly versus increasing concentrations of PEP. **(B)** Plots of k_{flux} showing initial rates of NAD^+ conversion for the two-enzyme cascade with 0.38 nM 641 QD and increasing amounts of 525 QD used in the self-assembly versus increasing concentrations of PEP. **(C)** Plots of k_{flux} showing initial rates of NAD^+ conversion for the two-enzyme cascade with 0.38 nM 641 QD and increasing amounts of 625 QD used in the self-assembly versus increasing concentrations of PEP. **(D)** Plots of k_{flux} showing initial rates of NAD^+ conversion for the two-enzyme cascade with 0.38 nM 625 QD and increasing amounts of 641 QD used in the self-assembly versus increasing concentrations of PEP. Enzyme concentrations held constant in each assay while nanoparticle concentrations varied. Data points from replicate samples and standard deviations were <15% in all cases. Trend lines to aid the eye are included in A-D, these are not necessarily the MM fits.
