# Supplementary material for: Optimizing the conversion of phosphoenolpyruvate to lactate by enzymatic channeling with mixed nanoparticle display
Source: Cell Rep Methods. 2024 May 6;4(5):100764. doi: 10.1016/j.crmeth.2024.100764 (PMC11133815; doi:10.1016/j.crmeth.2024.100764)
Supplement: Document S2. Article plus supplemental information [file mmc2.pdf]

Cell Reports Methods

Article

Optimizing the conversion of phosphoenolpyruvate to lactate by enzymatic channeling with mixed nanoparticle display

Graphical abstract

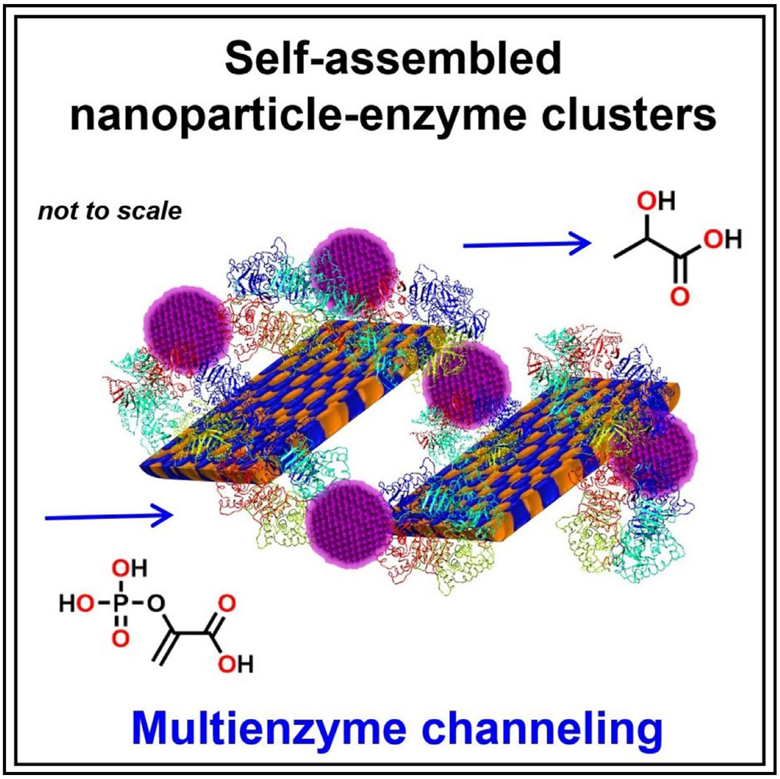

Highlights

- Assembly of enzyme nanoclusters with mixed nanoparticle types increases enzymatic channeling
- Mixing nanoparticle sizes and shapes enables structural optimization of nanoclusters
- Designer cascades with almost any enzyme type should be possible with this approach
- A mixed nanoparticle approach is demonstrated with two- and seven-enzyme systems

Authors

Shelby L. Hooe, Christopher M. Green, Kimihiro Susumu, Michael H. Stewart, Joyce C. Breger, Igor L. Medintz

Correspondence

igor.medintz@nrl.navy.mil

In brief

Enzymatic channeling is a highly efficient form of multienzyme catalysis with much to offer for synthetic biology but remains challenging to implement. Hooe et al. demonstrate an approach of co-assembling enzymes with different nanoparticle sizes and shapes into nanoclusters, resulting in channeling and increasing catalytic flux by orders of magnitude.

Hooe et al., 2024, Cell Reports Methods *4*, 100764
May 20, 2024 Published by Elsevier Inc.
https://doi.org/10.1016/j.crmeth.2024.100764

CellPress

Cell Reports Methods

CellPress
OPEN ACCESS

Article

Optimizing the conversion of phosphoenolpyruvate to lactate by enzymatic channeling with mixed nanoparticle display

Shelby L. Hooe,[1] Christopher M. Green,[1] Kimihiro Susumu,[2] Michael H. Stewart,[2] Joyce C. Breger,[1] and Igor L. Medintz[1,3,*]

[1]Center for Bio/Molecular Science and Engineering Code 6900, U.S. Naval Research Laboratory, Washington, DC 20375, USA
[2]Optical Sciences Division Code 5611, U.S. Naval Research Laboratory, Washington, DC 20375, USA
[3]Lead contact
*Correspondence: igor.medintz@nrl.navy.mil
https://doi.org/10.1016/j.crmeth.2024.100764

MOTIVATION The ability to synthesize complex molecules in a green manner using enzymes represents a sustainable strategy toward replacing many fossil fuel-derived chemical feedstock and energy-intensive synthetic processes. To offer greater synthetic versatility, such synthetic biology approaches must expand beyond the limits of cell-based systems and bypass cellular toxicity to create new molecules that cells cannot make. Thus, methods to join multienzyme cascades together *in vitro* to function in the most efficient manner possible are highly desirable. We show how different nanoparticle materials allow display and clustering of two coupled enzymes into aggregates that access channeling phenomena and increase catalytic flux by orders of magnitude. Mixing different nanoparticles together in this system enhances reactions beyond the capabilities of a single-size nanoparticle material and enables for selection of a nanoparticle-enzyme cluster that has been more structurally optimized for increased product yield with fewer reactants.

SUMMARY

Co-assembling enzymes with nanoparticles (NPs) into nanoclusters allows them to access channeling, a highly efficient form of multienzyme catalysis. Using pyruvate kinase (PykA) and lactate dehydrogenase (LDH) to convert phosphoenolpyruvic acid to lactic acid with semiconductor quantum dots (QDs) confirms how enzyme cluster formation dictates the rate of coupled catalytic flux (k_{flux}) across a series of differentially sized/shaped QDs and 2D nanoplatelets (NPLs). Enzyme kinetics and coupled flux were used to demonstrate that by mixing different NP systems into clusters, a >10× improvement in k_{flux} is observed relative to free enzymes, which is also ≥2× greater than enhancement on individual NPs. Cluster formation was characterized with gel electrophoresis and transmission electron microscopy (TEM) imaging. The generalizability of this mixed-NP approach to improving flux is confirmed by application to a seven-enzyme system. This represents a powerful approach for accessing channeling with almost any choice of enzymes constituting a multienzyme cascade.

INTRODUCTION

The ability to synthesize complex molecules via green, sustainable strategies continues to drive research interest in synthetic biology (SynBio). This is due to the potential that SynBio has to enable a circular bioeconomy capable of addressing numerous socioeconomic issues surrounding environmental challenges and increased use of fossil fuel-derived chemical feedstocks along with the energy requirements of industrial chemical synthesis.[1–5] SynBio looks to renewable bulk feedstocks derived from agriculture or waste, which are then converted into industrial chemical intermediaries and fine product molecules by enzymatic processes. Moreover, it can be implemented in a distributed manner matching available resources in contrast to the centralized localization of refineries next to naval shipping hubs.[3,4,6–8] Cell-based SynBio is the most common approach and represents a cost-efficient route to synthesize desired molecules on an industrial scale because of the ability to use large fermenters filled with self-replicating, biofactories.[9] However, within cell-based systems, the full synthetic potential of a given enzyme and/or multienzyme cascade cannot be realized in many cases. This is because within an enclosed cellular system

This is an open access article under the CC BY license (http://creativecommons.org/licenses/by/4.0/).

the efficiency of the enzyme(s) is limited by many competing pathways as cells are evolutionarily optimized to minimize metabolic redundancy.[10] Target molecule production also cannot exceed that of the cells tolerance and resistance to intermediary/product toxicity. Pertinently, living cells have a very limited chemical space in which they operate and generally do not tolerate the vast majority of non-natural molecules as substrates. Paradoxically, individual enzymes tolerate many non-natural substrates while it is other coupled metabolic pathways or end-products that manifest the cellular toxicity. An alternative approach to overcome the limits imposed by cellular toxicity is the application of minimalist cell-free SynBio where necessary pathways are reconstituted outside the cell.

In its minimalist format, this type of cell-free biosynthesis needs only the enzyme(s), substrate(s), cofactor(s), and buffer required for the formation of a desired product.[2,11,12] Minimalist cell-free SynBio is not only an appealing approach when attempting to incorporate non-natural or xenobiotic substrates, it is also an advantageous strategy for optimizing a single target pathway where concentrations/ratios of requisite enzyme(s), substrate(s), and cofactor(s) can be controlled to enhance desired product formation.[10] One apparent challenge associated with such minimalist approaches is the current lack of access toward the enhanced *in vitro* multi-step catalysis that nature provides within cellular systems, whereby the confines of the cell facilitate efficient catalysis by localizing enzymes and negating native substrate/intermediate diffusion through the cellular membrane into the surrounding bulk.[13] Research suggests that within cells, the enzymes constituting a multi-step cascade may form dense clusters of associated enzymes or metabolons via transient interactions to facilitate channeling, the most efficient form of multienzyme catalysis.[14–16] Channeling phenomena arise in multi-step biocatalysis when at least two enzymes are physically held in close proximity to one another such that the intermediary formed by one enzyme reaches the proceeding enzyme in the pathway faster than it can diffuse away into bulk solution. As a nanoscale phenomenon, channeling is observable under diffusion-limited reaction conditions when the "effective" multienzyme catalytic rate $\gg$ diffusion rate of intermediary away from the enzyme.[17–19] The ability to bypass diffusional loss of the intermediate substrate in multienzyme catalysis frequently results in significant increases in overall catalytic flux (k_{flux}) and product formation while requiring less time and fewer reactants.[20,21] Tryptophan synthase is considered the archetype for channeling as its structure contains a hydrophobic barrel, which connecting its α and β catalytic subunits, allowing the indole reaction intermediary to move efficiently between them.[22] In contrast to this almost perfect channeling example, metabolons achieve channeling by proximity or probabilistic processes due to their high-localized density of enzymes, which significantly increases the probability of intermediary finding downstream enzyme.[14–16,23]

Providing multienzyme cascades access to channeling is not a trivial undertaking and even fusion of two coupled enzymes directly together is not a guarantee of success. Keasling had to engineer a 3-dimensional protein scaffold to host three sequential enzymes from the mevalonate pathway at differing stoichiometry to increase product titer 77-fold from acetyl-CoA substrate in *E. coli*.[24] Minteer increased coupled flux between alcohol and aldehyde dehydrogenase almost 500-fold by fusing them together; however, this required considerable *in silico* modeling as part of a rational design beforehand along with mutational protein engineering to optimize the intermediary pathway between the enzymes.[25] Given these issues, many groups have turned to molecular scaffolding to achieve channeling by bringing enzymes into close proximity with each other. Nanomaterials, including DNA, metal organic frameworks, and other inorganic nanomaterials and nanoparticles (NPs) along with protein scaffolds such as virions have been prototyped and tested for these purposes.[20,26–29] Although increased stability and enhancements in an individual enzyme's kinetic profile have been noted following attachment to such scaffolds, and especially to that of DNA,[30,31] there continues to be debate about whether true channeling has been achieved in these systems.[18] Questions remain whether the requisite density of enzymes were properly achieved for channeling or whether other factors such as enzymatic enhancement, localized substrate sequestration, and effects of different (heterogeneous) immobilization chemistries contributed to observed increases in catalytic flux.[32–34]

We have relied on an alternative NP-based scaffolding approach that mimics the function of naturally occurring metabolons to provide enzymes access to probabilistic channeling. We have shown that for minimalist cell-free biosynthesis, utilizing either gold nanoparticles (AuNPs) or semiconductor quantum dots (QDs) as an NP-based enzyme immobilization strategy represents a robust approach to access channeling and/or enhanced enzyme activity.[20,35,36] Here, the enzymes are all expressed with terminal hexahistidine (His_6) motifs for purification by metal-affinity chromatography over Ni^{2+} nitrilotriacetic acid (NTA) media. These same His_6-motifs coordinate to the Zn surface of ZnS-overcoated QDs and NTA displaying AuNPs with high affinity. Moreover, as many of the enzymes are multimeric (e.g., dimers, tetramers), they crosslink the NPs into dense NP-enzyme clusters that manifest channeled catalytic flux. For example, we utilized a two-enzyme cascade consisting of benzaldehyde lyase and alcohol dehydrogenase to convert benzaldehyde and acetaldehyde to (1*R*,2*R*)-1-phenylpropane-1,2-diol, and derivatives thereof, where, upon QD immobilization, the enzymes were capable of engaging in intermediary channeling despite a 10,000-fold difference in their individual catalytic rates.[37] QD immobilization of pyruvate kinase (PykA) and lactate dehydrogenase (LDH) for the conversion of phosphoenolpyruvic acid to lactic acid resulted in a 100-fold improvement in product formation due to channeling.[38] Extensive support for channeling presence in this system included classical experimental formats where reactions were compared with those without QDs present at the same concentrations, underwent shaking to disrupt channeling, separating each enzyme into its own QD assembly, and undertaking detailed numerical simulations of the kinetic process that incorporated a channeling mechanism. Notably, the quaternary structure of the LDH enzyme was significantly stabilized by QD immobilization resulting in improved activity at lower enzyme concentrations.[38] We extended NP channeling to a 10-enzyme system exploiting enzymes from oxidative glycolysis that converted glucose into lactate as self-assembled with QDs

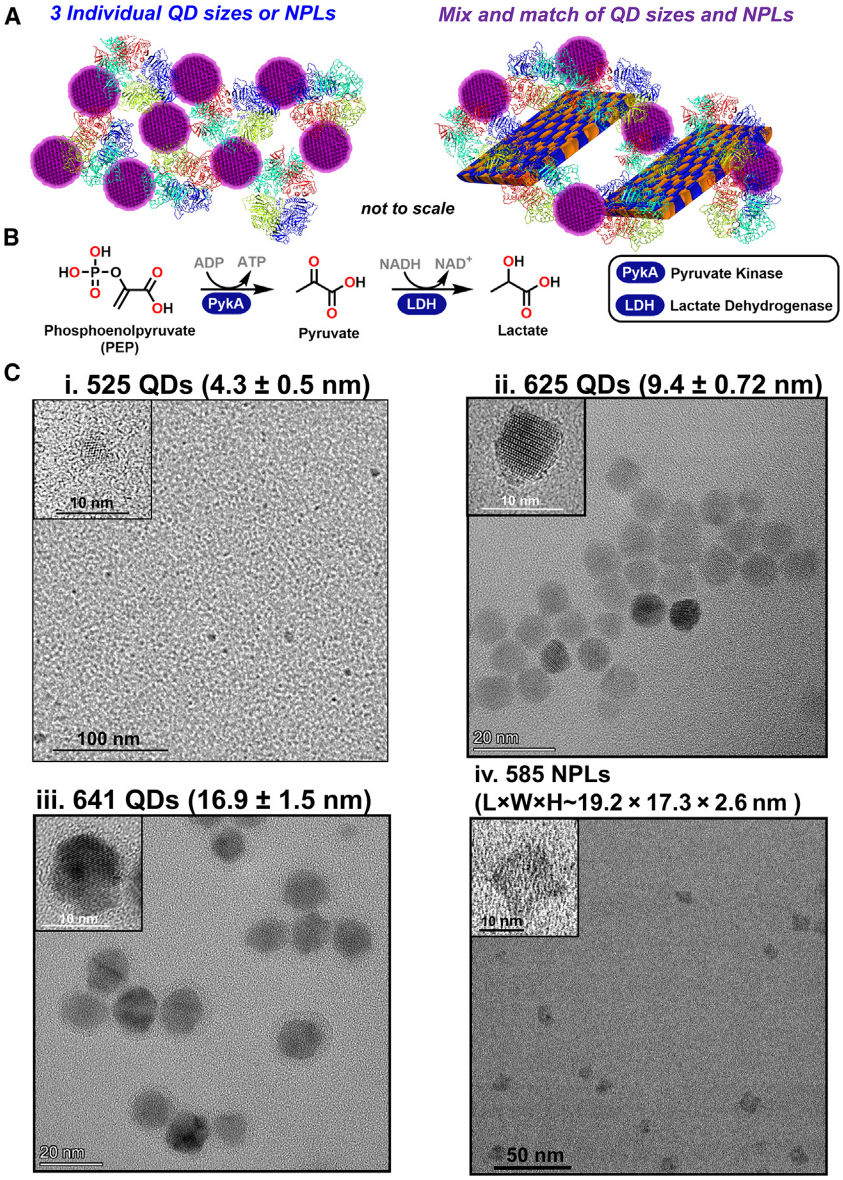

Figure 1. Two-enzyme channeled system converting phosphoenolpyruvate to lactate

(A) Strategies utilized to access and improve intermediary channeling.

(B) Enzyme pathway converting phosphoenolpyruvic acid to lactic acid, → indicates enzymatically catalyzed step(s). Chemical structures of substrate, intermediaries, and final product.

(C) Representative TEMs of (i) 525, (ii) 625, (iii) 641 nm emitting CdSe/CdS/ZnS core/shell/shell QDs, and (iv) NPLs. Inset, high-resolution image of each. Average diameter of each material given in the parenthesis. Note the well-dispersed and non-aggregated QD appearance in the absence of enzyme. The small size of the 525 nm QDs approaches the TEM limit of resolution.

into nanoclusters to access channeled catalytic flux.[20] Replacing spherical QDs with rectangular 2D planar nanoplatelets (NPLs) increased the resulting cluster size and further improved the rate of channeled flux (k_{flux}) significantly. Extensive experimental support including determining the relative transient times all provided strong evidence of a channeling mechanism being again responsible.

Herein, we provide an in-depth analysis of the two-enzyme PykA and LDH cascade where we focus on utilizing QDs of varying size, NPLs, and mixed QD-NPL systems to identify conditions, which enable even further enhancement of product formation via optimization of the underlying cluster architecture (Figures 1A and 1B). Previous work has attempted to correlate nanomaterial size or curvature to activity,[20,36] but to date no study exists where mixed-NP scaffolds have been systematically analyzed to optimize product formation. We demonstrate that by mixing different NP materials a >10× improvement in k_{flux} is observed relative to free enzyme for this bienzymatic system, which is also ≥2× greater than that achieved with any individual NPs. We characterize NP-enzyme assembly in these systems along with utilizing relative NP concentrations as a mechanism to control overall flux. The generalization of this mixed-NP approach to improving flux in the channeled nanoclusters is confirmed by application to a more complex seven-enzyme system.

RESULTS

Enzymes, nanoparticles, self-assembly, and characterization of the nanoparticle-enzyme clusters

For a detailed description of the materials and experimental techniques, see the supplemental information and STAR methods. The first enzyme in the coupled cascade is PykA (EC 2.7.1.40), which converts phosphoenolpyruvate (PEP) to pyruvic acid using adenosine diphosphate (ADP) as the phosphate acceptor to form adenosine triphosphate (ATP). The PykA gene encodes an ~53.5 kDa monomer, which assembles into the active ~220 kDa homotetramer. The second enzyme is LDH (EC 1.1.1.28), which converts pyruvic acid to lactic acid using nicotinamide adenine dinucleotide (NADH) as the reducing cofactor. (Figure 1B). The LDH gene encodes an ~39.1 kDa monomer, which assembles into the active ~160 kDa homotetramer. Both enzymes were cloned directly from *E. coli* strain BL21(DE3) and expressed with an N-terminal $(His)_6$ tag on their respective monomers.[38] Coupled PykA-LDH activity functions in downstream glycolysis as part of glucose metabolism to energy by regenerating ATP. This system was utilized for the current study due to our extensive experience with it on its own and within the context of other extended cascades.[20,38]

For our prototypical NP set, we utilize spherical ~525 nm emitting (average diameter ~4.3 ± 0.5 nm), 625 nm emitting (average

diameter ~9.4 ± 0.7 nm), and 641 nm emitting (average diameter ~16.9 ± 1.5 nm) CdSe/CdS/ZnS core/shell/shell QDs.[20,39] We also utilized ~585 nm emitting CdSe/ZnS core/shell NPLs (four monolayers CdSe) with an average L × W × H of ~19.2 × 17.3 × 2.6 nm, see Figure 1C. NPLs are quasi 2-dimensional (2D) QD-like materials that have been recently described.[40,41] We use NP or nanomaterial as interchangeable descriptors for all materials while QDs and NPLs are used to specify a given type. All NPs were surface functionalized with the zwitterionic dihydrolipoic acid derivative compact ligand 4 (CL4), which replaces the hydrophobic ligands utilized during nanomaterial crystal growth, providing colloidal stability in aqueous buffers, see Figure S1.[42] For enzymatic bioconjugation to NPs, we rely exclusively on self-assembly driven by metal-affinity coordination of the enzyme's pendant $(His)_6$-motifs to the QD's ZnS shell. This cooperative, high-affinity, interaction (K_d ~1 nM) occurs almost spontaneously and follows a Poisson distribution mechanism with the upper packing limit of a monomeric protein around an NP dictated by that protein's geometric fitting constraints based on size and shape.[43,44] $(His)_6$ binds at available ZnS sites on the NP's surface and does not displace the already coordinated CL4 ligands.

Given the homotetrameric structure of PykA and LDH, each enzyme displays multiple pendant $(His)_6$ tags, which will function to crosslink with the QDs and/or NPLs forming nanoclustered or nanoaggregated structures. These are the critical structures that provide the coupled enzymatic systems with the necessary localized density to engage in channeled catalytic flux. It is important to understand this process and the nature of the clusters that are formed to appreciate how it subsequently influences the ability of that system to access probabilistic enzyme channeling. When QDs or NPLs are mixed with multimeric enzymes, they self-assemble and form nanoclusters following a diffusion-limited aggregation mechanism (DLA).[20,37,38] Classical DLA occurs when particles diffusing due to Brownian motion follow a random walk path and then cluster together forming aggregates as they interact.[45] In the current scenario, the DLA process is mechanistically the same but there are now two participants, with each displaying one component of the necessary binding interaction—the $(His)_6$ tag or the QD's receptive ZnS surface. The number of variables involved including NP size/shape, the number of enzymes present, the enzyme's size/shape, reaction volume/concentrations, ratios of protein to NP, etc., means that this process cannot be accurately simulated. Switching to different enzymes or adding more upstream/downstream enzymes all increase the resulting complexity without even considering use of mixed-NP systems. Moreover, the nanoaggregates that form will in actuality be an ensemble of different sizes with each having a different number of component NPs and enzymes present. Nevertheless, despite forming in this non-deterministic manner and as detailed previously, DLA gives rise to clusters with a high density of enzymes in close proximity to each other such that they can engage in channeling.[20,37,38,46] Some control of nanocluster size can be afforded by the relative ratio of NP to overall enzyme present with increased NP presence giving rise to larger clusters. Larger clusters also in general manifest a high level of channeled catalytic flux since more enzymes are present in each cluster in close proximity to each other. Higher protein concentration over NP means smaller clusters as proteins now surround individual NPs with less chance of crosslinking. Higher NP concentration vs. protein means larger clusters as the proteins more readily bind between the NPs and crosslink them.[20] Thus, control over relative ratio of NP to enzyme represents a rudimentary control knob over the rate of channeled flux that can be attained in the clusters.

Agarose gel mobility shift assays were used to confirm individual enzyme and joint bienzyme cascade assembly with either the QD series or the NPLs in a manner similar to that described previously (Figures S2 and S3).[37,38,47] Figure S2 (top) shows representative gel images where 525 QDs (left) and NPLs were assembled with PykA and LDH both individually and together and then imaged as they were subsequently separated in an agarose gel under an electrical field. Increasing enzyme assembly to the NPs will decrease the migration rate in a manner that is somewhat proportional to the underlying ratios. The degree of NP mobility shift is distinctly different when each enzyme is present individually, and as a cascaded assembly, confirming cluster formation under both conditions. Similarly, Figure S2 (bottom) shows analogous representative gel images but with 625 (left) and 641 (right) QDs. Notably, the 641 QDs showed limited mobility in the presence of the enzyme likely due to the larger size of the clusters that formed. Similar mobility shifts during agarose gel separations were also used to confirm the formation of mixed QD-NPL assemblies with the enzymes. Figure 2A shows representative results where different concentrations of 525 QDs (green) and a fixed concentration of 585 NPLs (orange) were assembled with the indicated ratios of PykA and LDH (given as the ratio per NPL). The top image shows the samples as preassembled in Eppendorf tubes where the resulting color is indicative of the amount of QD (green) or NPL (orange) present. The bottom gel images show the samples as separated in a 0.85% agarose gel at different time periods. Mixed QD-NPL samples separate in the gel with a very different migration rate than either alone and this rate changes as the ratio of 525 QD to 585 NPL changes again confirming assembly of the constructs at different ratios (see also Figure S3).

Transmission electron microscopy (TEM) was further utilized to image and semi-quantitatively characterize the relative size of the clusters that formed and how these are subsequently altered as the ratio of NP to enzyme is increased. We define clusters as NPs that appear to be coassembled together with a separation distance less than or equal to the size of the NP itself. Clusters are binned or defined by the number of NPs present in each. Figures S4 and S5 show representative TEM micrographs of the 525, 625, and 641 QDs as well as the NPLs assembled with a fixed concentration of 40 nM LDH and 20 nM PykA as the QD concentration was increased from 0.5 to 1 and then 2 nM while the NPL concentration utilized was half that. Data from these micrographs are plotted as a function of cluster size with the percent of the total population in a given cluster size shown in red and the population of QD or NPL in that cluster given in blue. Notably, a change in the cluster distribution can be observed across increasing concentrations of all four NPs, where the concentration of LDH and PykA remained constant reflecting the formation of larger clusters when the concentration of NP was increased relative to enzyme present. Across

CellPress
OPEN ACCESS

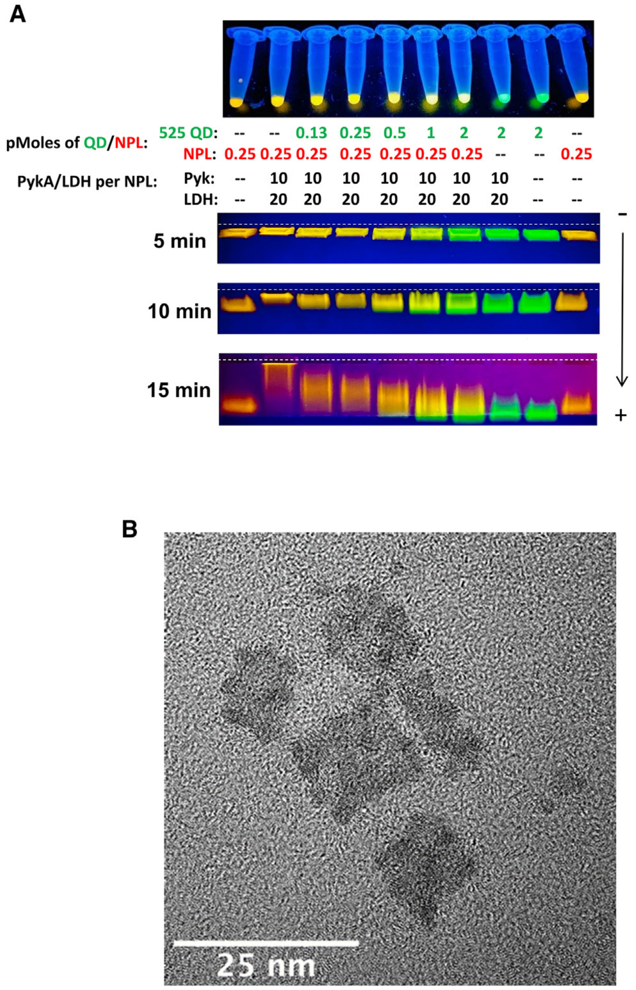

Figure 2. Mixed nanoparticle-enzyme clusters formed with QDs and NPLs

(A) Top: 525-nm emitting QDs (green) and 585-nm emitting NPLs (orange) assembled with indicated ratios of PykA, LDH, and 525 QDs relative to fixed NPL concentration. Samples shown in the Eppendorf tubes were assembled with the resulting photoluminescent color indicative of the amount of QD or NPL present. Bottom: Samples separated in a 0.85% agarose gel run in 1× TBE buffer. Samples separated using ~10 V per cm gel length and image collected every 5 min using a cellphone camera. White dashed line indicates location of the wells. Fluorescent images collected on a UV-trans-illuminator with 365 nm excitation.

(B) Representative TEM micrograph from a sample mixture containing nanoclusters formed from LDH (40 nM), PykA (20 nM), 525 QDs (1 nM), and NPLs (0.38 nM) present.

increasing concentrations of the 525 QDs, the average cluster size increased from 1.4 ± 0.6 at 0.5 nM 525 QD to 4.9 ± 4.3 at 2 nM 525 QD (Figures S4A–S4C). Similarly, across increasing concentrations of the 641 QDs (Figures S5A–S5C), the average cluster size increased from 1.2 ± 0.8 at 0.5 nM 641 QD to 4.5 ± 6.1 at 2 nM 641 QD. The 625 QDs produced an assembly pattern similar to the 641 QDs (Figures S4D–S4F). Interestingly, across increasing concentrations of NPLs, the average cluster size increased from 2.6 ± 2.3 at 0.25 nM NPL to 11.4 ± 10.7 at 1 nM NPL (Figures S5D–S5F). These results suggest that while the cluster distribution remains similar across the different QD sizes, the use of the NPLs enable larger sized clusters to form at lower concentrations than that used for the QDs. These data are similar to those previously seen with the NPLs when they were assembled with a seven-enzyme cascade drawn from oxidative glycolysis.[20] Last, we obtained TEM data to confirm mixed-NP cluster formation from a mixture containing LDH (40 nM) and PykA (20 nM) with both the 525 QDs (1 nM) and NPLs (0.38 nM) present. As shown in Figure 2B, successful formation of mixed-NP clusters was confirmed. These types of mixed-NP systems were not quantified by analyzing cluster distributions due to the complexity of the resulting clusters. However, these data do still confirm that clusters containing both 525 QDs and NPLs successfully form during self-assembly of this two-enzyme cascade.

Kinetic profile of PykA and LDH when free and as NP assembled

The kinetic profiles for PykA and LDH, both free in solution and when NP-displayed (on-QD or on-NPL) at different ratios, were next characterized. Michaelis–Menten (MM) assay formats using excess substrate ([S] $\gg >$ [E]) to meet standard Briggs-Haldane expectations were applied in the same manner as previously described.[17,38] While the active QD-enzyme clusters do not meet all the strictest definitions of the MM formalism, values derived from this analysis nevertheless provide a useful basis for comparison between free enzyme performance and on-NP assays; however, all reported values are qualified as "apparent." Tables 1, S1, and S2 list the MM descriptors for PykA and LDH, respectively, including the maximal velocity (V_{max}), catalytic rate (k_{cat}), Michaelis constant (K_M), and the k_{cat}/K_M ratio, which is a second-order rate constant giving the kinetic efficiency—sometimes referred to as the specificity constant.[17] Assays were carried out with equal concentrations of each enzyme free in solution and then as assembled with ratios of 1, 2, 4, and 8 enzymes/NP. The assays monitored changes to NAD^+ formation via absorbance in a microtiter plate reader either directly from the enzyme in question or in a coupled enzyme format.[17,20]

As seen in Table S1 and the representative progress curves shown in Figures S6A–S6D, for PykA the catalytic rate (k_{cat} = 25 s^{-1}) and efficiency (k_{cat}/K_M = 2.0 × 10^{-5} $mM^{-1}s^{-1}$) decreased by ~70% upon NP immobilization. However, immobilization was shown to increase the activity of LDH as compared with the freely diffusing enzyme (Figures S6E–S6H; S7). For free LDH, the catalytic rate (k_{cat} = 13.3 s^{-1}) and efficiency (k_{cat}/K_M = 1.5 × 10^{-5} $mM^{-1}s^{-1}$) were comparable to those of free PykA (Table S2). Upon LDH immobilization onto the 525 QDs, k_{cat} increased to 17.9 s^{-1} from 13.3 s^{-1} (~35%) at the 2 LDH to 1 QD ratio and to 22.7 s^{-1} (~72%) at a ratio of 8 LDH. Similarly, upon LDH immobilization onto the 625 QDs, k_{cat} increased to 27.6 s^{-1} (~110%) and 23.1 s^{-1} (76%) at QD display ratios of 1 and 2, respectively. However, upon LDH immobilization onto the 641 QDs, k_{cat} decreased to 3.1 s^{-1} (−86%) at the 2 LDH to 1 QD ratio or worse for other ratios. Optimal conditions for LDH activity were observed when immobilized onto the NPLs, where the k_{cat} increased to 32.3 s^{-1} at the 1 LDH to 1 NPL ratio (2.4× or 240%). In general, the K_M values for PykA and LDH

Table 1. Estimated enzymatic kinetic parameters for LDH and PykA for mixed NPL and 525 QD systems

Enzyme: [NP]	V_{max} (nM × s^{-1})	k_{cat} (sec^{-1})	K_M (mM)	k_{cat}/K_M (mM^{-1} × s^{-1})
PykA				
PykA only	63.0 ± 1	25.0 ± 0.1	1.3 ± 0.1	$2.0 \times 10^{-5} \pm 2 \times 10^{-6}$
1.25 nM 525 QDs	14.0 ± 1	5.7 ± 0.1	1.4 ± 0.1	$4.1 \times 10^{-6} \pm 4 \times 10^{-7}$
1.25 nM 625 QDs	5.4 ± 0.2	2.2 ± 0.1	2.0 ± 0.2	$1.1 \times 10^{-6} \pm 1 \times 10^{-7}$
1.25 nM 641 QDs	2.6 ± 0.1	1.1 ± 0.1	1.5 ± 0.1	$7.0 \times 10^{-7} \pm 2 \times 10^{-8}$
1.25 nM NPLs	16.0 ± 1	6.4 ± 0.1	1.5 ± 0.2	$4.2 \times 10^{-6} \pm 5 \times 10^{-7}$
1 nM 525 QD/0.25 nM NPL	24.0 ± 1	9.7 ± 0.1	1.0 ± 0.1	$9.8 \times 10^{-6} \pm 8 \times 10^{-7}$
0.75 nM 525 QD/0.5 nM NPL	25.0 ± 1	10.0 ± 0.1	1.2 ± 0.2	$8.3 \times 10^{-6} \pm 1 \times 10^{-6}$
0.25 nM 525 QD/1 nM NPL	25.0 ± 2	10.0 ± 0.1	1.1 ± 0.2	$9.1 \times 10^{-6} \pm 9 \times 10^{-7}$
LDH				
LDH only	33.0 ± 3	13.3 ± 0.2	0.9 ± 0.4	$1.5 \times 10^{-5} \pm 7 \times 10^{-6}$
1.25 nM 525 QDs	45.0 ± 1	17.9 ± 0.1	0.9 ± 0.4	$2.0 \times 10^{-5} \pm 7 \times 10^{-6}$
1.25 nM 625 QDs	58.0 ± 3	23.1 ± 0.2	1.0 ± 0.2	$2.3 \times 10^{-5} \pm 4 \times 10^{-6}$
1.25 nM 641 QDs	7.7 ± 0.3	3.07 ± 0.1	0.8 ± 0.1	$3.8 \times 10^{-6} \pm 7 \times 10^{-7}$
1.25 nM NPLs	71.0 ± 4	28.6 ± 0.3	0.2 ± 0.2	$1.7 \times 10^{-5} \pm 2 \times 10^{-6}$
1 nM 525 QD/0.25 nM NPL	57.0 ± 1	23.0 ± 0.1	1.3 ± 0.1	$1.8 \times 10^{-5} \pm 1 \times 10^{-6}$
0.75 nM 525 QD/0.5 nM NPL	53.0 ± 1	21.1 ± 0.1	1.1 ± 0.2	$1.9 \times 10^{-5} \pm 2 \times 10^{-6}$
0.25 nM 525 QD/1 nM NPL	53.0 ± 2	21.1 ± 0.1	1.2 ± 0.2	$1.8 \times 10^{-5} \pm 4 \times 10^{-6}$

Final enzyme concentration: Throughout the table, the ratio of enzyme per QD was maintained at 2, where 2.5 nM enzyme was used with a total mixed or unmixed QD/NPL concentration of 1.25 nM. Enzyme only = free enzyme in solution, no NP present. All kinetic values are qualified as apparent.

decreased as compared with free enzyme meaning that increases in k_{cat}/K_M were mostly not seen. These results are in accordance with our previous findings *albeit* with some variability due to altered assay conditions for enzyme concentration, buffer, and pH (see the supplementary information for a full description of assay conditions).[38] Since our goal was to examine the display of enzymes on mixed-NP scaffolds, we next examined the individual activity of PykA and LDH at a ratio of two enzymes per NP in representative samples consisting of three different ratios of 525 QD to NPL, see Table 1 and Figure S8. For PykA, $k_{cat} = 13.3\ s^{-1}$ was again decreased, but interestingly, not as much as when displayed on either the 525 QDs or NPLs at the same ratio. In contrast, LDH activity was found to be better than all configurations excepting the largest display ratio of 8 LDH to 525 QD and then less than that seen with NPL alone except for the largest ratio of 8 again.

Comparison of channeling activity with individual QD sizes and NPLs

After establishing the full kinetic profile for both PykA and LDH across the four different NPs, we then investigated how intermediary channeling between these two enzymes varies across nanoclusters formed with the individual materials. To do this, we examined how the apparent k_{flux} varies for the two-enzyme cascade across the 525, 625, and 641 QDs. The NPLs were also examined, but at a lower overall concentration relative to the QDs due to the extended flat surface area of the NPL material and also its propensity toward precipitation at higher working concentrations in clusters.[20,40] Again, these assays monitored NAD^+ formation via absorbance on a microtiterwell plate reader. Since the catalytic rate of PykA was twice as fast as LDH, all experiments aimed to analyze channeling were done with twice the amount of LDH relative to PykA such that the initial rates of each enzyme were well matched.[38]

The panels in Figures 3A–3D show representative data for the 641 QDs and the NPLs at constant enzyme concentration, which corresponded to the worst and best performing materials, respectively, in this case. Figure 3A highlights traces of NAD^+ concentration change vs. time for the two-enzyme cascade at increasing concentrations of 641 QD with 450 μM PEP, while Figure 3C shows the same data for the NPLs collected with lower NPL concentrations. Figure 3B plots k_{flux} showing initial rates of NAD^+ conversion for the two-enzyme cascade across increasing amounts of 641 QD used vs. increasing concentrations of PEP, while Figure 3D shows the same data for the NPLs. Analogous data were collected from the 525 and 625 QDs, see Figures S9 and S10, respectively. Analyzing the changes in k_{flux} for the two-enzyme cascade assembled to the three different QDs at increasing concentrations while the concentrations of PykA and LDH remained constant across increasing concentrations of PEP (Figures 3B, S9, and S10), it is clear that the overall k_{flux} increases somewhat linearly with increasing QD concentration. These results align well with what we have previously observed, where increasing the concentration of QD induced larger cluster formation and allowed for higher enzyme incorporation per cluster yielding an overall more efficient channeling mechanism.[20]

Using the NPLs in the two-enzyme cascade assembly, NAD^+ formation over time also showed a dependence on NPL concentration while the overall k_{flux} increased linearly with increasing NPL concentration ranging from 0.25 up to 0.75 nM (Figure 3D). Notably, at 1 nM NPL there is a clear suppression in activity and overall k_{flux}. We believe this was the result of forming clusters, which were too large to remain stable in solution hence our trend

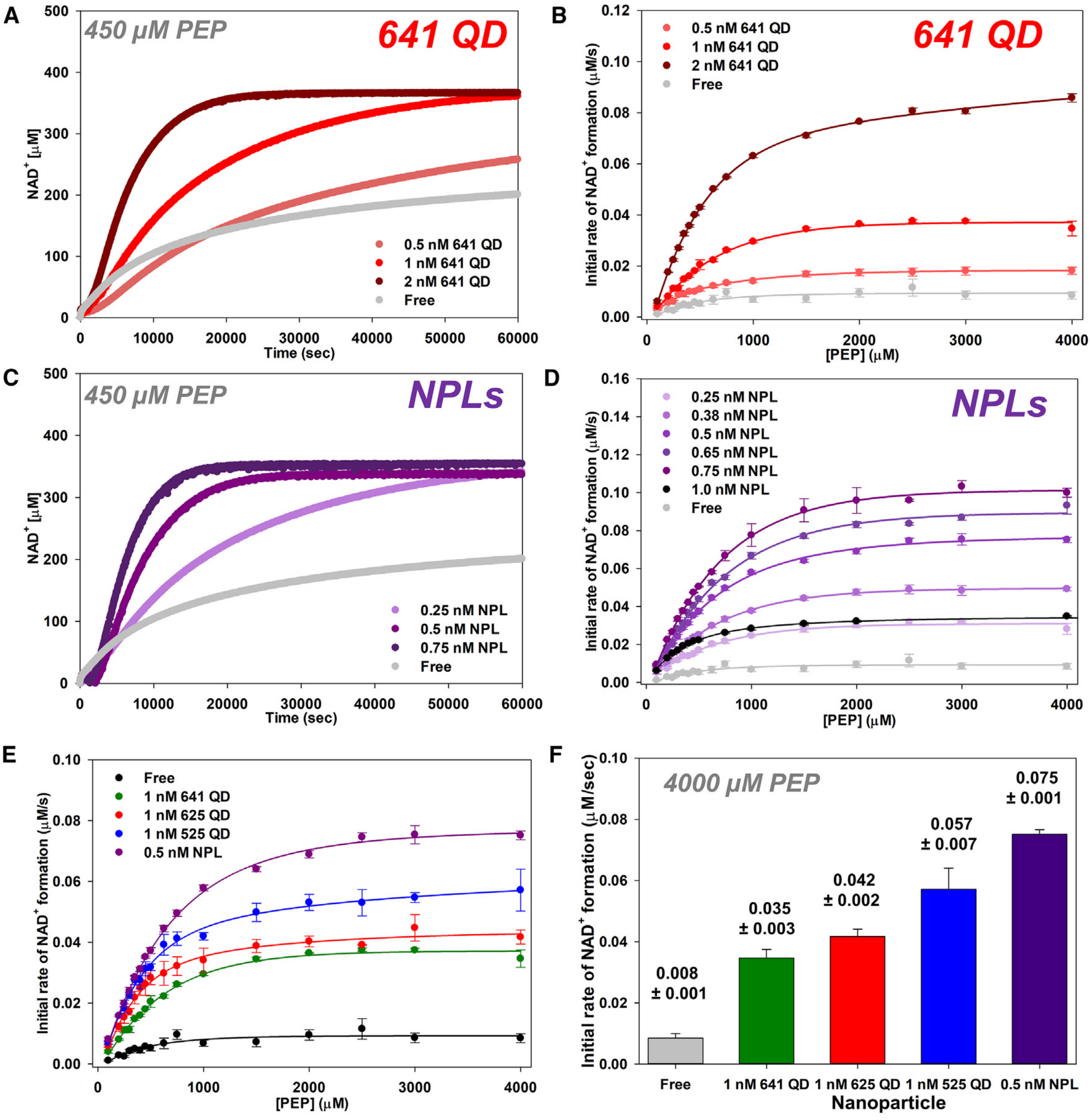

Figure 3. Kinetic enhancement from channeling in the two-enzyme cascade across different individual nanoparticles

(A) Representative progress curves of NAD^+ concentration vs. time at increasing concentrations of 641 QDs with 450 μM PEP.

(B) Plots of k_{flux} showing initial rates of NAD^+ conversion across increasing 641 QD used in self-assembly vs. increasing PEP concentration.

(C) Representative progress curves of NAD^+ concentration vs. time for increasing NPL concentrations with 450 μM PEP.

(D) Progress curves of k_{flux} showing initial rates of NAD^+ conversion for the two-enzyme cascade across increasing amounts of NPL used in the self-assembly vs. increasing concentrations of PEP. Enzyme concentration held constant in each assay while QD concentration varied.

(E) Plots of k_{flux} showing initial rates of NAD^+ conversion comparing free enzymes with individual 525 QDs, 625 QDs, 641 QDs, and NPLs used in the self-assembly vs. increasing concentrations of PEP.

(F) Plot of the initial rate of NAD^+ formation at 4,000 μM PEP comparing the free enzymes vs. the individual 525 QDs, 625 QDs, 641 QDs, and NPLs used in the self-assembly of PykA and LDH. Enzyme concentrations held constant in each assay. Full assay descriptions with similar data collected from the 525 and 625 QDs in the supplemental information. Data points from replicate samples and standard deviations were <15% in all cases. Trend lines to aid the eye are included and are not necessarily the MM fits.

of adjusting for this by lowering the working NPL concentrations in a manner similar to what we have reported previously.[20] Overall, when comparing the four different NP materials, each individual nanomaterial outperforms the activity that can be observed with the free enzymes under otherwise analogous conditions and a distinct relationship between decreasing QD size and increased k_{flux} can be observed when comparing the three QDs at a 1-nM concentration (Figure 3E). Comparably, half the amount of NPL can be used to achieve a greater overall k_{flux} relative to the three different QDs (Figure 3E, purple). Analyzing the

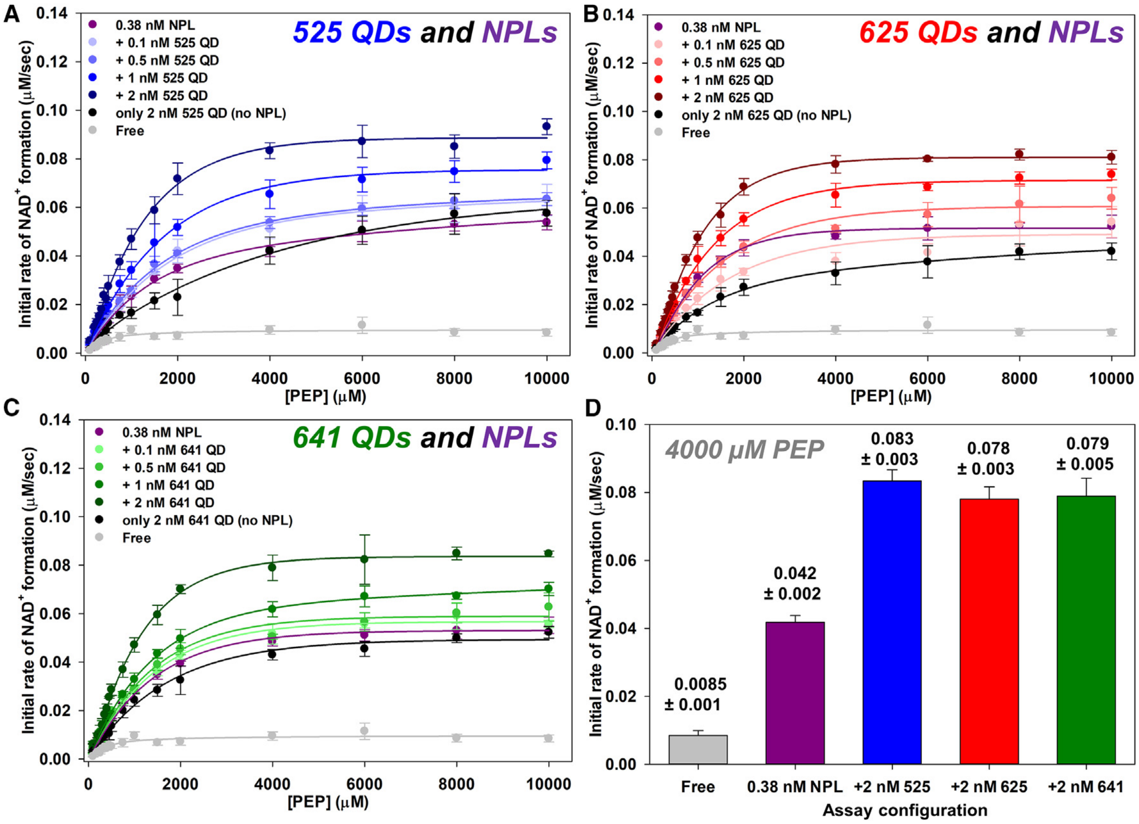

Figure 4. Changes in k_{flux} in the two-enzyme cascade as the result of mixed QD-NPL clusters engaged in channeling

(A) Plots of k_{flux} showing initial rates of NAD^+ conversion with 0.38 nM NPL and increasing amounts of 525 QD used in the self-assembly vs. increasing concentrations of PEP.

(B) Plots of k_{flux} showing initial rates of NAD^+ conversion with 0.38 nM NPL and increasing amounts of 625 QD used in the self-assembly vs. increasing concentrations of PEP.

(C) Plots of k_{flux} showing initial rates of NAD^+ conversion with 0.38 nM NPL and increasing amounts of 641 QD used in the self-assembly vs. increasing concentrations of PEP.

(D) Bar graph illustrating the initial rate of NAD^+ formation that is achieved at 4,000 μM PEP with only 0.38 nM NPL vs. 0.38 nM NPL with 2 nM of each of the QDs used in the self-assembly process along with the same concentration of free enzyme. Enzyme concentrations held constant in each assay while NP concentrations varied. Full assay descriptions in the supplemental information. Data points from replicate samples and standard deviations were <15% in all cases. Trend lines to aid the eye are included and are not necessarily the MM fits.

initial rate data in Figure 3E at 4,000 μM PEP and plotting the observed initial rate for the free enzyme system and NP-assembled systems, a linear correlation exits from the free enzyme system through the three QDs of decreasing size (641→625→525 QD) at 1 nM concentration to the NPLs at a concentration of 0.5 nM, where the largest initial rate is observed (Figure 3F). For the NPLs, the initial rate is enhanced >8× that of the free enzyme.

Channeling within QD/NPL mixed assemblies

We next sought to combine the two-enzyme cascade with both QDs and NPLs into mixed-NP assemblies to ascertain if it was possible to achieve a greater enhancement in channeling relative to that obtained with any of the single nanomaterial assemblies. The overarching goal was to identify if these assemblies would even optimize the underlying geometric packing and assembly components to create immobilized enzyme clusters that are even further enhanced for product formation beyond our current strategy of using singular nanomaterial types as the scaffolding that provides enzyme crosslinking. To do this, a series of multiparametric experiments testing the different NP-enzyme assembly combinations was performed.

First, we looked to combine each of the individual different-sized QDs with the NPLs for the self-assembly of the PykA-LDH system. Having already observed that lower amounts of the NPL could be used in this two-enzyme cascade to achieve an enhancement in activity (Figure 3D), we started with 0.38 nM NPL and systematically altered the self-assembly reactants to incorporate increasing amounts of 525 QD (0–2 nM). As can be observed in Figures 4A and S11A, when the 525 QDs are added increasing the NP concentration beyond the 0.38 nM NPL, a subsequent increase in the overall k_{flux} is observed. This increase in k_{flux} is greater than what is observed with either of the two NPs independently, suggesting that combining the two materials alters cluster formation in such a way that it improves the intermediary channeling process in the two-enzyme

cascade. The same data were obtained with the same amount of enzyme and 0.38 nM NPL when adding increasing concentrations of either 625 QD (Figures 4B; S11B) or 641 QD (Figures 4C; S11C) during the NP-enzyme self-assembly process. Figure S12 shows representative TEM micrographs taken of the mixed QD-NPL structures. These results demonstrated a similar trend to what was observed in the 525 QD titration with 0.38 nM NPL. Notably, if you compare the initial rate of the two-enzyme system assembled to 0.38 nM NPL vs. that of the two-enzyme system assembled to 0.38 nM NPL mixed with 2 nM of either 525, 625, or 641 QDs at 4,000 μM PEP, you observe a 2× increase in initial rate with the addition of 2 nM QD in the self-assembly process (Figure 4D). However, this 2× increase in initial rate appears to be independent of QD size, which is clearly in contrast with what we observed for the independent QD assemblies, described above.

Next, a series of assays were performed attempting to mix the different QD sizes in the self-assembly process in a similar manner as done for the individual QDs and NPLs described above. This was undertaken to see if any combination of differentially sized QDs in clusters would offer an enhancement in k_{flux} that was greater than when only one QD size was used. These results are illustrated as a series of plots of the initial rate of NAD^+ formation vs. increasing concentrations of PEP shown in Figures 5A, 5B, S13, and S14. For each of the three differently sized QDs, a lower concentration of one QD was used (0.38 nM) in the self-assembly process similar to the above for the NPLs with added increasing concentrations of a differently sized QD (0–2 nM). Cumulatively, these results are more suggestive of an effective increase in total nanomaterial concentration causing an enhancement in overall k_{flux}. Intuitively, these results are not unexpected, as under these conditions only the QD sizes are being changed, whereas, with the NPLs mixed with differentially sized QDs there exists more significant changes in both size and shape, which one would expect should have a greater impact on the geometric packing within the immobilized enzyme cluster formed during the self-assembly process. To further confirm and expand on this, we then performed a series of assays where the total concentration of nanomaterial present in the reaction was held constant at 1 nM and then their ratio relative to each was systematically changed (Figures 5C–5F). As shown in Figure 5C, when combining the 525 QDs with the NPLs and maintaining a constant total NP concentration of 1 nM, a distinct enhancement in the overall k_{flux} is created, which is independent of the total amount of NP used in the self-assembly process. However, when the same experiment is performed combining only the QDs of different sizes (Figures 5D–5F), the overall enhancement in k_{flux} becomes much less pronounced relative to what was observed with the initial NPL-525 QD mix. Overall, the best improvements are obtained when adding NPLs with the QDs in the mixed-NP clusters. We previously noted that the best increases in channeled k_{flux} were obtained with use of the smallest 525 QDs or the NPLs and the same remains true here with individual enzymes, coupled enzymes, and also amongst the mixed QD-NPL systems tested (Figure 5).[20] These improvements can be accessed either by increasing the total amount of NP concentration to presumably induce formation of larger nanoclusters or when keeping NP concentration constant but varying the QD-to-NPL ratio present. That such improvements increase beyond what was obtained with NPLs alone also suggests that the mixed NP-enzyme systems are perhaps able to extend or increase colloidal stability of the resulting aggregate versus that of NPL alone.

Application to a seven-enzyme cascade

To highlight that the above mixed NP-scaffolded approach has applicability beyond the prototypical two-enzyme system characterized above, we performed preliminary experiments with the seven-enzyme cascade that catalyzes the conversion of glucose to 3-phosphoglycerate (3-PG) as part of oxidative glycolysis, see Figure 6A. This cascade had recently been utilized to extensively characterize the channeling phenomena itself when formed into the requisite NP-enzyme clusters.[20] All studies in that report utilized a single NP type for each assay and also confirmed that NPLs could induce more efficient channeling than clusters assembled with spherical QDs. As above, the assay was monitored by measuring the conversion of NAD^+ to NADH by glyceraldehyde-3-phosphate dehydrogenase (GPD) via changes to the cofactor's absorption at the penultimate enzymatic step on a microtiter well plate reader. The additional conversion step of adding phosphoglycerate kinase into this cascade is due to the strongly negative free energy (ΔG) of this reaction, which helps pull the reaction flux forward *vice* that of the GPD, which is far more positive and favors the reverse gluconeogenic reaction direction.[20]

Figures 6B and 6C present representative data where the seven enzymes were combined with 525 QDs, NPLs, or select mixed ratios of both while maintaining a constant total NP concentration of 100 nM. The higher concentration of NPs is utilized here as initial testing showed that the QDs and NPLs tolerated the seven enzyme (7E) system at these concentrations without precipitating. The concentrations and ratios of the enzymes and the NPs utilized in this assay were drawn directly from the previous study and are described along with the assay format in the STAR methods.[20] Although using the NPLs alone as the scaffolding material performs quite well, use of 50 nM:50 nM and 25 nM:75 nM ratios of QD:NPL improve the rate of coupled flux beyond that of the NPL alone converting 10%–20% more NADH in the same time period. This may seem rather modest, but it is important to appreciate that this improvement was obtained from just a first test assay without any of the extensive parametric testing that was done with the two-enzyme system above. More importantly, this improvement also corresponds to a 4- to 5-fold more NADH conversion than the QDs are capable of by themselves when used as the sole scaffolding material at the same concentration. Another aspect to note is that total number of $(His)_6$ tags present is estimated to be ~95 nM, which is on par with the total number of NP present. Enzymes in the seven-enzyme cascade are a mix of monomers, dimers, and tetramers suggesting the total NP concentration should be in slight excess to the total concentration of $(His)_6$ tags so as to achieve nanocluster formation when further considering NP size and shape.

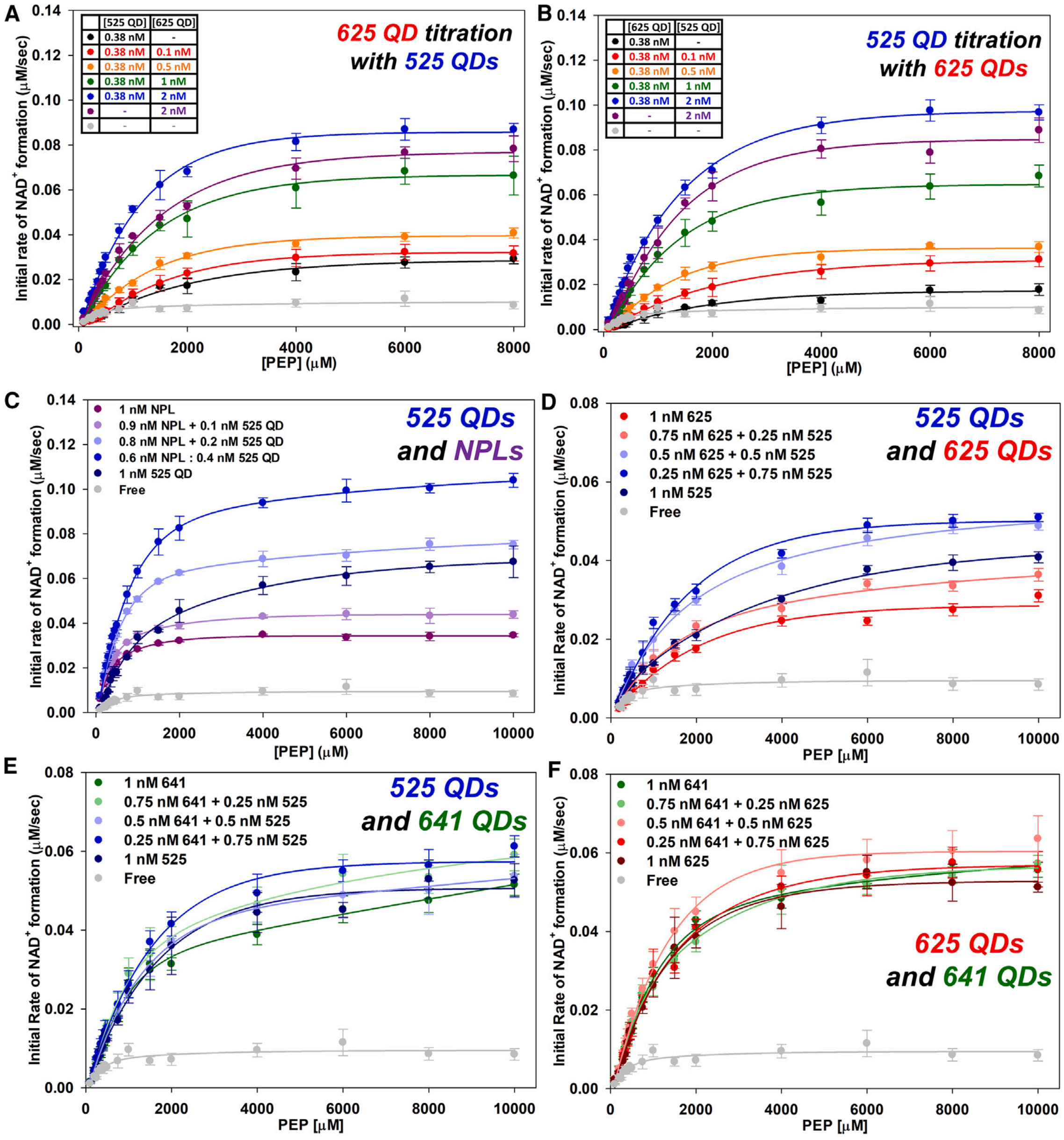

Figure 5. Changes in k_{flux} in the two-enzyme cascade from mixing QDs of different sizes or QDs with NPLs at a constant overall concentration

(A) Plots of k_{flux} showing initial rates of NAD^+ conversion with 0.38 nM 525 QD and increasing 625 QD used in self-assembly vs. increasing PEP concentrations.
(B) Plots of k_{flux} showing initial rates of NAD^+ conversion for 0.38 nM 625 QD and increasing amounts of 525 QD in the self-assembly vs. increasing PEP concentrations.
(C) Plots of k_{flux} showing initial rates of NAD^+ conversion with constant 1 nM nanoparticle that varied by mixing 525 QDs and NPLs at different ratios used in the self-assembly vs. increasing PEP concentrations.
(D) Plots of k_{flux} showing initial rates of NAD^+ conversion with constant 1 nM nanoparticle varied by mixing 525 QDs and 625 QDs at different ratios used in the self-assembly vs. increasing PEP concentrations.
(E) Plots of k_{flux} showing initial rates of NAD^+ conversion with constant 1 nM nanoparticle varied by mixing 525 QDs and 641 QDs at different ratios used in the self-assembly vs. increasing PEP concentrations.
(F) Plots of k_{flux} showing initial rates of NAD^+ conversion with a constant 1 nM nanoparticle varied by mixing 625 QDs and 641 QDs at different ratios used in the self-assembly vs. increasing PEP concentrations. Enzyme and overall nanomaterial concentration held constant while relative NP ratios to each other varied. Full assay descriptions in the supplemental information. Data points from replicate samples and standard deviations were <15% in all cases. Trend lines to aid the eye are included in (A)–(F), these are not necessarily the MM fits.

CellPress
OPEN ACCESS

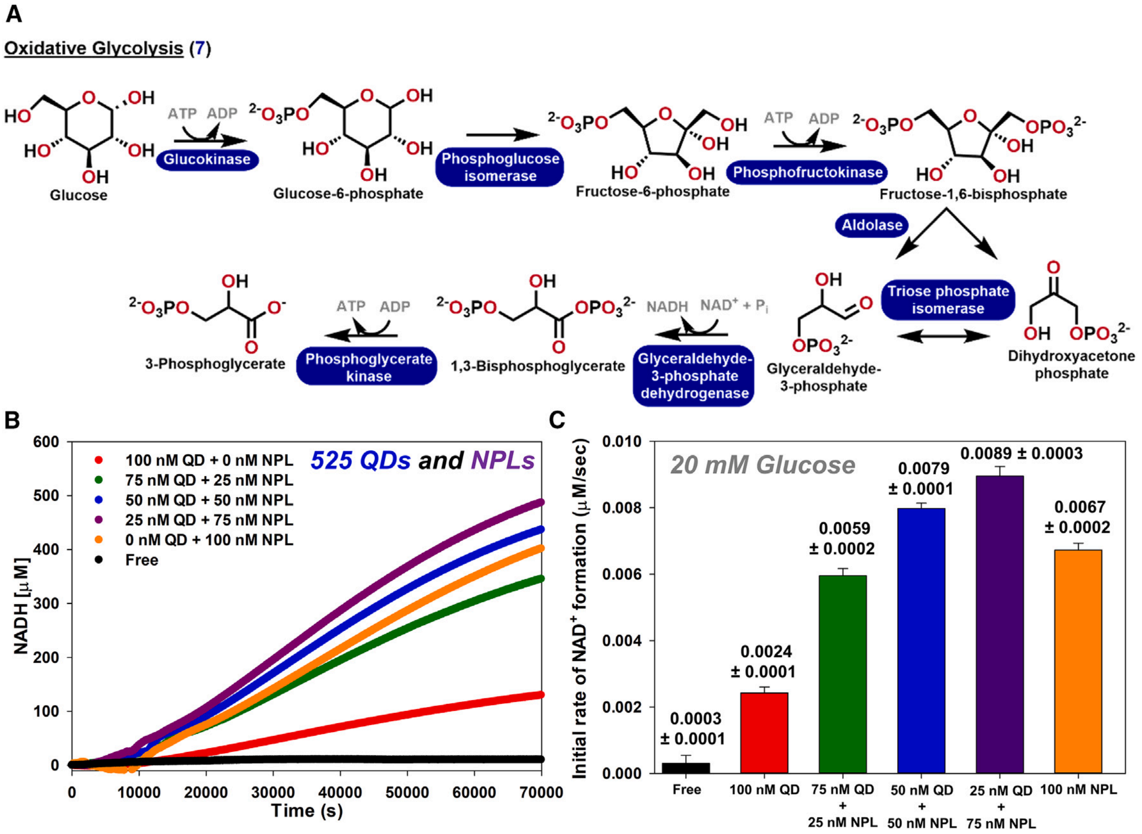

Figure 6. Changes in k_{flux} in a seven-enzyme cascade from mixing QDs with NPLs at a constant overall nanoparticle concentration

(A) Seven-enzyme pathway used to convert glucose to 3-phosphoglycrate, → indicates enzymatically catalyzed step(s). Chemical structures of the substrate, intermediaries, and final product.

(B) Progress curves of NADH production with a constant NP concentration of 100 nM varied by mixing 525 QDs and NPLs at different ratios used in the self-assembly. Each line represents the average of four replicates.

(C) Plot of initial rate of NAD^+ production with a constant NP concentration of 100 nM varied by mixing 525 QDs and NPLs at different ratios used in the self-assembly. Reaction conditions include 15 mM $MgCl_2$, 7.5 mM ATP, 7.5 mM ADP, 10 mM glucose, 4 mM dibasic/monobasic phosphate, 1.125 mM NAD^+, and 250 mM HEPES (pH 8). Final enzyme concentration: 2.75 nM glucokinase (GlK), 0.5 nM phosphoglucose isomerase (PGI), 4.5 nM phosphofructokinase (PFK), 6 nM fructose-bisphosphate aldolase (FBA), 0.5 nM triose phosphate isomerase (TPI), 13.5 nM glyceraldehyde-3-phosphate dehydrogenase (GPD), and 3.75 nM phosphoglycerate kinase (PGK).

DISCUSSION

The growing field of SynBio in all its different manifestations continues to drive extraordinary interest in harnessing the full synthetic potential of enzymes toward replacing many fossil fuel-based chemical feedstocks with more environmentally cleaner, safer, and renewable materials. Critically, the bulk of enzymatic chemistry toward biosynthesizing new non-natural products will occur primarily outside of cells due to toxicity issues, and thus must be found to significantly enhance this reaction format. Since most enzymatic chemistry occurs in the context of multi-enzymatic cascades, channeling represents a potentially robust methodology to impart efficiency to enzymes as they make complex molecules while also reducing the environmental impact of production. In order for successful channeling to become a routine approach across various enzymatic platforms, the principles that guide and dictate successful channeling must be well understood.[11,17–19] Since fusing enzymes together or chemically attaching them to different organic scaffolding materials has not yet been proven as a reliable way to access channeling, simpler and more efficient methodologies are needed right now.[19] Our approach toward accessing enzymatic channeling is to utilize NPs as inorganic nanoscale scaffolds that crosslink the enzymes into dense clusters where probabilistic channeling occurs.

Although we utilize in-house custom-prepared QD materials, we note that QDs are commercially available with a variety of other surface preparations and that the same metal-affinity driven self-assembly will occur with QDs that display some type of carboxylated surface chemistry.[48–50] The carboxylated QDs in essence chemically mimic an NTA-functionalized surface with the only requirement being to load the surface molecules with some Ni^{2+} or similarly functional divalent cation (Co, Cr, Mn, etc.). Similar enzyme bioconjugation and NP-crosslinking toward channeling should be feasible with other surface carboxylated NPs along with other NPs displaying the requisite NTA groups.[32,33,51] Indeed, channeling with the same seven-enzyme

system as utilized above was achieved within clusters formed with commercially carboxylated QDs, NTA-functionalized gold NPs, and even multigenerational carboxylated dendrimers.[20] Since metallic NPs are commonly utilized as catalysts in chemistry, we also note that we tested each of the QD and NPL materials utilized in this study without enzymes present for their ability to catalyze any of the reactions. All of these studies were negative (data not shown).

Here, we have analyzed channeling within a previously described two-enzyme cascade, containing LDH and PykA, for the conversion of PEP to lactic acid.[38] Extending from previous studies utilizing QDs as scaffolds for channeling, we focus on utilizing QDs of increasing size, 2D NPLs, and especially mixed QD-NPL systems to identify cluster assembly conditions enabling further enhancement of product formation via optimization of the underlying cluster architecture. We analyzed the MM kinetics for each enzyme in the cascade across all four NP materials at varying ratios and confirmed successful self-assembly of the enzymes to the NPs via agarose gel mobility assays. Using fixed enzyme ratios optimized for channeling, the change in k_{flux} was analyzed across all four NPs at varying concentrations to compare the effect of QD size within a channeled system. TEM data analyzed changes in NP cluster distribution across different NP concentrations for all four NPs, where it was found that NPLs produced larger clusters at lower overall NP concentration relative to the differently sized QDs. Mixed-NP assemblies were then explored to observe subsequent changes in the k_{flux} of the channeled system and self-assembly of the mixed-NP system was confirmed from additional TEM data. Overall, we demonstrated that by mixing different NP materials, a >10× improvement in k_{flux} is observed relative to free enzymes, which is also 2× and, in some cases, greater than that achieved with any of the individual NPs. Last, we apply the mixed-NP assembly approach to an extended seven-enzyme cascade where enhanced channeling was also initially demonstrated without doing any parametric characterization. Our results further demonstrated that utilizing the smallest 525 QDs and NPLs where even a small amount of NPL replaced the QD provided for the most consistently increased improvements in k_{flux}. Consistent with this, use of NPLs and smaller QDs/NPs have provided for the best channeled activity in previous experiments.[20,36,38] Interestingly, smaller QDs/NPs also tend to more consistently provide for the greatest enhancement in individual enzyme activity that is many times associated with NP display.[30,35,47,52–58] We also note that the catalytic flux in extended enzymatic cascades can be further improved by undertaking detailed numerical simulations of the coupled reactions in the context of the MM model, see for example Breger et al.[20] and Vranish et al.[38]

The simplicity of assembling these channeled cascaded systems, which requires just mixing of the NPs and necessary enzymes, suggests that this approach should be readily applicable to many other multienzyme cascades. Moreover, the applicable chemical space that these systems can access should be significantly larger than that afforded by cell-based SynBio. Both non-natural substrates and intermediaries can be readily utilized in this format without concern for cellular toxicity. There is also no reason preventing both eukaryotic- and prokaryotic-sourced enzymes being jointly utilized in these channeled clusters, which should eliminate the need to back-engineer the former type of enzymes for expression in the latter type of cells. Coming back full circle to cell-based SynBio, the approach described here is not meant to replace this in any sense but rather to function as a complementary approach that can undertake the challenging task of synthesizing many of the target molecules that cell-based systems either cannot or that they will struggle with.

Limitations of the study

Given that the research described is still in its initial stages, there are remaining challenges to be addressed. It is not clear how many enzymatic steps can be directly incorporated and achieve channeling in these clusters. The previous study utilizing glycolytic enzymes achieved a 10-enzyme channeled system.[20] There will also be kinetic, thermodynamic, and energetic barriers that will not be favorable toward creating extended channeled systems. In the previous example where oxidative glycolysis was extended from seven to an 11-enzyme cascade processing glucose to lactate, unfavorable kinetics precluded channeling from being accessed directly.[20] However, overall channeled activity was maintained by splitting the system after the seventh enzyme, purifying 3-PG intermediary from the upstream sub-cascade, and feeding it as a concentrated substrate to the downstream four-enzyme sub-cascade that converted it to lactate with channeling. Extending from this, the possibility exists for having two different nanoclustered enzyme cascades present in the same reaction where the downstream cascade is activated when sufficient product from the upstream cascade is produced such that it can be bound by the first enzyme of the downstream cascade.

Characterization of the NP-enzyme clusters still remains somewhat limited beyond gel separations, TEM, and simulations of the DLA assembly process.[20] This is due to the lack of metrologies available for characterizing the ensemble distribution of structures formed from such biological-inorganic nanoscale materials.[59,60] Förster resonance energy transfer (FRET) and dynamic light scattering (DLA) analysis have also seen preliminary application to characterizing these hybrid nanoclustered assemblies.[20] Another limitation is the requirement for NADH and ADP cofactors. Cofactors are challenging to chemically produce and therefore are commercially expensive. Additionally, the reactions of interest typically require an excess of cofactors for successful product formation. Fortunately, enzymatic strategies for efficient cofactor recycling have gained increased interest recently. Cofactor recycling is advantageous because it reduces the amount of cofactor required to drive product formation.[61–63] Importantly, cofactor recycling can be implemented using nanoclustered enzyme constructs that themselves exploit channeling making this even more efficient.[46] Last, we appreciate the toxicity concerns about utilizing semiconductor QDs containing Cd.[64] Opportunely, earth abundant, non-toxic ZnS-based QDs with similar size and surface properties, i.e., allowing metal-affinity coordination by an enzyme's $(His)_6$, should be amenable to similar application.[39,65]

STAR★METHODS

Detailed methods are provided in the online version of this paper and include the following:

- KEY RESOURCES TABLE
- RESOURCE AVAILABILITY
  - Lead contact
  - Materials availability
  - Data and code availability
- EXPERIMENTAL MODEL AND STUDY PARTICIPANT DETAILS
  - Pyruvate kinase II (PykA)
  - Lactate dehydrogenase (LDH)
- METHOD DETAILS
  - Quantum dots
  - Physicochemical characterization
  - Kinetic assays
- QUANTIFICATION AND STATISTICAL ANALYSIS

SUPPLEMENTAL INFORMATION

Supplemental information can be found online at https://doi.org/10.1016/j.crmeth.2024.100764.

ACKNOWLEDGMENTS

The authors acknowledge the Office of Naval Research (award N0001421WX01580), the US Naval Research Laboratory (NRL); the NRL Nanoscience Institute; the National Institute of Food and Agriculture, US Department of Agriculture award #2020-67021-31254; and the Strategic Environmental Research and Development Program (SERDP) award # WP21-1073 New Start Project (W74RDV03497375).

AUTHOR CONTRIBUTIONS

I.L.M. and S.L.H. provided initial concepts along with experimental design. S.L.H., J.C.B., and I.L.M. undertook experimental analysis and interpretation. C.M.G. obtained TEM images used in analyses and interpretations. K.S. and M.H.S. synthesized all nanoparticles used. All authors contributed to the writing and approval of the final manuscript.

DECLARATION OF INTERESTS

The authors declare no competing interests.

Received: November 26, 2023
Revised: February 19, 2024
Accepted: April 12, 2024
Published: May 6, 2024

REFERENCES

1. El Karoui, M., Hoyos-Flight, M., and Fletcher, L. (2019). Future trends in synthetic biology-a report. Front. Bioeng. Biotechnol. *7*, 175.
2. Bowie, J.U., Sherkhanov, S., Korman, T.P., Valliere, M.A., Opgenorth, P.H., and Liu, H. (2020). Synthetic biochemistry: The bio-inspired cell-rree approach to commodity chemical production. Trends Biotechnol. *38*, 766–778.
3. Brooks, S.M., and Alper, H.S. (2021). Applications, challenges, and needs for employing synthetic biology beyond the lab. Nat. Commun. *12*, 1390.
4. Church, G.M., Elowitz, M.B., Smolke, C.D., Voigt, C.A., and Weiss, R. (2014). Realizing the potential of synthetic biology. Nat. Rev. Mol. Cell Biol. *15*, 289–294.
5. Clomburg, J.M., and Gonzalez, R. (2010). Biofuel production in Escherichia coli: the role of metabolic engineering and synthetic biology. Appl. Microbiol. Biotechnol. *86*, 419–434.
6. Smanski, M.J., Zhou, H., Claesen, J., Shen, B., Fischbach, M.A., and Voigt, C.A. (2016). Synthetic biology to access and expand nature's chemical diversity. Nat. Rev. Microbiol. *14*, 135–149.
7. Helfrich, E.J.N., Lin, G.M., Voigt, C.A., and Clardy, J. (2019). Bacterial terpene biosynthesis: challenges and opportunities for pathway engineering. Beilstein J. Org. Chem. *15*, 2889–2906.
8. Voigt, C.A. (2020). Synthetic biology 2020-2030: six commercially-available products that are changing our world. Nat. Commun. *11*, 6379.
9. Clarke, L., and Kitney, R. (2020). Developing synthetic biology for industrial biotechnology applications. Biochem. Soc. Trans. *48*, 113–122.
10. Lin, G.-M., Warden-Rothman, R., and Voigt, C.A. (2019). Retrosynthetic design of metabolic pathways to chemicals not found in nature. Curr. Opin. Struct. Biol. *14*, 82–107.
11. Hooe, S.L., Ellis, G.A., and Medintz, I.L. (2022). Alternative design strategies to help build the enzymatic retrosynthesis toolbox. RSC Chem. Biol. *3*, 1301–1313.
12. Opgenorth, P.H., Korman, T.P., and Bowie, J.U. (2014). A synthetic biochemistry molecular purge valve module that maintains redox balance. Nat. Commun. *5*, 4113.
13. Abernathy, M.H., He, L., and Tang, Y.J. (2017). Channeling in native microbial pathways: Implications and challenges for metabolic engineering. Biotechnol. Adv. *35*, 805–814.
14. Jørgensen, K., Rasmussen, A.V., Morant, M., Nielsen, A.H., Bjarnholt, N., Zagrobelny, M., Bak, S., and Møller, B.L. (2005). Metabolon formation and metabolic channeling in the biosynthesis of plant natural products. Curr. Opin. Plant Biol. *8*, 280–291.
15. Vélot, C., Mixon, M.B., Teige, M., and Srere, P.A. (1997). Model of a quinary structure between Krebs TCA cycle enzymes: A model for the metabolon. Biochemistry *36*, 14271–14276.
16. Zhao, X., Palacci, H., Yadav, V., Spiering, M.M., Gilson, M.K., Butler, P.J., Hess, H., Benkovic, S.J., and Sen, A. (2018). Substrate-driven chemotactic assembly in an enzyme cascade. Nat. Chem. *10*, 311–317.
17. Cornish-Bowden, A. (2012). Fundamentals of Enzyme Kinetics, 4th Edition (Wiley-Blackwell).
18. Wheeldon, I., Minteer, S.D., Banta, S., Barton, S.C., Atanassov, P., and Sigman, M. (2016). Substrate channelling as an approach to cascade reactions. Nat. Chem. *8*, 299–309.
19. Ellis, G.A., Klein, W.P., Lasarte-Aragonés, G., Thakur, M., Walper, S.A., and Medintz, I.L. (2019). Artificial multienzyme scaffolds: Pursuing in vitro substrate channeling with an overview of current progress. ACS Catal. *9*, 10812–10869.
20. Breger, J.C., Vranish, J.N., Oh, E., Stewart, M.H., Susumu, K., Lasarte-Aragonés, G., Ellis, G.A., Walper, S.A., Díaz, S.A., Hooe, S.L., et al. (2023). Self assembling nanoparticle enzyme clusters provide access to substrate channeling in multienzymatic cascades. Nat. Commun. *14*, 1757.
21. Zhang, Y., and Fernie, A.R. (2021). Metabolons, enzyme–enzyme assemblies that mediate substrate channeling, and their roles in plant metabolism. Plant Commun. *2*, 100081.
22. Dunn, M.F. (2012). Allosteric regulation of substrate channeling and catalysis in the tryptophan synthase bienzyme complex. Arch. Biochem. Biophys. *519*, 154–166.
23. Castellana, M., Wilson, M.Z., Xu, Y., Joshi, P., Cristea, I.M., Rabinowitz, J.D., Gitai, Z., and Wingreen, N.S. (2014). Enzyme clustering accelerates processing of intermediates through metabolic channeling. Nat. Biotechnol. *32*, 1011–1018.
24. Dueber, J.E., Wu, G.C., Malmirchegini, G.R., Moon, T.S., Petzold, C.J., Ullal, A.V., Prather, K.L.J., and Keasling, J.D. (2009). Synthetic protein scaffolds provide modular control over metabolic flux. Nat. Biotechnol. *27*, 753–759.
25. Kummer, M.J., Lee, Y.S., Yuan, M., Alkotaini, B., Zhao, J., Blumenthal, E., and Minteer, S.D. (2021). Substrate channeling by a rationally designed fusion protein in a biocatalytic cascade. JACS Au *1*, 1187–1197.

26. Chen, W.-H., Vázquez-González, M., Zoabi, A., Abu-Reziq, R., and Willner, I. (2018). Biocatalytic cascades driven by enzymes encapsulated in metal–organic framework nanoparticles. Nat. Catal. *1*, 689–695.

27. Benčina, M., Mori, J., Gaber, R., and Jerala, R. (2018). Metabolic channeling using DNA as a scaffold. In Synthetic Biology: Parts, Devices and Applications, C. Smolke, S.Y. Lee, J. Nielsen, and G. Stephanopoulos, eds. (Wiley), pp. 237–259.

28. Chatterjee, A., Mahato, C., and Das, D. (2021). Complex cascade reaction networks via cross β amyloid nanotubes. Angew. Chem., Int. Ed. *60*, 202–207.

29. Díaz-Caballero, M., Navarro, S., and Ventura, S. (2021). Functionalized prion-inspired amyloids for biosensor applications. Biomacromolecules *22*, 2822–2833.

30. Breger, J.C., Walper, S.A., Oh, E., Susumu, K., Stewart, M.H., Deschamps, J.R., and Medintz, I.L. (2015). Quantum dot display enhances activity of a phosphotriesterase trimer. Chem. Commun. *51*, 6403–6406.

31. Klein, W.P., Thomsen, R.P., Turner, K.B., Walper, S.A., Vranish, J., Kjems, J., Ancona, M.G., and Medintz, I.L. (2019). Enhanced catalysis from multienzyme cascades assembled on a DNA origami triangle. ACS Nano *13*, 13677–13689.

32. Algar, W.R., Prasuhn, D.E., Stewart, M.H., Jennings, T.L., Blanco-Canosa, J.B., Dawson, P.E., and Medintz, I.L. (2011). The controlled display of biomolecules on nanoparticles: A challenge suited to bioorthogonal chemistry. Bioconjugate Chem. *22*, 825–858.

33. Sapsford, K.E., Algar, W.R., Berti, L., Gemmill, K.B., Casey, B.J., Oh, E., Stewart, M.H., and Medintz, I.L. (2013). Functionalizing nanoparticles with biological molecules: Developing chemistries that facilitate nanotechnology. Chem. Rev. *113*, 1904–2074.

34. Sapsford, K.E., Tyner, K.M., Dair, B.J., Deschamps, J.R., and Medintz, I.L. (2011). Analyzing nanomaterial bioconjugates: A review of current and emerging purification and characterization techniques. Anal. Chem. *83*, 4453–4488.

35. Breger, J.C., Oh, E., Susumu, K., Klein, W.P., Walper, S.A., Ancona, M.G., and Medintz, I.L. (2019). Nanoparticle size influences localized enzymatic enhancement - A casestudy with phosphotriesterase. Bioconjugate Chem. *30*, 2060–2074.

36. Díaz, S.A., Choo, P., Oh, E., Susumu, K., Klein, W.P., Walper, S.A., Hastman, D.A., Odom, T.W., and Medintz, I.L. (2020). Gold nanoparticle templating increases the catalytic rate of an amylase, naltase, and glucokinase multienzyme dascade through substrate channeling independent of surface curvature. ACS Catal. *11*, 627–638.

37. Hooe, S., Breger, J., Dean, S., Susumu, K., Oh, E., Walper, S., Ellis, G.A., and Medintz, I.L. (2022). Benzaldehyde lyase kinetic improvements, potential channeling to alcohol dehydrogenase, and substrate scope when immobilized on semiconductor quantum dots. ACS Appl. Nano Mater. *5*, 10900–10911.

38. Vranish, J.N., Ancona, M.G., Oh, E., Susumu, K., Lasarte Aragonés, G., Breger, J.C., Walper, S.A., and Medintz, I.L. (2018). Enhancing coupled enzymatic activity by colocalization on nanoparticle surfaces: Kinetic evidence for directed channeling of intermediates. ACS Nano *12*, 7911–7926.

39. Susumu, K., Field, L.D., Oh, E., Hunt, M., Delehanty, J.B., Palomo, V., Dawson, P.E., Huston, A.L., and Medintz, I.L. (2017). Purple-blue-and green-emitting multishell alloyed quantum dots: synthesis, characterization, and application for ratiometric extracellular pH sensing. Chem. Mater. *29*, 7330–7344.

40. Ithurria, S., Tessier, M.D., Mahler, B., Lobo, R.P.S.M., Dubertret, B., and Efros, A.L. (2011). Colloidal nanoplatelets with two-dimensional electronic structure. Nat. Mater. *10*, 936–941.

41. Diroll, B.T., Guzelturk, B., Po, H., Dabard, C., Fu, N., Makke, L., Lhuillier, E., and Ithurria, S. (2023). 2D II–VI Semiconductor nanoplatelets: from material synthesis to optoelectronic integration. Chem. Rev. *123*, 3543–3624.

42. Susumu, K., Oh, E., Delehanty, J.B., Blanco-Canosa, J.B., Johnson, B.J., Jain, V., Hervey, W.J., 4th, Algar, W.R., Boeneman, K., Dawson, P.E., and Medintz, I.L. (2011). Multifunctional compact zwitterionic ligands for preparing robust biocompatible semiconductor quantum dots and gold nanoparticles. J. Am. Chem. Soc. *133*, 9480–9496.

43. Blanco-Canosa, J.B., Wu, M., Susumu, K., Petryayeva, E., Jennings, T.L., Dawson, P.E., Algar, W.R., and Medintz, I.L. (2014). Recent progress in the bioconjugation of quantum dots. Coord. Chem. Rev. *263–264*, 101–137.

44. Prasuhn, D.E., Deschamps, J.R., Susumu, K., Stewart, M.H., Boeneman, K., Blanco-Canosa, J.B., Dawson, P.E., and Medintz, I.L. (2010). Polyvalent display and packing of peptides and proteins on semiconductor quantum dots: Predicted versus experimental results. Small *6*, 555–564.

45. Witten, T.A., and Sander, L.M. (1981). Diffusion-limited aggregation, a kinetic critical phenomenon. Phys. Rev. Lett. *47*, 1400–1403.

46. Breger, J.C., Goldman, E.R., Susumu, K., Oh, E., Green, C.M., Hooe, S.L., Thakur, M., Medintz, I.L., and Ellis, G.A. (2023). Enzyme assembly on nanoparticle scaffolds enhances cofactor recycling and improves coupled reaction kinetics. Nanoscale *15*, 10159–10175.

47. Breger, J.C., Ancona, M.G., Walper, S.A., Oh, E., Susumu, K., Stewart, M.H., Deschamps, J.R., and Medintz, I.L. (2015). Understanding how nanoparticle attachment enhances phosphotriesterase kinetic efficiency. ACS Nano *9*, 8491–8503.

48. Boeneman, K., Delehanty, J.B., Susumu, K., Stewart, M.H., and Medintz, I.L. (2010). Intracellular bioconjugation of targeted proteins with semiconductor quantum dots. J. Am. Chem. Soc. *132*, 5975–5977.

49. Yao, H., Zhang, Y., Xiao, F., Xia, Z., and Rao, J. (2007). Quantum dot/bioluminescence resonance energy transfer based highly sensitive detection of proteases. Angew. Chem. Int. Ed. *46*, 4346–4349.

50. Dennis, A.M., Sotto, D.C., Mei, B.C., Medintz, I.L., Mattoussi, H., and Bao, G. (2010). Surface ligand effects on metal-affinity coordination to quantum dots: Implications for nanoprobe self-assembly. Bioconjugate Chem. *21*, 1160–1170.

51. Susumu, K., Medintz, I.L., Delehanty, J.B., Boeneman, K., and Mattoussi, H. (2010). Modification of poly(ethylene glycol)-capped quantum dots with nickel nitrilotriacetic acid and self-assembly with histidine-tagged proteins. J. Phys. Chem. C *114*, 13526–13531.

52. Claussen, J.C., Malanoski, A., Breger, J.C., Oh, E., Walper, S.A., Susumu, K., Goswami, R., Deschamps, J.R., and Medintz, I.L. (2015). Probing the enzymatic activity of alkaline phosphatase within quantum dot bioconjugates. J. Phys. Chem. C *119*, 2208–2221.

53. Brown III, C.W., Oh, E., Hastman, D.A., Walper, S.A., Susumu, K., Stewart, M.H., Deschamps, J.R., and Medintz, I.L. (2015). Kinetic enhancement of the diffusion-limited enzyme beta-galactosidase when displayed with quantum dots. RSC Adv. *5*, 93089–93094.

54. Breger, J.C., Buckhout-White, S., Walper, S.A., Oh, E., Susumu, K., Ancona, M.G., and Medintz, I.L. (2017). Assembling high activity phosphotriesterase composites using hybrid nanoparticle peptide-DNA scaffolded architectures. Nano Futures *1*, 011002.

55. Vranish, J.N., Ancona, M.G., Oh, E., Susumu, K., and Medintz, I.L. (2017). Enhancing coupled enzymatic activity by conjugating one enzyme to a nanoparticle. Nanoscale *9*, 5172–5187.

56. Hondred, J.A., Breger, J.C., Garland, N.T., Oh, E., Susumu, K., Walper, S.A., Medintz, I.L., and Claussen, J.C. (2017). Enhanced enzymatic activity from phosphotriesterase trimer gold nanoparticle bioconjugates for pesticide detection. Analyst *142*, 3261–3271.

57. Malanoski, A.P., Breger, J.C., Brown, C.W., Deschamps, J.R., Susumu, K., Oh, E., Anderson, G.P., Walper, S.A., and Medintz, I.L. (2017). Kinetic enhancement in high-activity enzyme complexes attached to nanoparticles. Nanoscale Horiz. *2*, 241–252.

58. Samanta, A., Breger, J.C., Susumu, K., Oh, E., Walper, S.A., Bassim, N., and Medintz, I.L. (2018). DNA–nanoparticle composites synergistically enhance organophosphate hydrolase enzymatic activity. ACS Appl. Nano Mater. *1*, 3091–3097.

59. Sapsford, K.E., Granek, J., Deschamps, J.R., Boeneman, K., Blanco-Canosa, J.B., Dawson, P.E., Susumu, K., Stewart, M.H., and Medintz, I.L.

(2011). Monitoring botulinum neurotoxin A activity with peptide-functionalized quantum dot resonance energy transfer sensors. ACS Nano *5*, 2687–2699.

60. Sapsford, K.E., Medintz, I.L., Golden, J.P., Deschamps, J.R., Uyeda, H.T., and Mattoussi, H. (2004). Surface-immobilized self-assembled protein-based quantum dot nanoassemblies. Langmuir *20*, 7720–7728.

61. Bachosz, K., Zdarta, J., Bilal, M., Meyer, A.S., and Jesionowski, T. (2023). Enzymatic cofactor regeneration systems: A new perspective on efficiency assessment. Sci. Total Environ. *868*, 161630.

62. Zachos, I., Nowak, C., and Sieber, V. (2019). Biomimetic cofactors and methods for their recycling. Curr. Opin. Chem. Biol. *49*, 59–66.

63. Paul, C.E., and Hollmann, F. (2016). A survey of synthetic nicotinamide cofactors in enzymatic processes. Appl. Microbiol. Biotechnol. *100*, 4773–4778.

64. Oh, E., Liu, R., Nel, A., Gemill, K.B., Bilal, M., Cohen, Y., and Medintz, I.L. (2016). Meta-analysis of cellular toxicity for cadmium-containing quantum dots. Nat. Nanotechnol. *11*, 479–486.

65. Li, H., Shih, W.Y., and Shih, W.-H. (2007). Non-heavy-metal ZnS quantum dots with bright blue photoluminescence by a one-step aqueous synthesis. Nanotechnology *18*, 205604.

STAR★METHODS

KEY RESOURCES TABLE

REAGENT or RESOURCE	SOURCE	IDENTIFIER
Bacterial and virus strains		
E. coli BL21(DE3) strain	New England BioLabs	Cat. #C2527H
Chemicals, peptides, and recombinant proteins		
Adenosine diphosphate disodium salt (ADP)	Sigma Aldrich	Cat. # A2754
Nicotinamide adenine dinucleotide (NADH) disodium salt	Research Products International (RPI)	Cat. # N20100
Phosphoenolpyruvate monopotassium salt (PEP)	Beantown Chemical	Cat. # 129745
Ni^{2+}-nitrilotriacetic acid (Ni-NTA)	Qiagen	Cat. # 30230
isopropyl β-D-1-thiogalactopyranoside (IPTG)	Thermo Fisher Scientific	Cat. # 15529019
HEPES (2-[4-(2-hydroxyethyl)piperazin-1-yl]ethanesulfonic acid)	Sigma Aldrich	Cat #H3375
Imidazole	Fisher Scientific	Cat. # L-13902
Sodium chloride	Fisher Scientific	Cat. # BP358-1
Potassium chloride	Sigma Aldrich	Cat. # P-9541
Sodium phosphate monobasic	Sigma Aldrich	Cat. #S8282
Sodium phosphate dibasic	Fisher Scientific	Cat. #S373
Pyruvic acid sodium salt	VWR	Cat. # 97061-448
Ethylenediaminetetraacetic Acid (EDTA)	EM Science	Cat. # EX0534-1
Magnesium chloride hexahydrate	Fisher Scientific	Cat. # BP214
Sodium hydroxide (NaOH)	Sigma Aldrich	Cat. # 795429
10× tris(hydroxymethyl)aminomethane (Tris)-borate-EDTA (ethylenediaminetetraacetic acid) buffer (10xTBE)	Thermo Fisher Scientific	Cat. # AM9863
low electroendosmosis (EEO) agarose	Sigma Aldrich	Cat. # A6013
Experimental models: Organisms/strains		
E. coli D-lactate dehydrogenase (LDH)		EC 1.1.1.28
E. coli pyruvate kinase A (PykA)		EC 2.7.1.40
E. coli Glucokinase (Glk)		EC 2.7.1.1
E. coli Phosphoglucose isomerase (PGI)		EC 5.3.1.9
E. coli Phosphofructokinase I (PFK)		EC 2.7.1.11
E. coli Fructose-bisphosphate aldolase (FBA)		EC 4.1.2.13
E. coli Triose phosphate isomerase (TPI)		EC 5.3.1.1
E. coli Glyceraldehyde-3-phosphate dehydrogenase (GPD)		EC 1.2.1.12
E. coli Phosphoglycerate kinase (PGK)		EC 2.7.2.3
Other		
TEM Grids: Ultrathin Carbon Film on Lacey Carbon Support Film, 400 mesh, Reference Max H7, Copper	Ted Pella	Product #: 01825

RESOURCE AVAILABILITY

Lead contact

Further information and requests for resources and reagents should be directed to and will be fulfilled by the lead contact, Igor L. Medintz (igor.medintz@nrl.navy.mil).

Materials availability

This study did not generate new unique reagents.

Data and code availability

- The article includes all data generated or analyzed during this study. Original source data for figures in the paper are available upon request to the lead contact.
- This study did not generate novel algorithms or code.
- Any additional information needed to re-analyze the results reported in this paper is available from the lead contact upon request.

EXPERIMENTAL MODEL AND STUDY PARTICIPANT DETAILS

Protein Sequences (given N- to C-terminal for protein monomers).

Pyruvate kinase II (PykA)

MGSSHHHHHHSSGLVPRGSHMSRRLRRTKIVTTLGPATDRDNNLEKVIAAGANVVRMNFSHGSPEDHKMRADKVREIAAKLGRHVAIL
GDLQGPKIRVSTFKEGKVFLNIGDKFLLDANLGKGEGDKEKVGIDYKGLPADVVPGDILLLDDGRVQLKVLEVQGMKVFTEVTVGGPLSN
NKGINKLGGGLSAEALTEKDKADIKTAALIGVDYLAVSFPRCGEDLNYARRLARDAGCDAKIVAKVERAEAVCSQDAMDDIILASDVVMV
ARGDLGVEIGDPELVGIQKALIRRARQLNRAVITATQMMESMITNPMPTRAEVMDVANAVLDGTDAVMLSAETAAGQYPSETVAAMARV
CLGAEKIPSINVSKHRLDVQFDNVEEAIAMSAMYAANHLKGVTAIITMTESGRTALMTSRISSGLPIFAMSRHERTLNLTALYRGVTPVHFD
SANDGVAAASEAVNLLRDKGYLMSGDLVIVTQGDVMSTVGSTNTTRILTVE

Lactate dehydrogenase (LDH)

MGSSHHHHHHSSGLVPRGSHMKLAVYSTKQ
YDKKYLQQVNESFGFELEFFDFLLTEKTAKTANGCEAVCIFVNDDGSRPVLEELKKHGVKYIALRCAGFNNVDLDAAKELGLKVVRVPAY
DPEAVAEHAIGMMMTLNRRIHRAYQRTRDANFSLEGLTGFTMYGKTAGVIGTGKIGVAMLRILKGFGMRLLAFDPYPSAAALELGVEYV
DLPTLFSESDVISLHCPLTPENYHLLNEAAFDQMKNGVMIVNTSRGALIDSQAAIEALKNQKIGSLGMDVYENERDLFFEDKSNDVIQDD
VFRRLSACHNVLFTGHQAFLTAEALTSISQTTLQNLSNLEKGETCPNELV

Enzyme expression and purification generalized protein expression and purification protocol

Plasmid DNA with each gene was transformed into *E. coli*, strain BL21(DE3) for bacterial expression. Single colonies from antibiotic selection plates (LB agar plus 50 μg/mL kanamycin) were inoculated and grown in liquid broth, then combined with sterile glycerol to prepare glycerol stocks which were stored at −80°C. Protein expression and purification generally utilized the following procedure. Starter cultures of 5 mL LB or TB with 50 μg/mL kanamycin were grown overnight at 37°C and shaking at ~180 rpm. The following morning, 500 mL of TB or LB containing 50 μg/mL kanamycin in a 2 L baffled flask was inoculated with a single 5 mL starter culture. Flasks were incubated at 37°C and shaking at 180 rpm for 3–4 h or until mid-log (OD_{600} = 0.6–0.8) was obtained. Then the temperature was lowered to 30°C. Production was initiated through the addition of 0.5 mM isopropyl-β-Dthiogalactopyranoside (IPTG) and shaking was maintained at 180 rpm for 12–16 h. Cell suspensions were transferred to polypropylene, screw-top bottles and centrifuged for 15 min at 4000 x g and 4°C to pellet cells. Pellets were then transferred to −80°C freezers to await further processing (minimum storage of 2 h at this temperature). Cell pellets were thawed on ice then re-suspended in lysis buffer (1/2x phosphate buffered saline, 1 mM EDTA, 1 mg/mL hen egg white lysozyme, 0.1% Triton X-100) and incubated on ice for at least 30 min with periodic mixing. Following incubation on ice, samples were sonicated using a Branson sonifier at 90% amplitude, cycle 0.5, and 60s intervals. A minimum of three cycles were used to ensure cell lysis. Lysates were transferred to either a 50 mL Falcon tube (Fisher Scientific, USA) or a Nalgene 50 mL Oak Ridge style 3119 tubes (Sigma Aldrich, USA) and centrifuged at 4°C and 10,000 × g for 45 min to pellet cell debris. Soluble material was decanted to a clean 50 mL Falcon tube (Fisher Scientific, USA) and placed on ice. A 750 μL aliquot of immobilized metal-affinity chromatography (IMAC) resin (Ni Sepharose High Performance, Sigma Aldrich, USA) was transferred to a microfuge tube then equilibrated in column wash buffer (50 mM phosphate pH 6.0, 300 mM NaCl, 25 mM imidazole) using a batch wash method. Equilibrated resin was added to the soluble protein fraction and the entire suspension was equilibrated through addition of the stock wash buffer solution (prepared at 5x strength) which was diluted to a final ~0.5–1x concentration. The Falcon tubes were transferred to a rotary wheel and incubated overnight at 4°C. Resin was then batch washed in the Falcon tubes using low speed centrifugation (400 x g) and cold column wash buffer. Resin was washed with a minimum 60 bed volumes using this method then transferred to a gravity chromatography column (9 cm Poly-Prep Chromatography Columns, Bio Rad, USA). Captured proteins were eluted with wash buffer containing 300 mM imidazole. Fractions were collected in 0.8 mL aliquots which were stored on ice. Protein-containing fractions were identified (e.g., via measurement of absorbance at 280 nm using a Nanodrop One Microvolume UV-Vis Spectrophotometer (ThermoFisher Scientific, USA); examined for purity via SDS-PAGE on 4–15% gradient Tris-glycine gels (Bio Rad, USA)). Enzyme-containing fractions were pooled and purified either by dialysis against 50 mM phosphate buffer (pH 8.0) or 20 mM phosphate buffer (pH 7.4) or by loading on to BioRad Biologic Fast Protein Liquid Chromatography (FPLC) system with a SEC 650 column. Final enzyme concentration was determined by UV-vis measurement of their absorbance using their predicted extinction coefficient. Enzymes samples were supplemented with 20–30% glycerol prior to aliquoting into 0.5 mL microfuge tubes for snap freezing in a dry ice-methanol bath and final storage at −80°C. For assays, individual tubes were removed from storage, thawed, used, and any remaining enzyme discarded.

METHOD DETAILS

Quantum dots

CdSe/CdS/ZnS core/shell/shell QDs were synthesized as previously described. Briefly, QDs were cap exchanged with the zwitterionic dihydrolipoic acid- (DHLA) based Compact Ligand CL4. This ligand provides for long-term QD colloidal stability in buffer and challenging environments such as cells and tissues while still allowing polyhistidine metal-affinity coordination of enzymes to the QDs surface. QD size was confirmed with transmission electron microscopy (TEM) analysis as previously described.

Cap exchange of CdSe/ZnS NPLs with CL4

The disulfide, methyl ester form of CL4 (126 mg, 0.3 mmol) was dissolved in ethanol (1 mL) and DI water (0.5 mL) and stirred with LiOH (16 mg, 0.67 mmol).[42] After 1 h, the solution was adjusted to pH 7–8 by slowly adding 4M HCl dropwise. Next, $NaBH_4$ (25 mg, 0.66 mmol) was added and the mixture was stirred for 1 h. After the solution turned colorless, 4M HCl was added dropwise to adjust the pH to 7–8. Separately, a portion of CdSe/ZnS NPLs (2 nmol) in toluene were precipitated with minimal isopropanol and centrifuged at 3500 rpm for 5 min. The supernatant was discarded and the CdSe/ZnS NPLs were dissolved in 1 mL of chloroform and added to the activated ligand mixture with vigorous stirring. Small portions of chloroform and DI water were added until a biphasic mixture was achieved. The mixture was rapidly stirred until the NPLs were transferred to the aqueous phase (2–24 h). The organic phase was discarded and the aqueous phase was washed with $CHCl_3$ (3 × 1 mL). The aqueous phase was filtered through a 0.45 μm hydrophilic membrane filter (Millipore) and washed with DI water (2-3 × 1.5 mL) using a centrifugal filtration device (Millipore, MW cutoff 100 kDa). The aqueous CL4-capped NPLs were stored at 4°C in the dark until further use. The final NPL material had an emission at ~585 nm (585 NPL).

Physicochemical characterization

Agarose gel mobility assays

Agarose gel separation of 525 QDs assembled with increasing ratios of each enzyme utilized in this study were undertaken to confirm that each enzyme did indeed have the ability to coordinate to the surface of the ZnS-overcoated QDs when assembled as nanoconjugates. Low electroendosmosis (EEO) agarose gels were utilized with percentages as indicated in each image set. The percentage agarose in each gel was varied as needed to obtain separation. Gels were imaged on a Biorad Gel Doc XR System. This type of assay is now a common method for confirming that a protein or other molecule such as DNA has indeed assembled to a QD. Tris/Borate/EDTA (TBE) buffer is 89 mM Tris, 89 mM boric acid, 2 mM EDTA pH 8.3 and was used as is unless otherwise indicated. In some cases, the pH was changed to enhance QD-protein separation. Images were collected at every 5 min or as indicated during separation to show the evolution of mobility differences with time.

TEM methods

TEM imaging of the QDs and QD-enzyme conjugates was performed as described in previous publications.[20] Briefly, the TEM grids used in this study (Ultrathin Carbon Film on Lacey Carbon Support Film, 400 mesh, Reference Max H7, Copper from Ted Pella) were plasma-cleaned prior to use. The plasma-cleaned grid was placed face up on clean filter paper and a 10 μL drop of 0.1 mg/mL poly-L-lysine solutions was dropped onto the center of the grid and left to dry for 1 min. The 10 μL of water was dropped onto the center of the grid and left to dry for 1 min. Then 5 μL of sample was dropped onto the center of the grid and left to dry for 10 min. Finally, another 10 μL of water was dropped onto the center of the grid to remove and excess salt and after being left to dry for another 10 min the TEM grid was ready for imaging.

Kinetic assays

Pyruvate kinase (PykA) Assay

NADH consumption was monitored as a function of time at 340 nm to determine the apparent kinetic parameters of PykA as displayed on the different nanoparticles or free in solution at the same concentration. Stock solutions were prepared in 120 mM HEPES (pH 8) with 1 mM EDTA and 10 mM KCl and consisted of: a 500 nM PykA stock, a 5000 nM LDH stock, and a 500 nM nanoparticle stock. Additional stock solutions were prepared in 120 mM HEPES (pH 8) and consisted of: a 100 mM PEP stock, a 100 mM ADP stock, an 800 mM $MgCl_2x6H_2O$ stock, a 50 mM NADH stock. The pH of the PEP stock solution was monitored and adjusted as necessary from a 5 M NaOH stock prior to mixing to ensure a pH of 8 was maintained. Then solutions of 5 nM PykA were mixed with increasing concentrations of nanoparticle and stored in the dark at 4°C for 3 h to ensure self-assembly of the PykA-nanoparticle bioconjugate. The blocking peptide was added to the nanoparticle containing solutions in 100-fold excess of the nanoparticle concentration to block any remaining open sites on the nanoparticle and stored in the dark at 4°C for 3 h. Then 500 nM LDH was added to each solution. The assays were performed in 40 μL total volume with 20 μL of the enzyme-nanoparticle solution, 10 μL of solution containing ADP (4 mM) and NADH (1 mM), and 10 μL of solution containing variable PEP (0.4–40 mM) and $MgCl_2x6H_2O$ (20 mM). The final concentrations for the assays were 2.5 nM PykA, 250 nM LDH, varied nanoparticle concentration (0–5 nM), variable PEP (0.1–10 mM), 1 mM ADP, 10 mM MgCl2, 0.25 mM NADH, 0.5 mM EDTA and 10 mM KCl. Reactions were initiated by the simultaneous addition of $MgCl_2$ and PEP. Assays were carried out in a 384-well microtiter white transparent bottom plate. The absorbance at 340 nm was followed on a Tecan Spark plate reader utilizing a kinetic program that consisted of shaking the plate for 3 s prior to taking a reading every 26 s. Absorbance values were converted to concentration values utilizing the molar extinction coefficient of NADH. The rate of NADH consumption was directly converted into the rate of NAD^+ formation. The linear portions of the progress curves

were used to calculate the initial rates for each substrate concentration. These were fitted to the Michaelis–Menten equation using either Excel's solver module or Sigma Plot's enzyme module. All activity measurements were performed on at least three independently-assembled replicates.

Lactate dehydrogenase (LDH) Assay

NADH consumption was monitored as a function of time at 340 nm to determine the apparent kinetic parameters of LDH as displayed on the different nanoparticles or free in solution at the same concentration. Stock solutions were prepared in 120 mM HEPES (pH 8) with 1 mM EDTA and 10 mM KCl and consisted of: a 500 nM LDH stock, a 5000 nM PykA stock, and a 500 nM nanoparticle stock. Additional stock solutions were prepared in 120 mM HEPES (pH 8) and consisted of: a 100 mM PEP stock, a 100 mM ADP stock, an 800 mM $MgCl_2x6H_2O$ stock, a 50 mM NADH stock. The pH of the PEP stock solution was monitored and adjusted as necessary from a 5 M NaOH stock prior to mixing to ensure a pH of 8 was maintained. Then solutions of 5 nM LDH were mixed with increasing concentrations of nanoparticle and stored in the dark at 4°C for 3 h to ensure self-assembly of the LDH-nanoparticle bioconjugate. The blocking peptide was added to the nanoparticle containing solutions in 100-fold excess of the nanoparticle concentration to block any remaining open sites on the nanoparticle and stored in the dark at 4°C for 3 h. Then 500 nM PykA was added to each solution. The assays were performed in 40 μL total volume with 20 μL of the enzyme-nanoparticle solution, 10 μL of solution containing ADP (4 mM) and NADH (1 mM), and 10 μL of solution containing variable PEP (0.4–40 mM) and $MgCl_2x6H_2O$ (20 mM). The final concentrations for the assays were 2.5 nM LDH, 250 nM PykA, varied nanoparticle concentration (0–5 nM), variable PEP (0.1–10 mM), 1 mM ADP, 10 mM $MgCl_2$, 0.25 mM NADH, 0.5 mM EDTA and 10 mM KCl. Reactions were initiated by the simultaneous addition of $MgCl_2$ and PEP. Assays were carried out in a 384-well microtiter white transparent bottom plate. The absorbance at 340 nm was followed on a Tecan Spark plate reader utilizing a kinetic program that consisted of shaking the plate for 3 s prior to taking a reading every 26 s. Absorbance values were converted to concentration values utilizing the molar extinction coefficient of NADH. The rate of NADH consumption was directly converted into the rate of NAD^+ formation. The linear portions of the progress curves were used to calculate the initial rates for each substrate concentration. These were fitted to the Michaelis–Menten equation using either Excel's solver module or Sigma Plot's enzyme module. All activity measurements were performed on at least three independently-assembled replicates.

Coupled enzyme Assay procedures

In general, the concentrations of nanoparticles were varied as indicated in figure legends and tables. For all coupled assay procedures, the concentration of PykA was 20 nM and the concentration of LDH was 20 nM. Unless stated otherwise enzyme-nanoparticle assembly was carried out by mixing the enzyme(s) solution in buffer and then subsequently adding the individual nanoparticle. Where mixed nanoparticle assays were carried out, the two types of nanoparticles were mixed together before being added to the PykA and LDH mixture. Nanoparticle(s)-QD solution were stored in the dark at 4°C for 3 h prior to running the assay to ensure adequate time for assembly to occur. Stock solutions of enzyme and nanoparticle were made by diluting aliquots of each solution in buffer (120 mM HEPES (pH 8), 1 mM EDTA, and 20 mM KCl). Substrate stock solutions were prepared in an analogous manner as described for the individual enzyme assays above. The final substrate concentrations for the assays were variable PEP (0.1–10 mM), 1 mM ADP, 10 mM $MgCl_2$, 0.25 mM NADH, 0.5 mM EDTA and 10 mM KCl. Reactions were initiated by the simultaneous addition of $MgCl_2$ and PEP. All coupled assays where NADH consumption was monitored were carried out in a 384-well plate in a Tecan Spark Microplate reader.

QUANTIFICATION AND STATISTICAL ANALYSIS

For quantification and analysis of all kinetic data described herein, the linear portions of the progress curves were used to calculate the initial rates for each substrate concentration. These were fitted to the Michaelis–Menten equation using either Excel's solver module or Sigma Plot's enzyme module. All activity measurements were performed on at least three individual replicates.

Cell Reports Methods, Volume *4*

Supplemental information

Optimizing the conversion of phosphoenolpyruvate to lactate by enzymatic channeling with mixed nanoparticle display

Shelby L. Hooe, Christopher M. Green, Kimihiro Susumu, Michael H. Stewart, Joyce C. Breger, and Igor L. Medintz

Supporting Information

Optimizing the Conversion of Phosphoenolpyruvate to Lactate by Enzymatic Channeling with Mixed Nanoparticle Display

Shelby L. Hooe,[a] Christopher M. Green,[a] Kimihiro Susumu,[b] Michael H. Stewart,[b]

Joyce C. Breger,[a] and Igor L. Medintz[a*]

[a]Center for Bio/Molecular Science and Engineering Code 6900
[b]Optical Sciences Division, Code 5611
U.S. Naval Research Laboratory
Washington, D.C. 20375, USA

[*]Email: igor.medintz@nrl.navy.mil

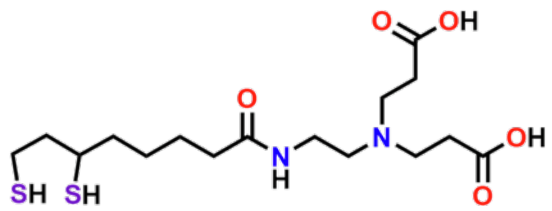

Figure S1. Structure of CL4 ligand shown with the thiols open as the dithiolane (related to Figure 1C). This ligand on the QD surface enables colloidal stability of enzyme-QD mixtures.

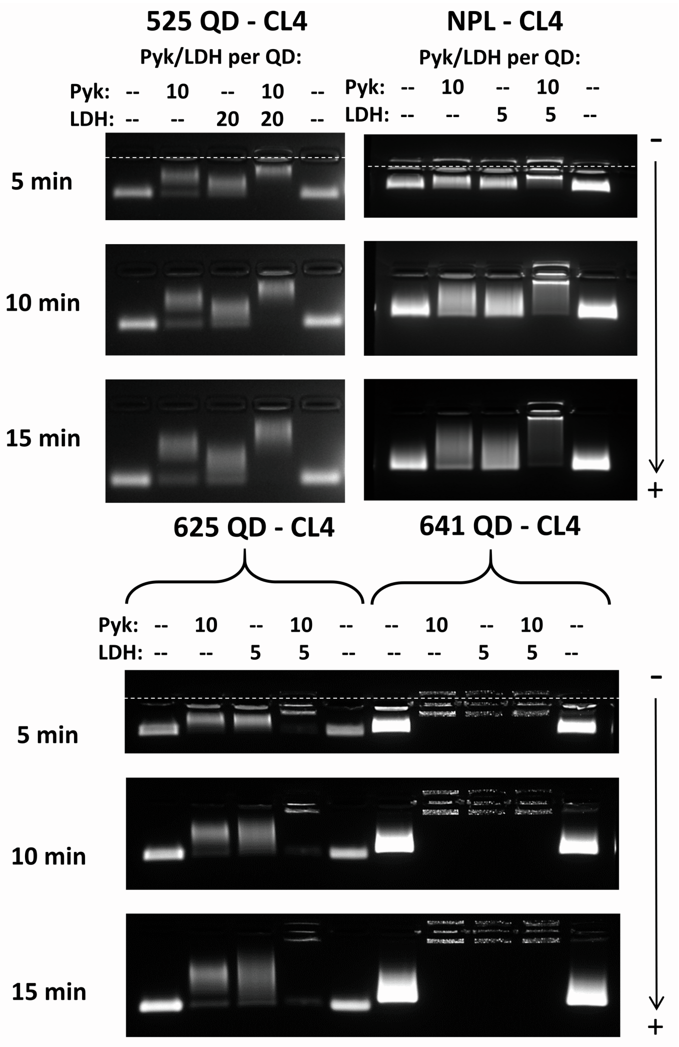

Figure S2. Characterization of QD enzyme clusters. This data supports the gel data shown in Figure 2A. ***Top*** - representative agarose gel mobility assay confirming PykA and LDH assembly to the 523 nm emitting QDs and 585 nm NPLs capped with CL4 ligand. 2.5 picomoles of nanoparticle/well were assembled with the indicated ratio of PykA and/or LDH enzyme and then separated in a 1.5% and 1.0% agarose gel for the 523 nm QD and 585 nm NPLs, respectively, supplemented with 1×TBE buffer. The degree of nanoparticle mobility shifting correlates to the increased ratio of enzyme displayed on their surface. ***Bottom*** - representative agarose gel mobility assay confirming PykA and LDH assembly to the 625 nm and 641 nm emitting QDs capped with CL4 ligand. 2.5 picomoles of QD/well were assembled with the indicated ratio of PykA and/or LDH enzyme and then separated in a 1.0% agarose gel for the supplemented with 1×TBE buffer. The degree of nanoparticle mobility shifting correlates to the increased ratio of enzyme displayed on their surface. The degree of QD mobility is directly correlated to the ratio of different enzymes displayed on their surface. Ratios of enzyme used for gel assays do not correlate to the empirically estimated number of each enzymes that fit around the QD but are utilized to improve differences in mobility. Excess enzyme is used since the sieving action of the gel can remove loosely associated enzyme on the QDs. The location of the wells are indicated by the white dashed line in the 5 minute images.

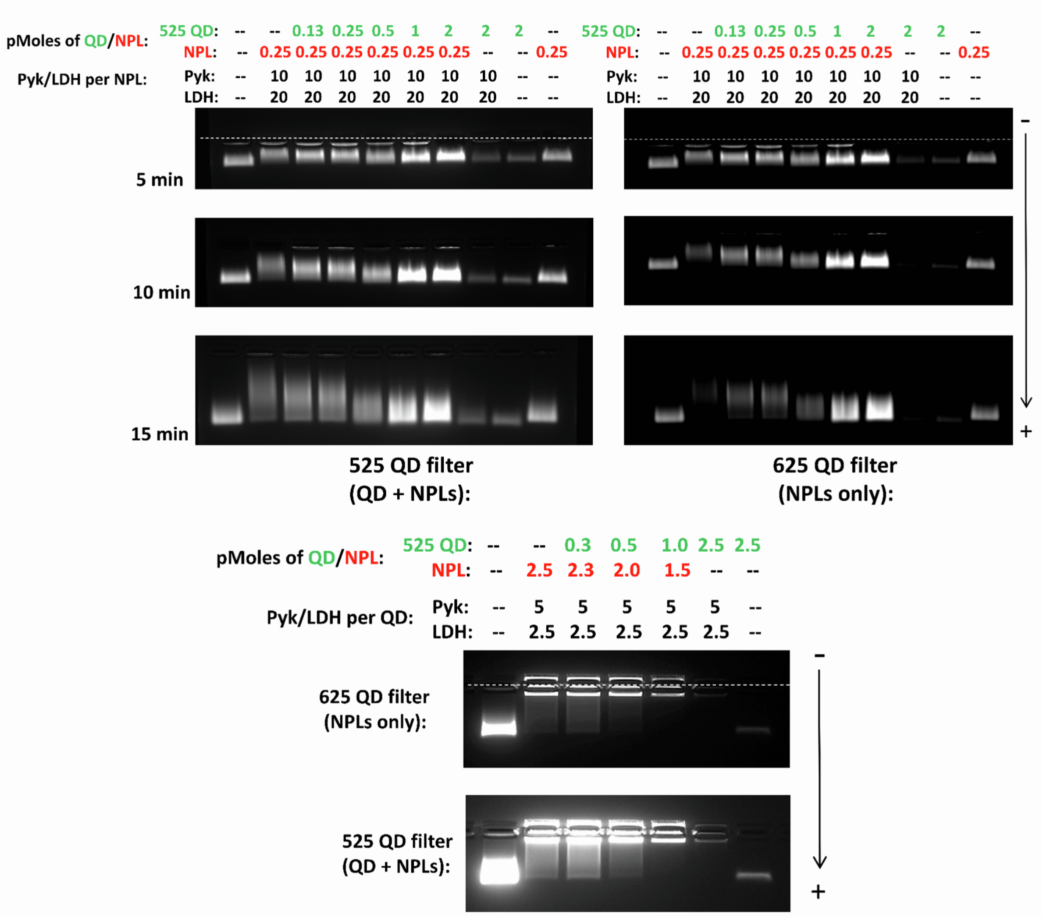

Figure S3. Agarose gels of mixed NP assembly. This data supports the gel data shown in Figure 2A. (Top) Agarose gel mobility assay confirming assembly of the PykA→LDH 2 enzyme cascade to 525 nm emitting QDs and NPLs capped with CL4 ligand. Gel in 2.0% agarose 1×TBE buffer. Enzymes added to QDs and/ NPLs in their ratios shown. These ratios are different from those used in the catalytic assays and were arrived at empirically to reveal changes in mobility during electrophoresis with each sequential nanoparticle addition. (Bottom) Agarose gel mobility assay confirming assembly of the PykA→LDH 2 enzyme cascade to 525 nm emitting QDs and NPLs capped with CL4 ligand. Gel in 2.0% agarose 1×TBE buffer. Enzymes added to QDs and/or NPLs in their ratios shown. These ratios are different from those used in the catalytic assays and were arrived at empirically to reveal changes in mobility during electrophoresis with each sequential nanoparticle addition. White dashed line indicates the location of the wells where samples were loaded.

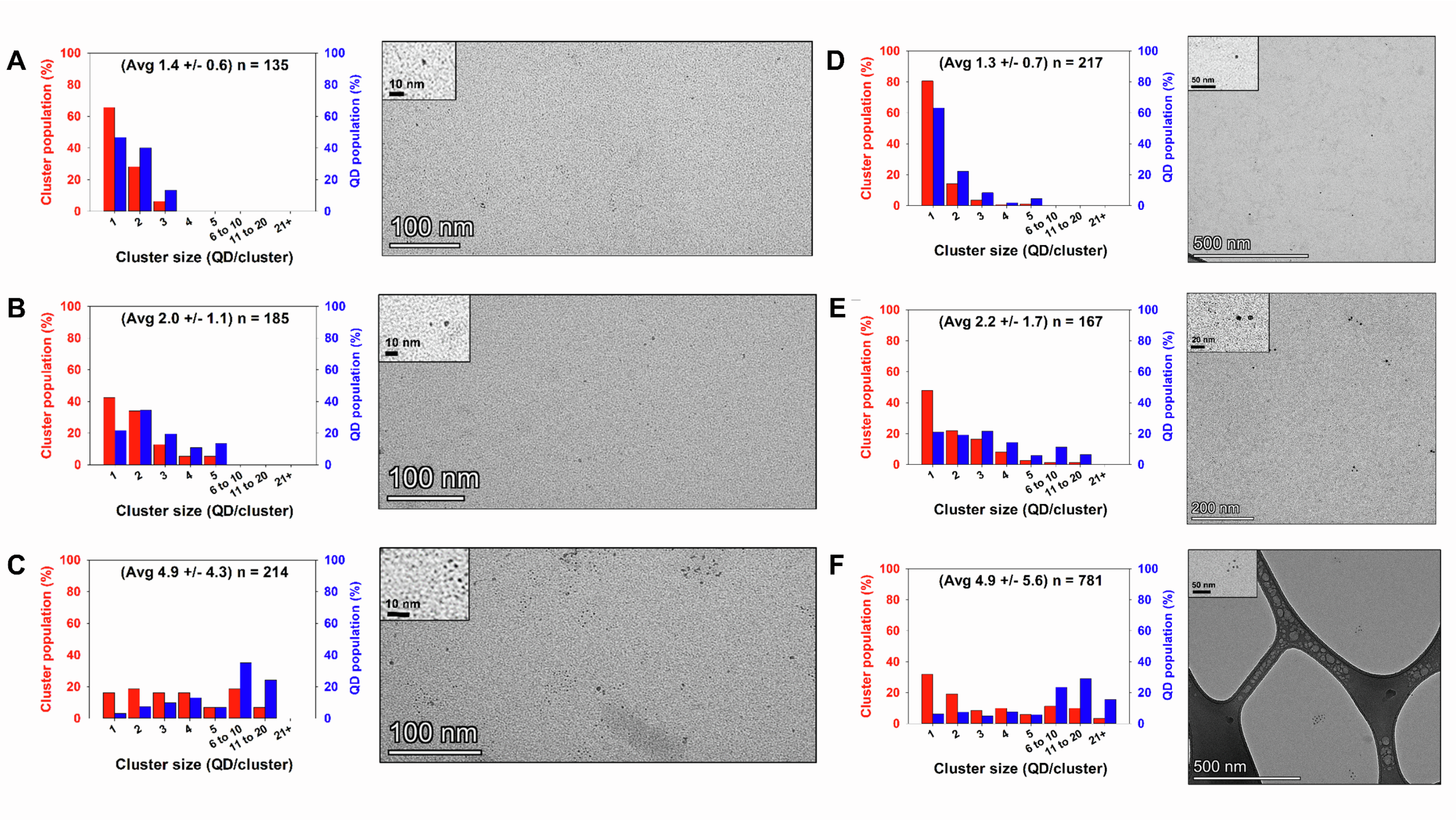

Figure S4. TEM characterization of QD-enzyme clusters with the 525 and 625 QDs. This data supports the TEM data shown in Figure 1C. Representative TEMs of 525 QDs assembled with 40 nM LDH and 20 nM PykA with 0.5 nM **(A)**, 1 nM **(B)**, and 2 nM **(C)** 525 QD. Average cluster size is given above the micrograph along with the number of QDs counted. Corresponding bar plots for each sample below showing the distribution of cluster sizes present (red) and number of NPs per cluster size (blue). TEM characterization of QD-enzyme clusters with the 625 QDs. Representative TEMs of 625 QDs assembled with 40 nM LDH and 20 nM PykA with 0.5 nM **(D)**, 1 nM **(E)**, and 2 nM **(F)** 625 QD. Average cluster size is given in the plots along with the number of QDs counted. Corresponding bar plots for each sample below showing the distribution of cluster sizes present (red) and number of NPs per cluster size (blue).

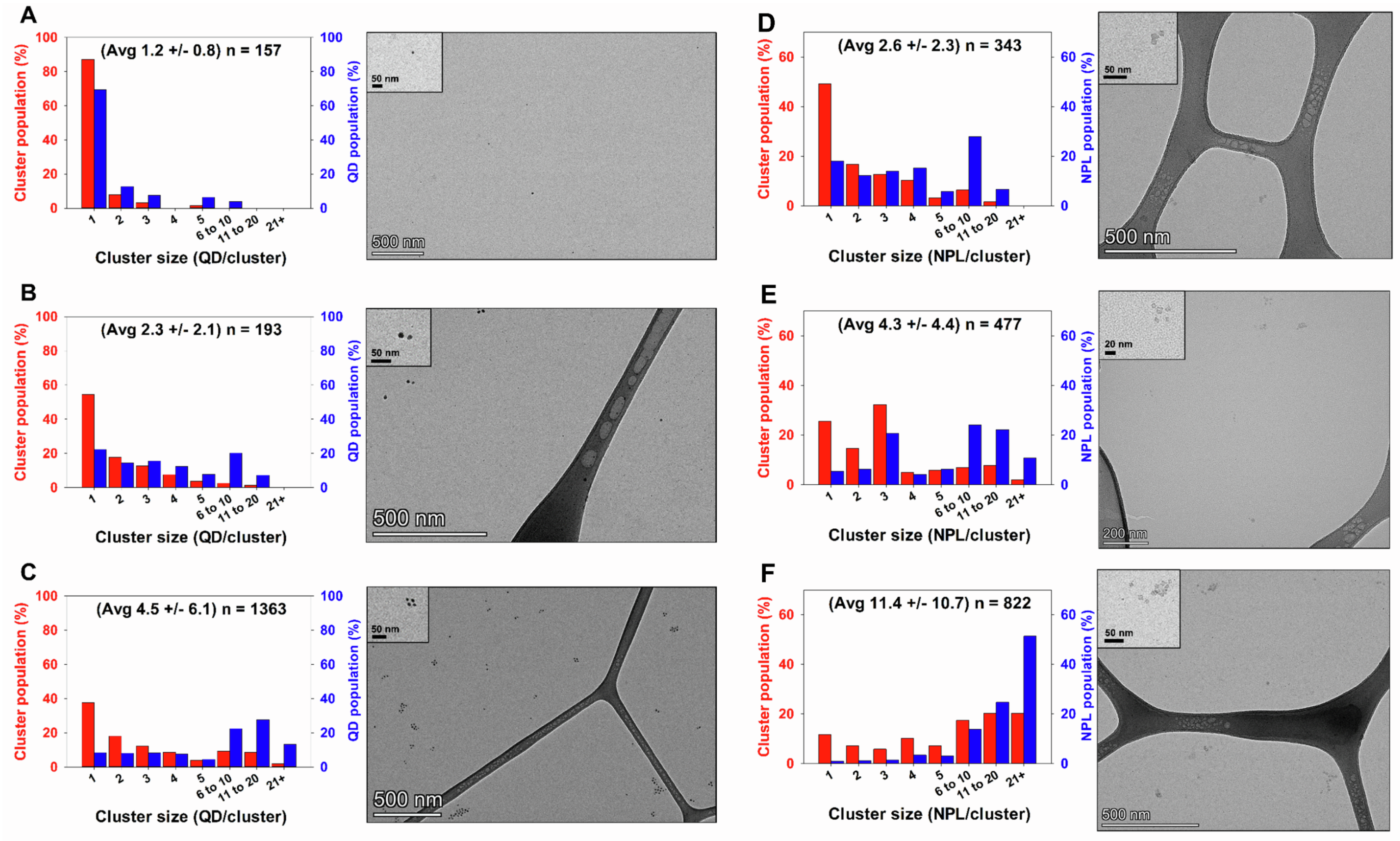

Figure S5. TEM characterization of QD-enzyme clusters with the 641 QDs and NPLs. This data supports the TEM data shown in Figure 1C. Representative TEMs of 641 QDs assembled with 40 nM LDH and 20 nM PykA with 0.5 nM **(A)**, 1 nM **(B)**, and 2 nM **(C)** 641 QD. Average cluster size is given above the micrograph along with the number of QDs counted. Corresponding bar plots for each sample below showing the distribution of cluster sizes present (red) and number of NPs per cluster size (blue). TEM characterization of QD-enzyme clusters with the NPLs. Representative TEMs of NPLs assembled with 40 nM LDH and 20 nM PykA with 0.25 nM **(D)**, 0.5 nM **(E)**, and 1 nM **(F)** NPL. Average cluster size is given in the plots along with the number of NPLs counted. Corresponding bar plots for each sample below showing the distribution of cluster sizes present (red) and number of NPLs per cluster size (blue).

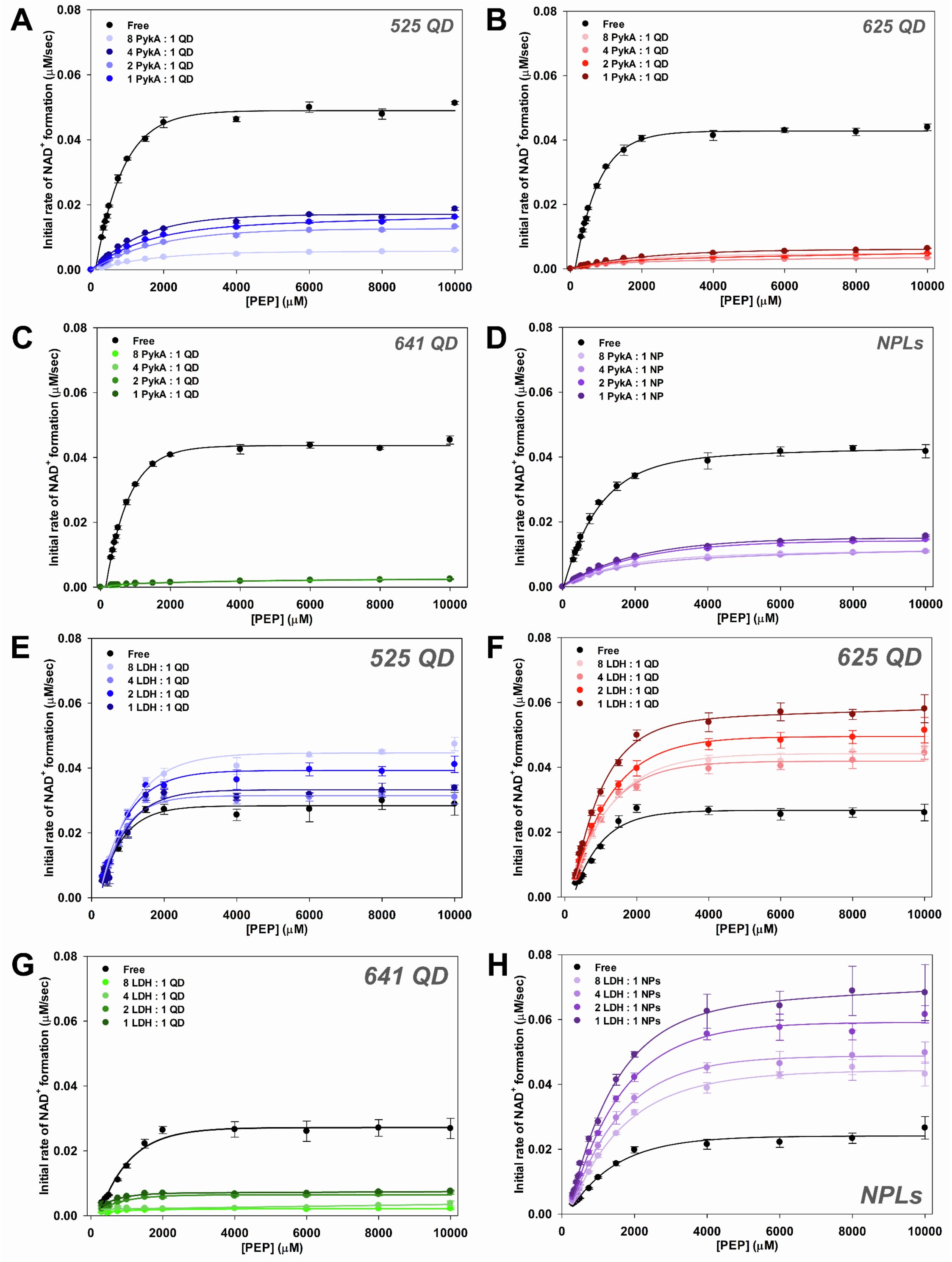
A
525 QD
Free
8 PykA : 1 QD
4 PykA : 1 QD
2 PykA : 1 QD
1 PykA : 1 QD
Initial rate of NAD+ formation (μM/sec)
[PEP] (μM)
B
625 QD
C
641 QD
D
NPLs
8 PykA : 1 NP
4 PykA : 1 NP
2 PykA : 1 NP
1 PykA : 1 NP
E
525 QD
8 LDH : 1 QD
4 LDH : 1 QD
2 LDH : 1 QD
1 LDH : 1 QD
F
625 QD
G
641 QD
H
NPLs
8 LDH : 1 NPs
4 LDH : 1 NPs
2 LDH : 1 NPs
1 LDH : 1 NPs

Figure S6.

Figure S6. MM data for LDH and PykA across the different NP materials. This data supports the kinetic data shown in Figure 3 and Figure 4. (A) MM plots showing initial rates of NAD^+ conversion for free PykA and as assembled to increasing amounts of 525 QD used in the self-assembly *versus* increasing concentrations of PEP. (B) MM plots showing initial rates of NAD^+ conversion for free PykA and as assembled to increasing amounts of 625 QD used in the self-assembly *versus* increasing concentrations of PEP. (C) MM plots showing initial rates of NAD^+ conversion for free PykA and as assembled to increasing amounts of 641 QD used in the self-assembly *versus* increasing concentrations of PEP. (D) MM plots showing initial rates of NAD^+ conversion for free PykA and as assembled to increasing amounts of NPL used in the self-assembly *versus* increasing concentrations of PEP. Reaction conditions for panels A-D include variable PEP, 5 mM $MgCl_2 \times 6H_2O$, 1 mM ADP, 0.25 mM NADH, 2.5 nM PykA, 250 nM LDH, 10 mM KCl, and 0.5 mM EDTA in 120 mM HEPES at pH 8 and 30° C. (E) MM plots showing initial rates of NAD^+ conversion for free LDH and as assembled to increasing amounts of 525 QD used in the self-assembly *versus* increasing concentrations of PEP. (F) MM plots showing initial rates of NAD^+ conversion for free LDH and as assembled to increasing amounts of 625 QD used in the self-assembly *versus* increasing concentrations of PEP. (G) MM plots showing initial rates of NAD^+ conversion for free LDH and as assembled to increasing amounts of 641 QD used in the self-assembly *versus* increasing concentrations of PEP. (H) MM plots showing initial rates of NAD^+ conversion for free LDH and as assembled to increasing amounts of NPL used in the self-assembly *versus* increasing concentrations of PEP. Reaction conditions for panels E-H include variable PEP, 5 mM $MgCl_2 \times 6H_2O$, 1 mM ADP, 0.25 mM NADH, 2.5 nM LDH, 250 nM PykA, 10 mM KCl, and 0.5 mM EDTA in 120 mM HEPES at pH 8 and 30° C.

Table S1. Estimated enzymatic kinetic parameters for PykA when free in solution and as assembled on QDs and NPLs. This data supports the kinetic data shown in Figure 3.

Enzyme: Ratio per QD	V_{Max} ($nM \times s^{-1}$)	k_{cat} (sec^{-1})	K_M (mM)	k_{cat} / K_M ($mM^{-1} \times s^{-1}$)
**PykA:[a] 0**	63.0 ± 1	25.0 ± 0.1	1.3 ± 0.1	$2.0 \times 10^{-5} \pm 2 \times 10^{-6}$
**525 QDs**				
**1**	17.0 ± 1	6.8 ± 0.1	1.3 ± 0.1	$5.3 \times 10^{-6} \pm 5 \times 10^{-7}$
**2**	14.0 ± 1	5.7 ± 0.1	1.4 ± 0.1	$4.1 \times 10^{-6} \pm 4 \times 10^{-7}$
**4**	21.0 ± 1	8.5 ± 0.1	1.6 ± 0.1	$5.3 \times 10^{-6} \pm 3 \times 10^{-8}$
**8**	6.8 ± 0.2	2.7 ± 0.1	1.7 ± 0.2	$1.6 \times 10^{-6} \pm 2 \times 10^{-7}$
**625 QDs**				
**1**	6.9 ± 0.2	2.7 ± 0.1	1.6 ± 0.1	$1.7 \times 10^{-6} \pm 8 \times 10^{-8}$
**2**	5.4 ± 0.2	2.2 ± 0.1	2.0 ± 0.2	$1.1 \times 10^{-6} \pm 1 \times 10^{-7}$
**4**	3.9 ± 0.1	1.6 ± 0.1	1.8 ± 0.3	$8.8 \times 10^{-7} \pm 2 \times 10^{-7}$
**8**	5.7 ± 0.2	2.3 ± 0.1	1.8 ± 0.2	$1.3 \times 10^{-6} \pm 1 \times 10^{-7}$
**641 QDs**				
**1**	2.7 ± 0.2	1.1 ± 0.1	1.4 ± 0.1	$7.7 \times 10^{-7} \pm 9 \times 10^{-8}$
**2**	2.6 ± 0.1	1.1 ± 0.1	1.5 ± 0.1	$7.0 \times 10^{-7} \pm 2 \times 10^{-8}$
**4**	3.0 ± 0.1	1.2 ± 0.1	1.8 ± 0.2	$6.6 \times 10^{-7} \pm 9 \times 10^{-8}$
**8**	2.6 ± 0.1	1.0 ± 0.1	1.5 ± 0.1	$6.9 \times 10^{-7} \pm 6 \times 10^{-8}$
**NPLs**				
**1**	17.0 ± 1	6.8 ± 0.1	1.5 ± 0.1	$4.5 \times 10^{-6} \pm 4 \times 10^{-7}$
**2**	16.0 ± 1	6.4 ± 0.1	1.5 ± 0.2	$4.2 \times 10^{-6} \pm 5 \times 10^{-7}$
**4**	12.0 ± 1	5.0 ± 0.1	1.7 ± 0.2	$2.9 \times 10^{-6} \pm 4 \times 10^{-7}$
**8**	12.0 ± 1	5.0 ± 0.1	1.5 ± 0.1	$3.2 \times 10^{-6} \pm 2 \times 10^{-7}$

Notes. Final enzyme concentration: [a]PykA = 2.5 nM. All kinetic values are qualified as apparent. Ratio of 0 = free enzyme in solution, no QD present.

Table S2. Estimated enzymatic kinetic parameters for LDH when free in solution and as assembled on QDs and NPLs. This data supports the kinetic data shown in Figure 3.

Enzyme: Ratio per QD	V_{Max} (nM × s^{-1})	k_{cat} (sec^{-1})	K_M (mM)	k_{cat} / K_M (mM^{-1} × s^{-1})
**LDH:[a] 0**	33.0 ± 3	13.3 ± 0.2	0.9 ± 0.4	$1.5 \times 10^{-5} \pm 7 \times 10^{-6}$
**525 QDs**				
**1**	40.0 ± 6	15.9 ± 0.4	1.1 ± 0.2	$1.5 \times 10^{-5} \pm 5 \times 10^{-7}$
**2**	45.0 ± 1	17.9 ± 0.1	0.9 ± 0.4	$2.0 \times 10^{-5} \pm 7 \times 10^{-6}$
**4**	40.0 ± 1	15.5 ± 0.1	1.1 ± 0.3	$1.4 \times 10^{-5} \pm 4 \times 10^{-6}$
**8**	57.0 ± 1	22.7 ± 0.1	1.5 ± 0.4	$1.5 \times 10^{-5} \pm 3 \times 10^{-6}$
**625 QDs**				
**1**	69.0 ± 1	27.6 ± 0.2	1.3 ± 0.3	$2.1 \times 10^{-5} \pm 5 \times 10^{-6}$
**2**	58.0 ± 3	23.1 ± 0.2	1.0 ± 0.2	$2.3 \times 10^{-5} \pm 4 \times 10^{-6}$
**4**	49.0 ± 1	19.5 ± 0.1	1.1 ± 0.3	$1.8 \times 10^{-5} \pm 5 \times 10^{-6}$
**8**	52.0 ± 1	20.7 ± 0.1	1.2 ± 0.3	$1.7 \times 10^{-5} \pm 5 \times 10^{-6}$
**641 QDs**				
**1**	8.3 ± 0.1	3.32 ± 0.1	0.6 ± 0.2	$6.0 \times 10^{-6} \pm 2 \times 10^{-6}$
**2**	7.7 ± 0.3	3.07 ± 0.1	0.8 ± 0.1	$3.8 \times 10^{-6} \pm 7 \times 10^{-7}$
**4**	3.6 ± 0.5	1.45 ± 0.1	0.7 ± 0.3	$2.1 \times 10^{-6} \pm 1 \times 10^{-6}$
**8**	2.6 ± 0.3	1.04 ± 0.1	0.8 ± 0.1	$1.3 \times 10^{-6} \pm 3 \times 10^{-7}$
**NPLs**				
**1**	81.0 ± 9	32.3 ± 0.6	1.6 ± 0.3	$2.0 \times 10^{-5} \pm 6 \times 10^{-6}$
**2**	71.0 ± 4	28.6 ± 0.3	0.2 ± 0.2	$1.7 \times 10^{-5} \pm 2 \times 10^{-6}$
**4**	59.0 ± 5	23.6 ± 0.3	1.7 ± 0.4	$1.4 \times 10^{-5} \pm 4 \times 10^{-6}$
**8**	52.0 ± 2	20.9 ± 0.1	1.7 ± 0.3	$1.2 \times 10^{-5} \pm 3 \times 10^{-6}$

Notes. Final enzyme concentration: [a]LDH = 2.5 nM. All kinetic values are qualified as apparent. Ratio of 0 = free enzyme in solution, no QD present.

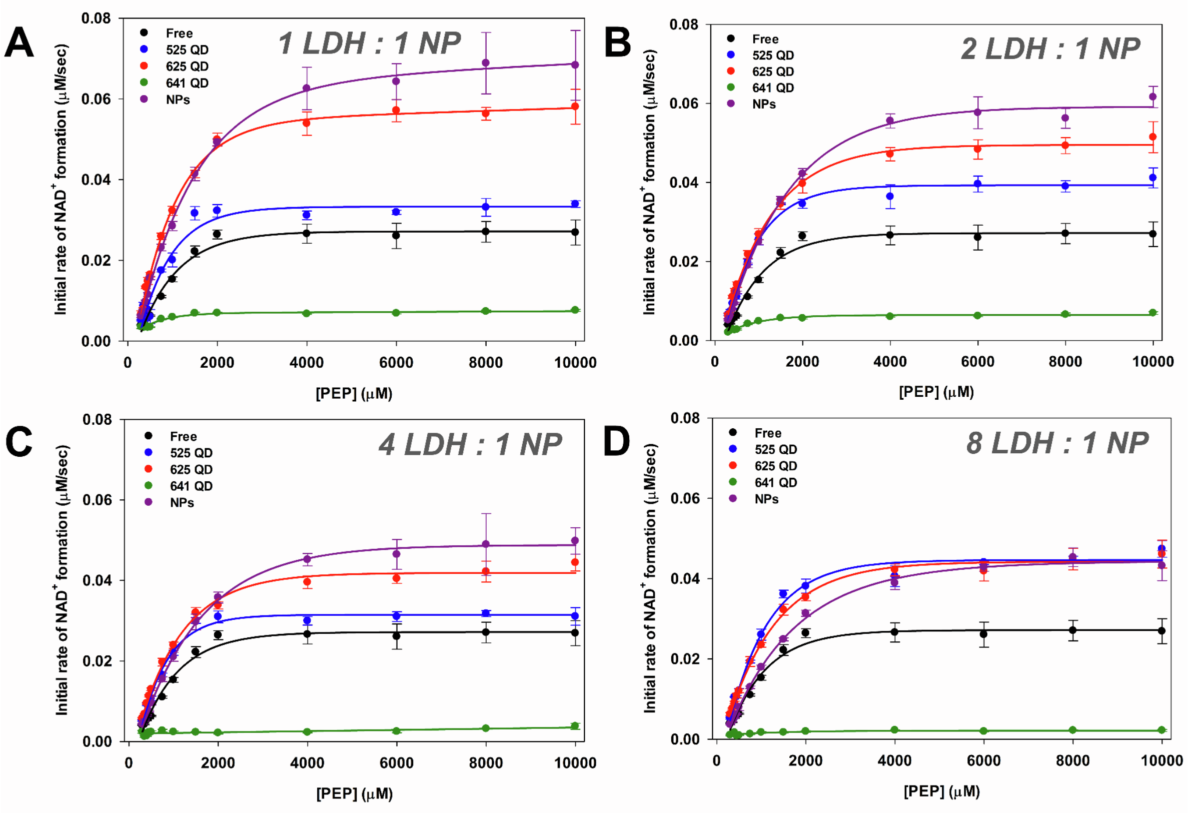

Figure S7. MM plots showing initial rates of NAD^+ conversion for free LDH and as assembled across different nanoparticles used in the self-assembly *versus* increasing concentrations of PEP. This data supports the kinetic data shown in Figure 3. Data collected at the LDH to NPL ratios of 1:1 (A), 2:1 (B), 4:1 (C), and 8:1 (D). Reaction conditions include variable PEP, 5 mM $MgCl_2 \times 6H_2O$, 1 mM ADP, 0.25 mM NADH, 2.5 nM LDH, 250 nM PykA, 10 mM KCl, and 0.5 mM EDTA in 120 mM HEPES at pH 8 and 30° C.

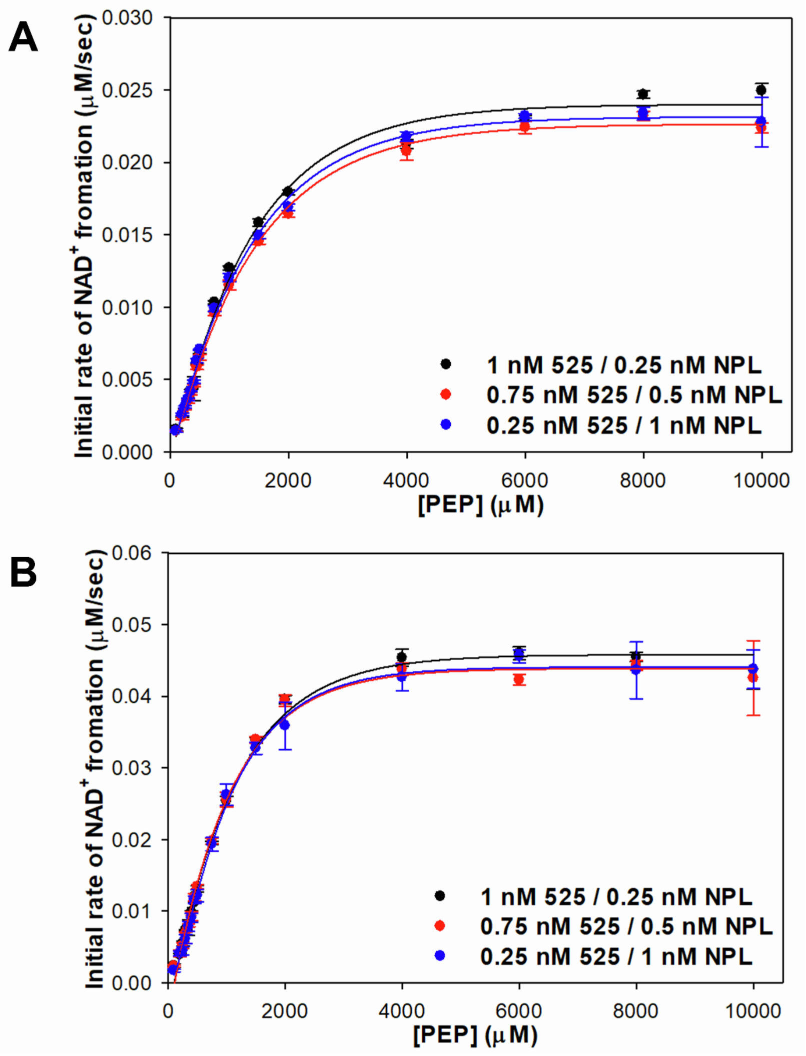

Figure S8. Mixed NP assembly MM data for LDH and PykA. This data supports the kinetic data shown in Figure 5. (A) MM plots showing initial rates of NAD^+ conversion for PykA as assembled across mixed nanoparticles used in the self-assembly *versus* increasing concentrations of PEP. Reaction conditions include variable PEP, 5 mM $MgCl_2 \times 6H_2O$, 1 mM ADP, 0.25 mM NADH, 250 nM LDH, 2.5 nM PykA, 10 mM KCl, and 0.5 mM EDTA in 120 mM HEPES at pH 8 and 30° C. (B) MM plots showing initial rates of NAD^+ conversion for LDH as assembled across mixed nanoparticles used in the self-assembly *versus* increasing concentrations of PEP. Reaction conditions include variable PEP, 5 mM $MgCl_2 \times 6H_2O$, 1 mM ADP, 0.25 mM NADH, 2.5 nM LDH, 250 nM PykA, 10 mM KCl, and 0.5 mM EDTA in 120 mM HEPES at pH 8 and 30° C.

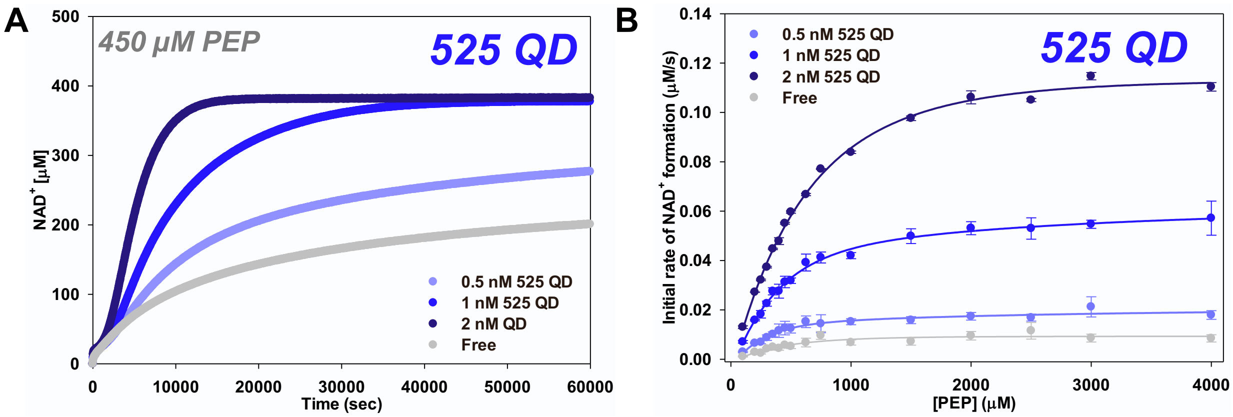

Figure S9. Kinetic enhancement from channeling in the two enzyme cascade clustered with 525 QDs. This data supports the kinetic data shown in Figure 3. (**A**) Traces of NAD^+ concentration versus time for the two-enzyme cascade at increasing concentrations of 525 QD with 450 μM PEP. (**B**) Plots of k_{flux} showing initial rates of NAD^+ conversion for the two-enzyme cascade across increasing amounts of 525 QD used in the self-assembly *versus* increasing concentrations of PEP.

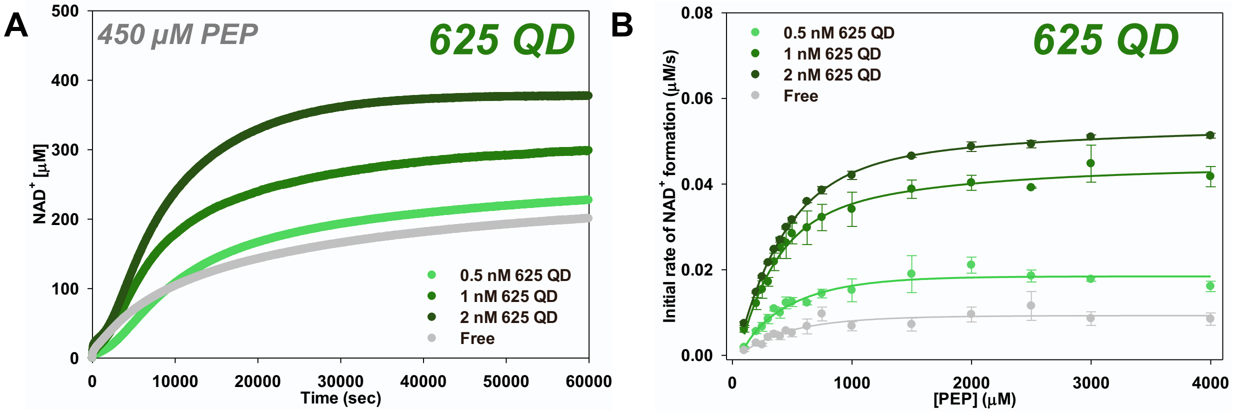

Figure S10. Kinetic enhancement from channeling in the two enzyme cascade clustered with 625 QDs. This data supports the kinetic data shown in Figure 3. (**A**) Traces of NAD^+ concentration versus time for the two-enzyme cascade at increasing concentrations of 625 QD with 450 μM PEP. (**B**) Plots of k_{flux} showing initial rates of NAD^+ conversion for the two-enzyme cascade across increasing amounts of 625 QD used in the self-assembly *versus* increasing concentrations of PEP.

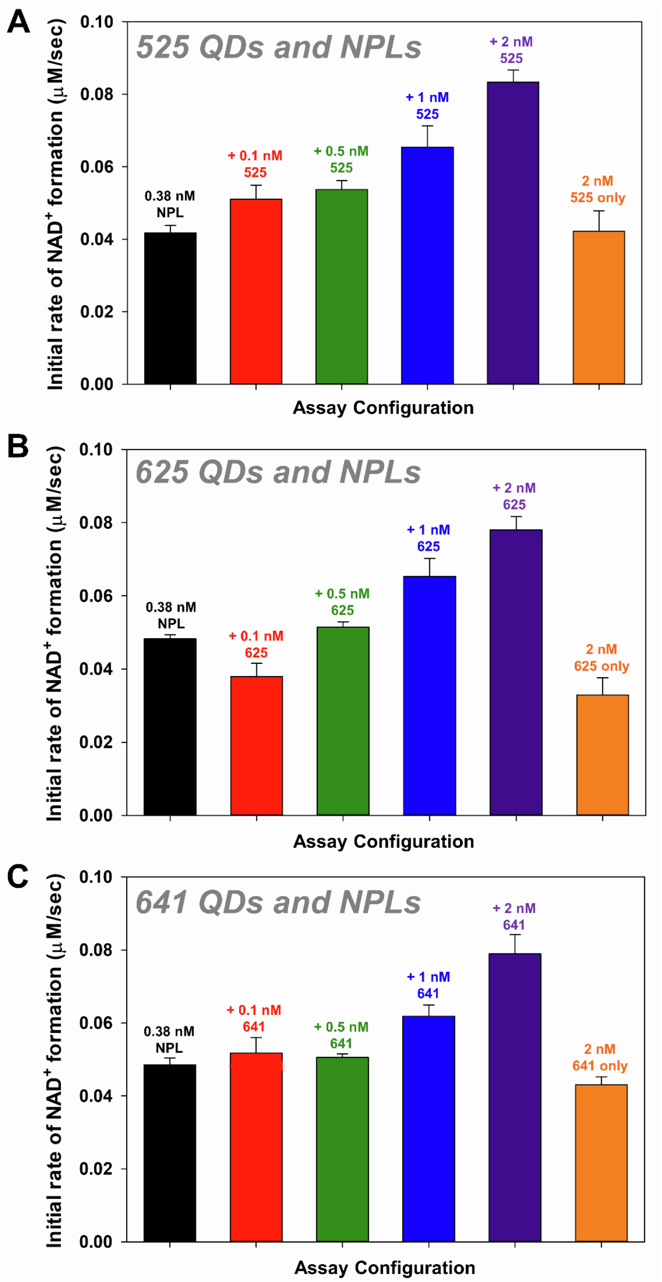

Figure S11. Changes in initial rate at 4000 µM PEP in the two-enzyme cascade as the result of mixed QD-NPL clusters engaged in channeling. This data supports the kinetic data shown in Figure 5. (**A**) Plots of initial rate of NAD^+ conversion for the two-enzyme cascade with 0.375 nM NPL and increasing amounts of 525 QD used in the self-assembly at 4000 µM PEP. (**B**) Plots of initial rate of NAD^+ conversion for the two-enzyme cascade with 0.375 nM NPL and increasing amounts of 625 QD used in the self-assembly at 4000 µM PEP. (**C**) Plots of initial rate of NAD^+ conversion for the two-enzyme cascade with 0.375 nM NPL and increasing amounts of 641 QD used in the self-assembly at 4000 µM PEP. Enzyme concentrations held constant in each assay while nanoparticle concentrations varied. Data points from replicate samples and standard deviations were <15% in all cases.

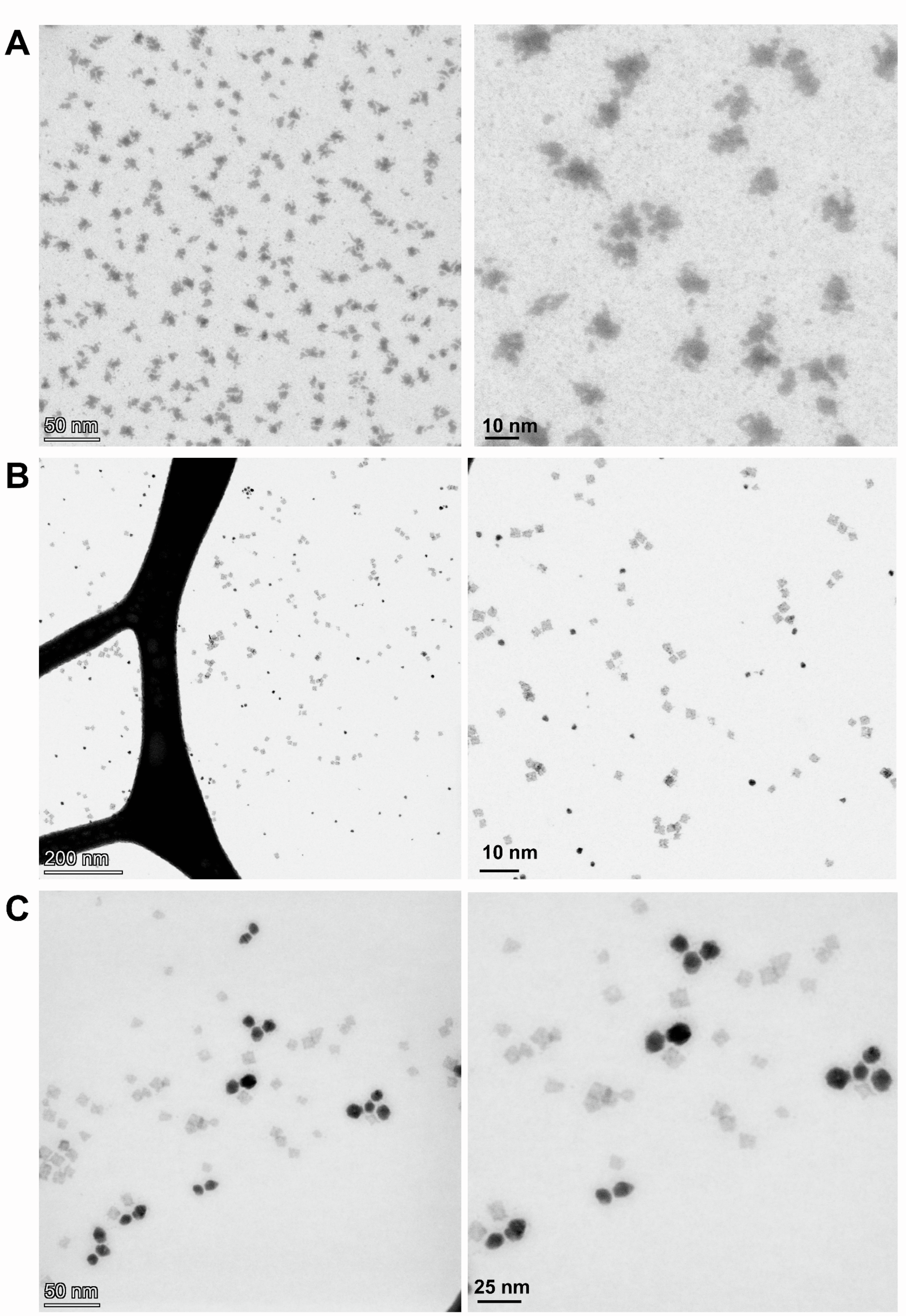

Figure S12. TEM images of mixed NPL systems. This data supports the kinetic data shown in Figure 2B. Samples with NPL and 525 QDs **(A)**, 625 QDs **(B)**, or 641 QDs **(C)**. Conditions: 10 mM HEPES, 0.38 nM NPL, 0.75 nM QD, 40 nM LDH, and 20 nM PyKA.

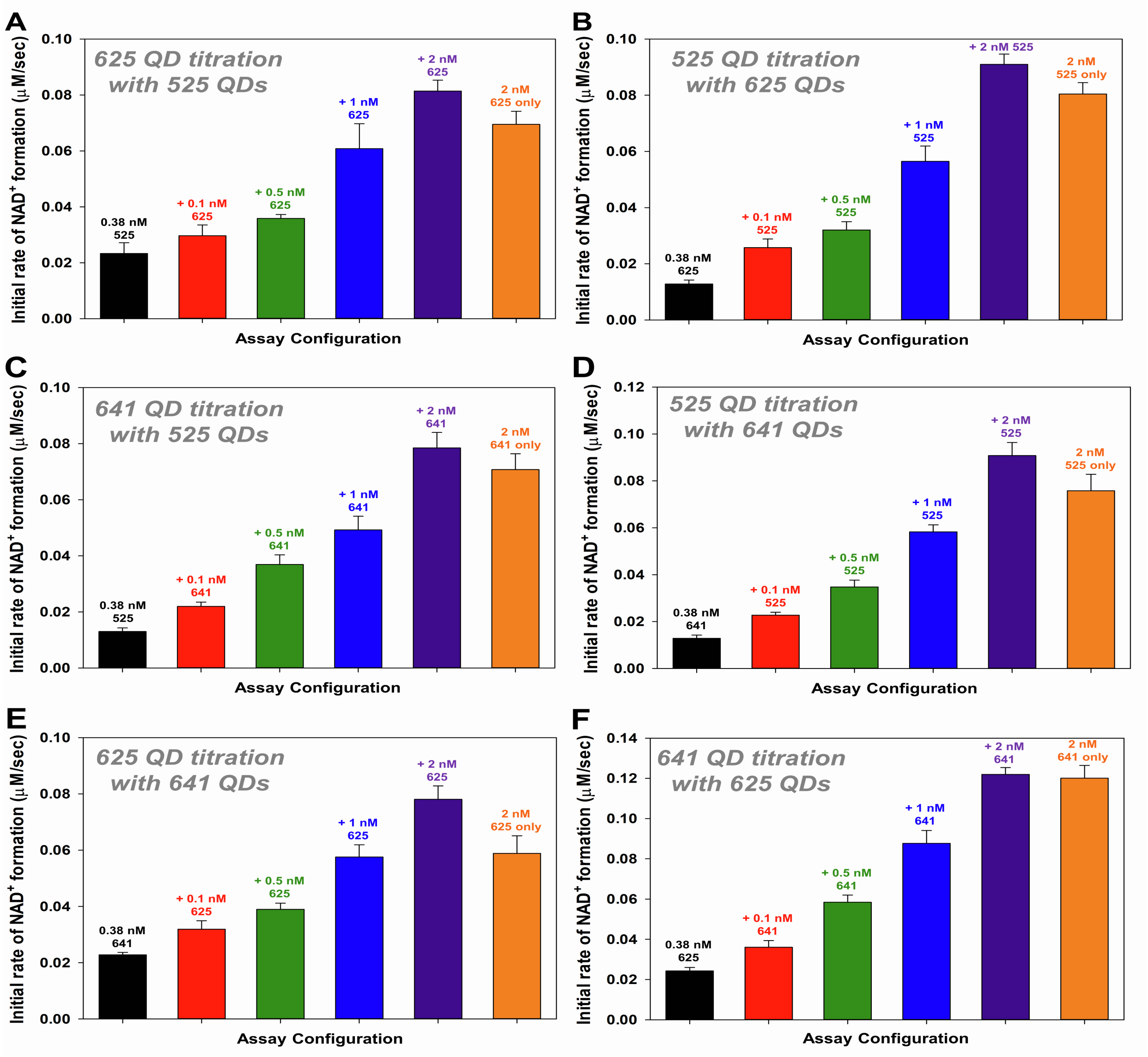

Figure S13. Changes in initial rate at 4000 µM PEP in the two-enzyme cascade as the result of mixed QDs of different sizes. This data supports the kinetic data shown in Figure 4 and Figure 5. (**A**) Plots of initial rate of NAD^+ conversion for the two-enzyme cascade with 0.38 nM 525 QD and increasing amounts of 625 QD used in the self-assembly at 4000 µM PEP. (**B**) Plots of initial rate of NAD^+ conversion for the two-enzyme cascade with 0.38 nM 625 QD and increasing amounts of 525 QD used in the self-assembly at 4000 µM PEP. (**C**) Plots of initial rate of NAD^+ conversion for the two-enzyme cascade with 0.38 nM 525 QD and increasing amounts of 641 QD used in the self-assembly at 4000 µM PEP. (**D**) Plots of initial rate of NAD^+ conversion for the two-enzyme cascade with 0.38 nM 641 QD and increasing amounts of 525 QD used in the self-assembly at 4000 µM PEP. (**E**) Plots of initial rate of NAD^+ conversion for the two-enzyme cascade with 0.38 nM 641 QD and increasing amounts of 625 QD used in the self-assembly at 4000 µM PEP. (**F**) Plots of initial rate of NAD^+ conversion for the two-enzyme cascade with 0.38 nM 625 QD and increasing amounts of 641 QD used in the self-assembly at 4000 µM PEP. Enzyme concentrations held constant in each assay while nanoparticle concentrations varied. Data points from replicate samples and standard deviations were <15% in all cases.

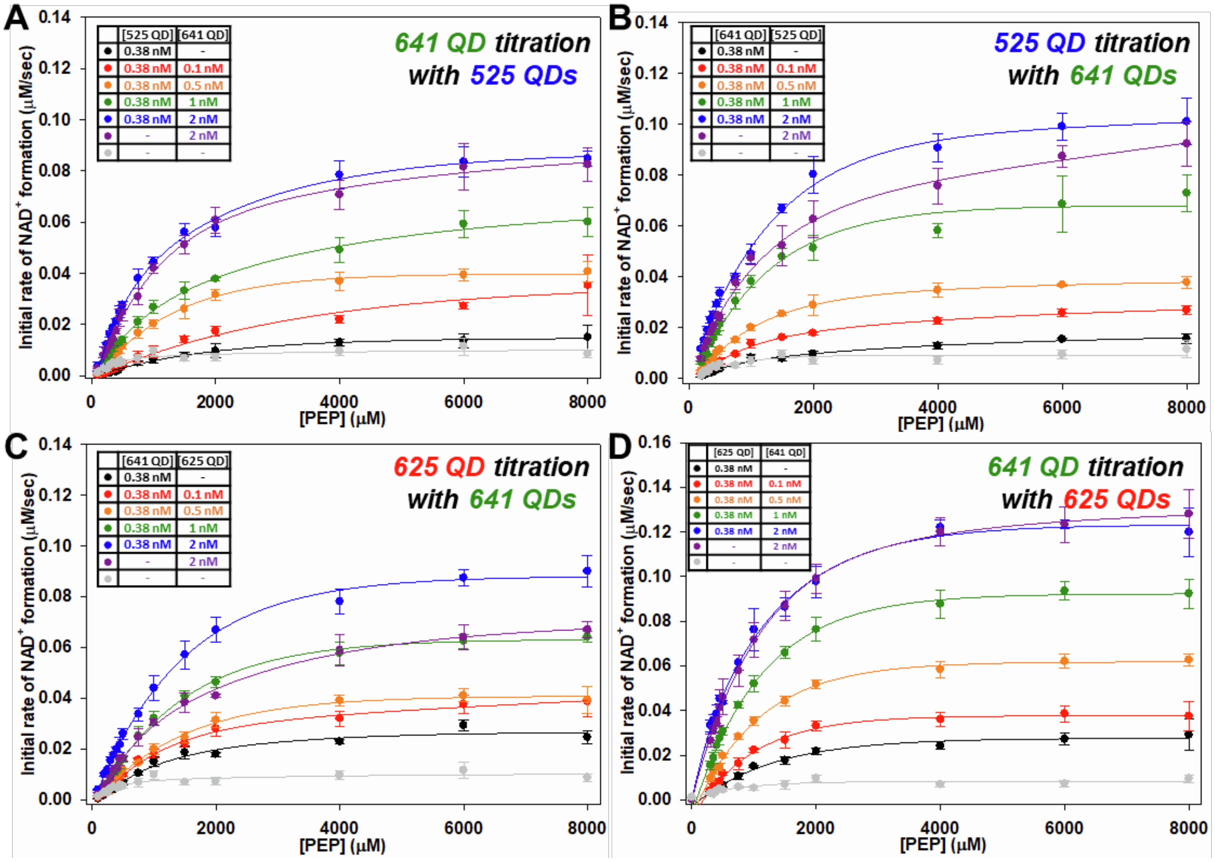

Figure S14. Mixed QD-QD assembly systems. This data supports the kinetic data shown in Figure 5. (A) Plots of k_{flux} showing initial rates of NAD^+ conversion for the two-enzyme cascade with 0.38 nM 525 QD and increasing amounts of 641 QD used in the self-assembly versus increasing concentrations of PEP. **(B)** Plots of k_{flux} showing initial rates of NAD^+ conversion for the two-enzyme cascade with 0.38 nM 641 QD and increasing amounts of 525 QD used in the self-assembly versus increasing concentrations of PEP. **(C)** Plots of k_{flux} showing initial rates of NAD^+ conversion for the two-enzyme cascade with 0.38 nM 641 QD and increasing amounts of 625 QD used in the self-assembly versus increasing concentrations of PEP. **(D)** Plots of k_{flux} showing initial rates of NAD^+ conversion for the two-enzyme cascade with 0.38 nM 625 QD and increasing amounts of 641 QD used in the self-assembly versus increasing concentrations of PEP. Enzyme concentrations held constant in each assay while nanoparticle concentrations varied. Data points from replicate samples and standard deviations were <15% in all cases. Trend lines to aid the eye are included in A-D, these are not necessarily the MM fits.
